# Inferring Tissue-Specific, TLR4-Dependent Type 17 Immune Interactions in Experimental Trauma/Hemorrhagic Shock and Resuscitation Using Computational Modeling

**DOI:** 10.3389/fimmu.2022.908618

**Published:** 2022-05-19

**Authors:** Ashti M. Shah, Ruben Zamora, Sebastian Korff, Derek Barclay, Jinling Yin, Fayten El-Dehaibi, Timothy R. Billiar, Yoram Vodovotz

**Affiliations:** ^1^Department of Surgery, University of Pittsburgh, Pittsburgh, PA, United States; ^2^Center for Inflammation and Regeneration Modeling, McGowan Institute for Regenerative Medicine, Pittsburgh, PA, United States; ^3^Center for Systems Immunology, University of Pittsburgh, Pittsburgh, PA, United States

**Keywords:** trauma, hemorrhagic shock, inflammation, systems biology, computational biology, Toll-like 4 receptor

## Abstract

Trauma/hemorrhagic shock followed by resuscitation (T/HS-R) results in multi-system inflammation and organ dysfunction, in part driven by binding of damage-associated molecular pattern molecules to Toll-like Receptor 4 (TLR4). We carried out experimental T/HS-R (pseudo-fracture plus 2 h of shock followed by 0-22 h of resuscitation) in C57BL/6 (wild type [WT]) and TLR4-null (TLR4^-/-^) mice, and then defined the dynamics of 20 protein-level inflammatory mediators in the heart, gut, lung, liver, spleen, kidney, and systemic circulation. Cross-correlation and Principal Component Analysis (PCA) on data from the 7 tissues sampled suggested that TLR4^-/-^ samples express multiple inflammatory mediators in a small subset of tissue compartments as compared to the WT samples, in which many inflammatory mediators were localized non-specifically to nearly all compartments. We and others have previously defined a central role for type 17 immune cells in human trauma. Accordingly, correlations between IL-17A and GM-CSF (indicative of pathogenic Th17 cells); between IL-17A and IL-10 (indicative of non-pathogenic Th17 cells); and IL-17A and TNF (indicative of memory/effector T cells) were assessed across all tissues studied. In both WT and TLR4^-/-^ mice, positive correlations were observed between IL-17A and GM-CSF, IL-10, and TNF in the kidney and gut. In contrast, the variable and dynamic presence of both pathogenic and non-pathogenic Th17 cells was inferred in the systemic circulation of TLR4^-/-^ mice over time, suggesting a role for TLR4 in efflux of these cells into peripheral tissues. Hypergraph analysis – used to define dynamic, cross compartment networks – in concert with PCA-suggested that IL-17A was present persistently in all tissues at all sampled time points except for its absence in the plasma at 0.5h in the WT group, supporting the hypothesis that T/HS-R induces efflux of Th17 cells from the circulation and into specific tissues. These analyses suggest a complex, context-specific role for TLR4 and type 17 immunity following T/HS-R.

## Introduction

Trauma, which often co-occurs with severe hemorrhage (1), is one of the leading causes of death and disability worldwide (2). Patients that survive their initial injuries and hypovolemic shock often undergo organ dysfunction that is associated with, and likely driven by, multi-focal inflammation (3, 4). In the context of human trauma, it is often difficult to discern the dynamic flow of inflammation across tissues and organs, with the systemic circulation being the main compartment that is amenable to interrogation. Despite this limitation, an extensive body of literature has documented the impact of trauma/hemorrhage on immune cells (5, 6), inflammatory mediators (7–9), and a large array of cell-derived proteins and metabolites (10, 11) in the systemic circulation. Among a multitude of findings, these studies have suggested a potential role for type 17 immune responses following traumatic injury (7, 12, 13).

A key, unanswered question remains that of how trauma/hemorrhage impact immuno-inflammatory responses in various organs and the systemic circulation. We have recently begun to address this question in the simpler context of experimental endotoxemia in mice, utilizing computational methods aimed at defining the temporal hierarchy of the cross-tissue progression of inflammation, dynamic networks of inflammation, and the hallmarks of pathological systemic spillover of inflammation (14, 15). These studies also implicated type 17 immune responses in the spatiotemporal elaboration of inflammation (15).

The inflammatory response to both traumatic injury and sepsis/endotoxemia involves Toll-like receptor-4 (TLR4). In the context of sepsis, TLR4 transduces signals from pathogen-derived molecular pattern (PAMP) molecules such as endotoxin/lipopolysaccharide (LPS) (16), whereas in the setting of trauma TLR4 mediates signals from damage-associated molecular pattern (DAMP) molecules such as high-mobility group box-1 (HMGB1) (17). Our prior studies on modeling the spatiotemporal dynamics of LPS-induced inflammation suggested distinct, tissue- and time-specific differences in wild type (WT) C57BL/6 mice as compared to TLR4-deficient (TLR4^-/-^) mice (14, 15).

In the present study, our goal was to interrogate the spatiotemporal dynamics of inflammation in the context of experimental trauma/hemorrhagic shock, and the impact of resuscitation on these dynamics. We also sought to define the impact of TLR4 deficiency. Our prior data-driven modeling studies in both experimental (18) and clinical (12, 19–21) settings of trauma/hemorrhage leveraged Principal Component Analysis (PCA) (7, 18, 22), cross-correlation analysis (14), and dynamic network discovery algorithms (4, 8, 15). Here, we utilized PCA along with an emerging multi-dimensional network analysis approach known as hypergraphs (23–26) to define novel DAMP/TLR interactions.

## Materials and Methods

### Experimental Model of T/HS-R in Mice

All procedures involving animals complied with the regulations regarding the care and use of experimental animals published by the National Institutes of Health and were approved by the Institutional Animal Care and Use Committee of the University of Pittsburgh. Male TLR4^+/+^ C57BL/6 mice were purchased from Jackson Laboratory (Bar Harbor, ME, USA). TLR4-null (TLR4^-/-^) mice were bred at the University of Pittsburgh animal facility on a C57BL/6 background (27). Animals were allowed access to rodent chow and water *ad libitum* and used at the age of 8-12 weeks. Both wild type (WT) and TLR4^-/-^ mice were randomly assigned to one of three experimental groups: Control (animals were sacrificed directly after anesthesia to obtain physiological baseline levels, n=4-5), HS (animals were subjected to pseudo-fracture followed by pressure controlled hemorrhagic shock, n=4), and HS/R (animals were subjected to pseudo-fracture and hemorrhagic shock followed by 30 min, 1h, 4h, and 22h resuscitation, n=4 each), as previously described (28). At different time-points, the animals were anesthetized with isoflurane (0.25-2% as needed), cardiac puncture was performed, blood was collected into heparinized tubes, and then centrifuged to obtain plasma; the mice were then euthanized by cervical dislocation while under anesthesia. Mice were then perfused with ice-cold PBS followed by RNALater™ (Thermo Fisher Scientific, Waltham, MA), which we have previously shown to be a preservation method compatible with Luminex™ analysis and equivalent to flash-freezing in liquid nitrogen (29). A small section (approx. 100 mg) of each tissue (liver [left lobe], heart, gut [terminal ileum], lung [left lobe], spleen, and kidney [left]) was collected and stored at -80°C until analysis. Total protein isolation and determination was done as described previously (30).

### Assay of Inflammatory Mediators

Mouse inflammatory mediators were measured using a Luminex™ 100 IS apparatus (Luminex, Austin, TX) and the 20-plex mouse cytokine bead kit (MCYTO-70K-20, Millipore, Burlington, MA) as per manufacturer’s specifications. The antibody bead kit included: Granulocyte-Macrophage Colony-Stimulating Factor (GM-CSF), Interferon-γ (IFN-γ), Interleukin (IL)-1α, IL-1β, IL-2, IL-4, IL-5, IL-6, IL-10, IL-12p40, IL-12p70, IL-13, IL-17A, Interferon-γ-inducible Protein 10 (IP-10/CXCL10), Keratinocyte-derived Cytokine (KC/CXCL1), Monocyte Chemoattractant Protein-1 (MCP-1/CCL2), Monokine induced by Interferon-γ (MIG/CXCL9), Macrophage Inflammatory Protein-1α (MIP-1α/CCL3), Tumor Necrosis Factor-α (TNF), and Vascular Endothelial Growth Factor (VEGF). The final mediator concentrations are expressed in pg/ml for plasma samples, and in pg/mg total protein for tissue samples. Experimental data are presented as mean ± SEM.

### Statistical and Computational Analyses

*Two-Way Analysis of Variance* (ANOVA) was carried out to analyze the time-dependent changes in inflammatory mediators in C57BL/6 (wild type, WT) vs. TLR4^-/-^ mice in all organs as well as in plasma, using SigmaPlot (Systat Software, San Jose, CA).

*Heatmaps and Spearman’s correlation* carried out to measure the strength of the association between the Luminex™ data for two different mediators were generated using MetaboAnalyst (https://www.metaboanalyst.ca) (31, 32).

*Principal component analysis* (PCA) was carried out to identify the inflammatory mediators that contributed the most to the overall variance of the response in all organs as well as in plasma of both wild type and TLR4^-/-^ mice with and without HS and HS/R as described above. The algorithm employed was implemented using MATLAB^®^ software (The MathWorks, Inc., Natick, MA) and has been reported previously (18).

*Hypergraphs* are a computational tool to model how inflammatory mediators move between tissues across time. This form of network analysis can be used to represent edges that connect two or more vertices (23–26). Here, we created a set of hypergraphs to model the inflammatory mediators sampled in the WT and TLR4^-/-^ experimental groups described above. We designated inflammatory mediators as edges and tissue compartments as nodes. As such, we utilized the following nodes/tissue compartments: liver, spleen, gut, lung, kidney, heart, and plasma. A hypergraph was created for each time point at which a tissue sample was drawn (CTRL, HS, HS/R at 0.5h, 1h, 4h, and 22h). An edge is drawn around one or more nodes when the concentration of the cytokine in the tissue/plasma is > 0. The resultant hypergraphs depict which inflammatory mediators are located within a given set of tissues at specific time point. Hypergraphs were created using the open-source package HyperNetX (https://github.com/pnnl/HyperNetX) (33).

To add quantitative analysis to the hypergraph visualizations, we utilized s-betweenness centrality and edge distribution to characterize network complexity. S-betweenness centrality is a measure of the number of times an edge lies on the shortest path between other edges. In essence, s-betweenness centrality is a measure of the extent to which an edge, or inflammatory mediator, acts as a bridge between nodes, or tissue compartments. Here, we focused on 1-betweenness centrality which captures edges that connect one or more nodes. The second quantitative metric used was edge distribution, which simply tallies the number of edges that surround 1, 2, and 3 nodes, respectively. Such a metric quantifies the extent to which inflammatory mediators are grossly disseminated throughout the body.

## Results

### Differential Dynamic Expression of Inflammatory Mediators in Multiple Tissues of WT vs. TLR4^-/-^ Mice

**Figure 1:**
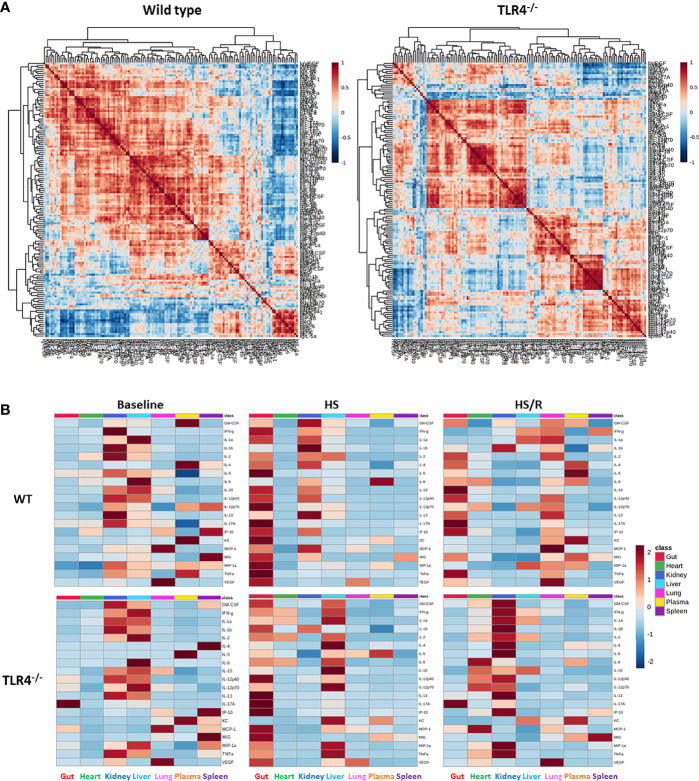
Differential inflammatory correlation patterns in WT vs. TLR4^-/-^ mice. **(A)** Heatmaps show Spearman Rank Correlation patterns using all experimental data (Ctrl + HS + HS/R) in wild type vs. TLR4^-/-^ mice as described in *Materials and Methods*. **(B)** Heatmaps show the average concentration values for each mediator in each experimental group (Ctrl-Baseline, HS, HS/R in wild type vs. TLR4^-/-^ mice as described in Materials and Methods.

We first performed Spearman Rank correlation analysis using all experimental data of WT vs. TLR4^-/-^ mice to visualize differences between WT and TLR4^-/-^ mice at baseline (Control), in response to hemorrhagic shock, and following resuscitation. This analysis suggested a generally more robust inflammatory response in WT as compared to TLR4^-/-^ mice (**Figure 1A**). To compare inflammatory profiles of WT vs. TLR4^-/-^mice for the three experimental conditions (Ctrl, HS, and HS/R), we generated 3 individual heatmaps using the average concentration values for each mediator in each group (**Figure 1B**). This analysis suggested that 1) the main tissues in which inflammation evolved following T/HS-R in both WT and TLR4^-/-^ were the gut > kidney > liver > lung, and, to a lesser degree, the systemic circulation; 2) that in both WT and TLR4^-/-^ mice HS resulted in a major impact on the gut, which persisted in WT mice following resuscitation but that was greatly reduced in TLR4^-/-^ mice; and 3) that baseline inflammatory responses in the kidney were reduced following hemorrhage and further reduced following resuscitation in WT mice, while decreasing following hemorrhage but rising substantially following resuscitation in TLR4^-/-^ mice. This analysis pointed to a complex, context-specific role for TLR4 following T-HS/R.

To determine the impact TLR4 status over time, we next carried out Two-Way ANOVA (**Supplementary Figure 1**). Comparison of time-courses (wild type vs. TLR4^-/-^) showed statistically significant changes in several inflammatory mediators (n) as follows: spleen (n=7 [GM-CSF, IL-1α, IL-5, IL-10, IP-10, KC, MIG]), kidney (n=6 [IL-1α, IL-4, IL-5, IL-12p70, KC, MIP-1α]), plasma (n=5 [KC, MCP-1, MIG, MIP-1α, TNFα]), heart (GM-CSF), liver (KC), lung (IL-4), and gut (IL-10). Notably, the absence of TLR4 was not associated simply with reduced levels of inflammatory mediators; rather, we observed complex dynamics in which certain mediators were higher in WT or in TLR4^-/-^ mice in certain tissues at certain time points (**Supplementary Figure 1**).

### Hypergraph Analysis Defines Distinct Multi-Organ Inflammation in WT vs. TLR4^-/-^ Mice

**Figure 2:**
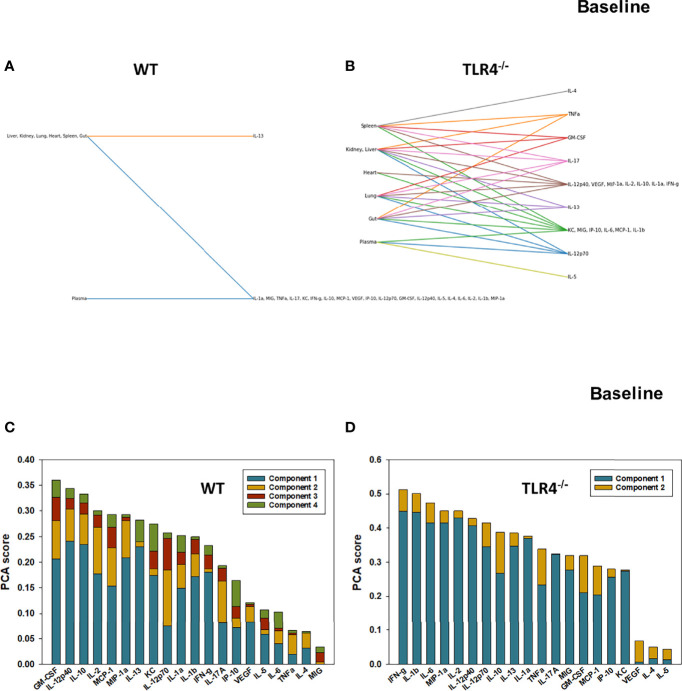
Hypergraphs and Principal Component Analysis suggest differential baseline inflammatory responses in WT and TLR4^-/-^ mice. Plasma and tissue samples from heart, lung, liver, gut, spleen, and kidney were collected from untreated (Ctrl) WT and TLR4^-/-^ mice and analyzed using Luminex™ as described in Materials and Methods. Panels **(A, B)** show the hypergraphs and Panels **(C, D)** show the PCA results (70% variance) in WT and TLR4^-/-^ mice, respectively.

**Figure 3:**
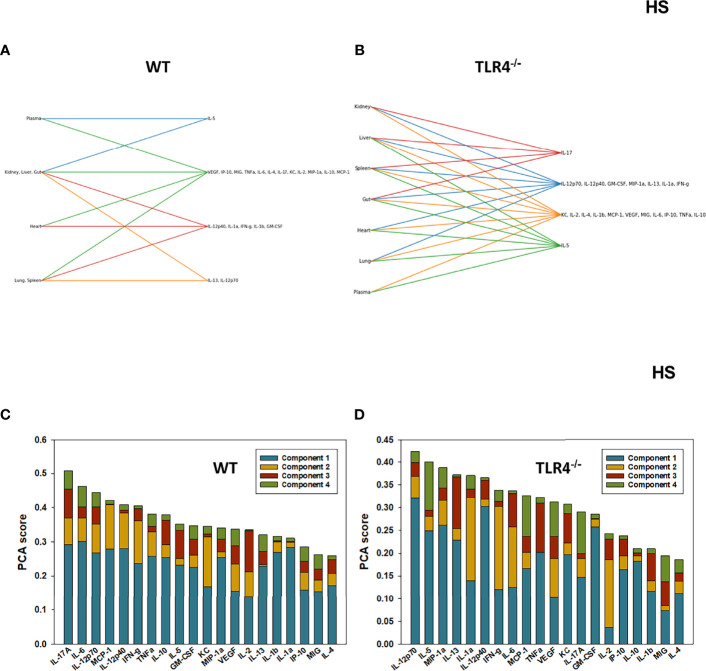
*Hypergraphs and Principal Component Analysis of inflammatory mediators from WT and TLR4^-/-^* mice *subjected to HS.* Plasma and tissue samples from heart, lung, liver, gut, spleen, and kidney were collected from WT and TLR4^-/-^ mice subjected to HS and analyzed using Luminex™ as described in Materials and Methods. Panels **(A, B)** show the hypergraphs and Panels **(C, D)** show the PCA results (70% variance) in WT and TLR4^-/-^ mice, respectively.

**Figure 4:**
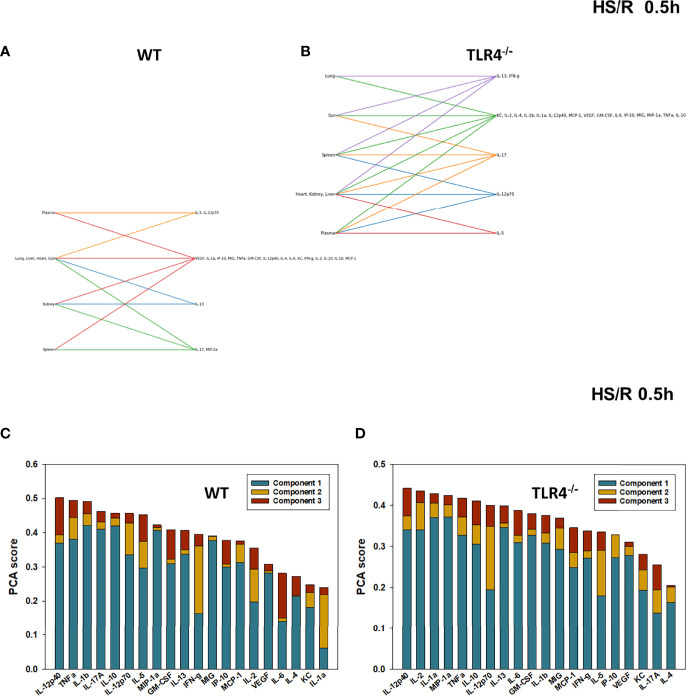
Hypergraphs and Principal Component Analysis of inflammatory mediators from WT and TLR4^-/-^ mice subjected to HS/R (0.5h). Plasma and tissue samples from heart, lung, liver, gut, spleen, and kidney were collected from WT and TLR4^-/-^ mice subjected to HS/R (0.5h) and analyzed using Luminex™ as described in Materials and Methods . Panels **(A, B)** show the hypergraphs and Panels **(C, D)** show the PCA results (70% variance) in WT and TLR4^-/-^ mice, respectively.

**Figure 5:**
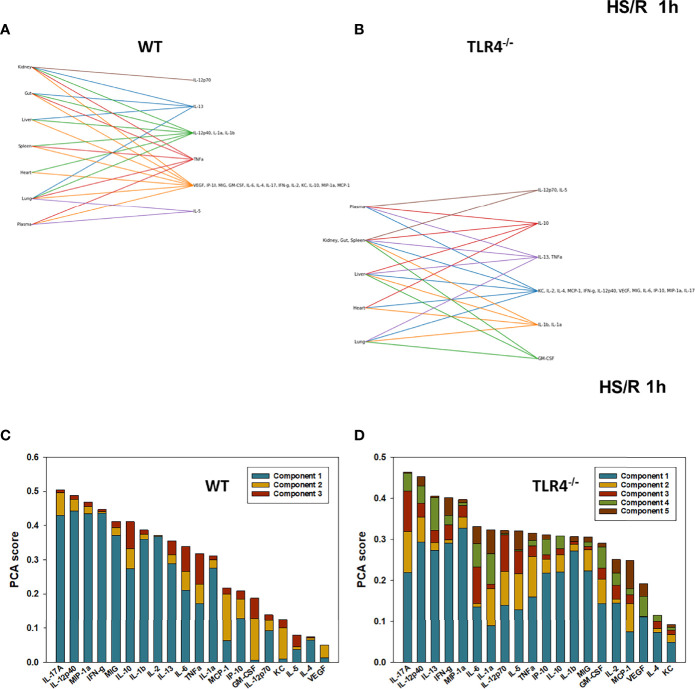
Hypergraphs and Principal Component Analysis of inflammatory mediators from WT and TLR4^-/-^ mice subjected to HS/R (1h). Plasma and tissue samples from heart, lung, liver, gut, spleen, and kidney were collected from WT and TLR4^-/-^ mice subjected to HS/R (1h) and analyzed using Luminex™ as described in Materials and Methods. Panels **(A, B)** show the hypergraphs and Panels **(C, D)** show the PCA results (70% variance) in WT and TLR4^-/-^ mice, respectively.

**Figure 6:**
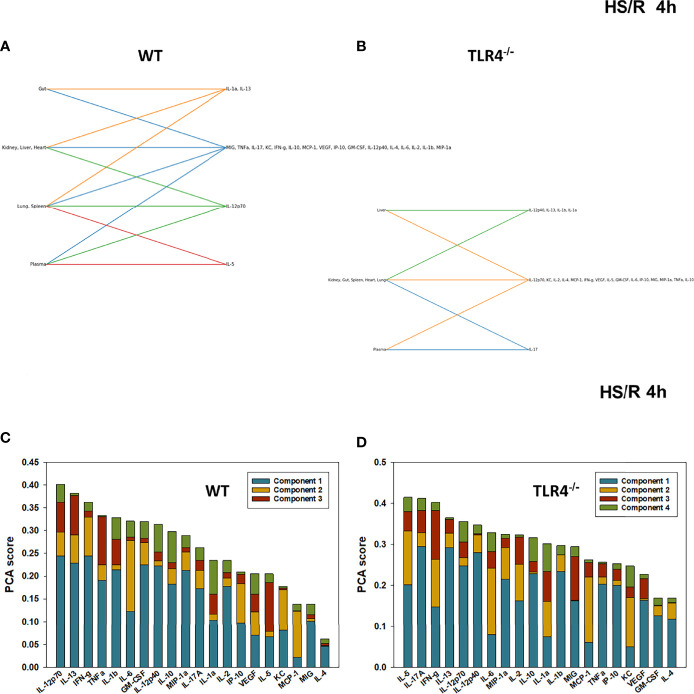
Hypergraphs and Principal Component Analysis of inflammatory mediators from WT and TLR4^-/-^ mice subjected to HS/R (4h). Plasma and tissue samples from heart, lung, liver, gut, spleen, and kidney were collected from WT and TLR4^-/-^ mice subjected to HS/R (4h) and analyzed using Luminex™ as described in Materials and Methods. Panels **(A, B)** show the hypergraphs and Panels **(C, D)** show the PCA results (70% variance) in WT and TLR4^-/-^ mice, respectively.

**Figure 7:**
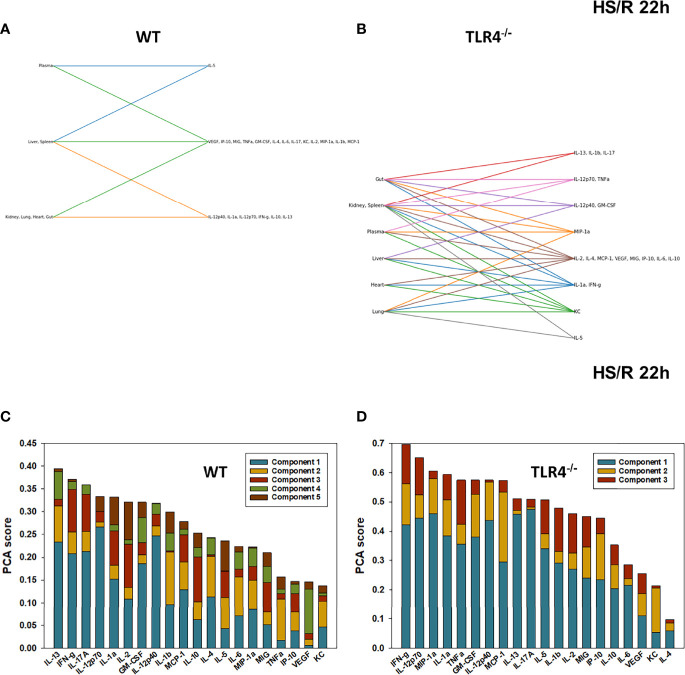
Hypergraphs and Principal Component Analysis of inflammatory mediators from WT and TLR4^-/-^ mice subjected to HS/R (22h). Plasma and tissue samples from heart, lung, liver, gut, spleen, and kidney were collected from WT and TLR4^-/-^ mice subjected to HS/R (22h) and analyzed using Luminex™ as described in Materials and Methods. Panels **(A, B)** show the hypergraphs and Panels **(C, D)** show the PCA results (70% variance) in WT and TLR4^-/-^ mice, respectively.

We have reported previously that the expression of TLR4 impacts dynamic networks of inflammation induced by LPS in mice (14, 15). We therefore hypothesized that the expression of TLR4 impacts the cross-compartment spread of inflammation, and thus compared the dynamic evolution of inflammation in WT and TLR4^-/-^ mice following T/HS-R. While dynamic network inference algorithms such as Dynamic Network Analysis (18) and Dynamic Bayesian Network (DyBN) inference (20) have both proven useful in identifying novel features of systemic inflammation following trauma/hemorrhage, as well as for defining cross-tissue interactions in other context of inflammation (34), these methods are not designed explicitly for multi-dimensional analysis. Emerging hypergraph methods (23–26) hold the potential for this type of analysis, and therefore we carried out a hypergraph analysis of the multi-tissue dataset at each time point (**Figures 2**–**7**). Prior to injury, despite overall low levels of inflammatory mediators in general, WT mice (**Figure 2A**) expressed a variety of cytokines and chemokines in fewer organs with more mediators present in the systemic circulation vs. TLR4^-/-^ mice (**Figure 2B**), suggesting that TLR4 deficiency mitigated an inherent tendency for pathological systemic inflammation in C57BL/6 mice. Notably, after 2h of hemorrhagic shock, overall tissue distribution of inflammatory mediators was fairly similar in WT and TLR4^-/-^ mice (**Figure 3**). This overall compartmental similarity of inflammation between WT and TLR4^-/-^ mice was preserved following resuscitation at 0.5h (**Figure 4**), 1h (**Figure 5**), and 4h (**Figure 6**). By 22h following resuscitation, the qualitative pattern of tissue distribution of inflammatory mediators began to resemble that observed prior to the induction of trauma/hemorrhage (**Figure 7**).

Taken together, these results suggest that TLR4 affects baseline inflammatory responses across multiple organs and the systemic circulation. This difference is blurred following severe stress, since inflammation triggered by trauma/hemorrhage and initially following resuscitation was at least qualitatively similar across multiple organs and the systemic circulation in both WT and TLR4^-/-^ mice. By approximately 24h following resuscitated shock, the respective mouse strains appeared to recover toward their original, genetically encoded state.

Despite the overall qualitative similarity, there were different groupings of inflammatory mediators in distinct organ clusters at each time point in WT vs. TLR4^-/-^ mice (**Supplementary Table 1**), suggesting organ-specific roles for TLR4 in regulating post T/HS-R inflammation. Notably, IL-17A was present persistently in all tissues at all sampled time points except for its absence in the systemic circulation following 0.5h of resuscitation in the WT group. Interestingly, TNF was found in all tissues in TLR4^-/-^ mice from 0-4h following resuscitation, but was no longer expressed in the liver, lung, and heart by 22h.

### Principal Component Analysis Defines Key Inflammatory Mediators at Baseline and Following Hemorrhagic Shock and Resuscitation in WT and TLR4^-/-^ Mice

We next utilized Principal Component Analysis (70% variance) to better define the principal mediators associated with inflammation at baseline, following hemorrhage, and over time post-resuscitation in WT and TLR4^-/-^ mice. At baseline, inflammation in WT mice was characterized by 4 principal components involving GM-CSF, IL-12p40, and IL-10 among a multitude of other mediators (**Figure 2C**), while in TLR4^-/-^ mice this response was characterized by 2 principal components and IFN-γ and IL-1β along with multiple other mediators. This supports the concept that WT and TLR4^-/-^ differ in the baseline inflammatory propensities. Following hemorrhagic shock, inflammation in WT mice was characterized by 4 principal components involving IL-17A, IL-6, and IL-12p70 among a multitude of other mediators (**Figure 3C**), while in TLR4^-/-^ mice this response was also characterized by 4 principal components and the mediators IL-12p70, IL-5, and MIP-1α among others (**Figure 3D**). This supports the hypothesis that stress in the form of hemorrhagic shock blurs some of the baseline differences between WT and TLR4^-/-^ mice.

The relative similarity between WT and TLR4^-/-^ persisted following resuscitation for 0.5-4h (compare **Figures 4C** vs **4D**, **5C** vs **5C**, and **6C** vs **6C**, respectively). By 22h post-resuscitation, inflammation in WT mice was characterized by 5 principal components involving IL-13, IFN-γ, and IL-17A among others (**Figure 7C**), while in TLR4^-/-^ mice this response was also characterized by 3 principal components and the mediators IFN-γ and IL-12p70 among others (**Figure 7D**), suggesting a return toward baseline inter-strain differences in inflammation.

Taken together, these analyses support the conclusion that TLR4 affects baseline inflammatory responses across multiple compartments, that these differences are blurred somewhat following hemorrhagic shock and resuscitation, and that, with time, these baseline differences re-emerge. Furthermore, the predominance of IL-17A suggests an important role for type 17/type 3 immunity, while the presence of IL-12p40/p70 suggests a role for dendritic cells.

### Hypergraph Metrics Yield Insights Into Cross-Compartment Inflammation Following Hemorrhagic Shock and Resuscitation in WT and TLR4^-/-^ Mice

**Figure 8:**
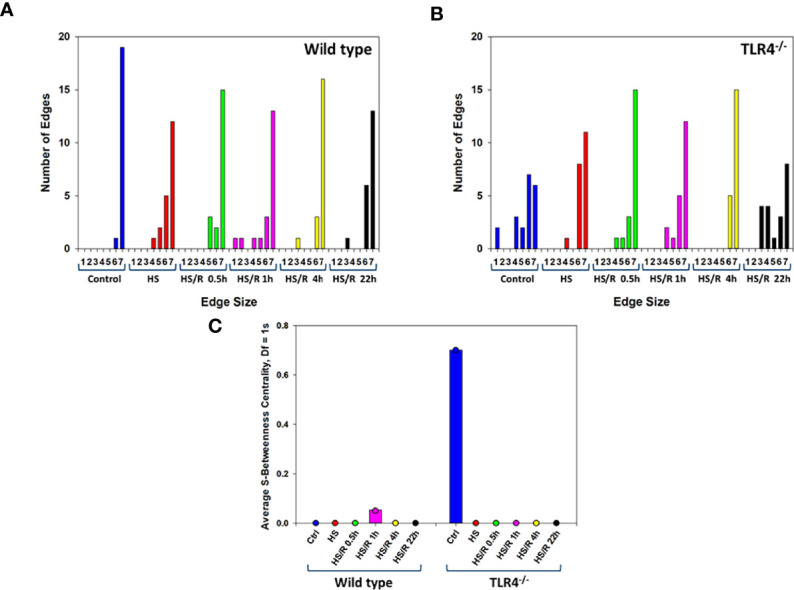
Hypergraph metrics demonstrate differential tissue distribution of inflammatory mediators from WT and TLR4^-/-^ mice. Plasma and tissue samples from heart, lung, liver, gut, spleen, and kidney were collected from Ctrl WT and TLR4^-/-^ mice or mice subjected to HS and HS/R (0.5-22h) and analyzed using Luminex™ as described in Materials and Methods. Panels **(A, B)** show the Hypergraph Edge Distribution analysis in WT and TLR4^-/-^ mice, respectively. Panel **(C)** shows the average S-Betweenness Centrality (Df=1) for each hypergraph as indicated in WT and TLR4^-/-^ mice, respectively.

We next sought to better define the compartment-specific role of TLR4 in modulating the spread of inflammation following T/HS-R. Hypergraph Edge Distribution analysis suggested that WT mice had a more robust trans-compartmental inflammatory response at baseline, prior to T/HS-R, as compared to TLR4^-/-^ mice (**Figure 8**). After hemorrhage and prior to resuscitation, WT mice exhibited a decrease in the number of inflammatory mediators there were present in all 7 tissue compartments. By t=1h after resuscitation, the WT group began to express inflammatory mediators in specific tissue compartments as indicated by the bars in the graph, spanning an edge size from 1 to 7. By 22h of resuscitation, the WT group exhibited a generally more limited edge distribution ranging from 6-7 (**Figure 8A**).

In contrast, at pre-hemorrhage baseline, TLR4^-/-^ mice expressed distinct subsets of inflammatory mediators in defined tissue compartments compared to the WT group (**Figure 8B**). The tissue-specific inflammatory responses of TLR4^-/-^ mice to hemorrhage alone were fairly similar to those of WT mice. After resuscitation, TLR4^-/-^ mice exhibited the greatest trans-compartmental inflammation at t = 0.5h and t = 4h, with edge distributions spanning the range of 1-7. By 22h of resuscitation, the edge distribution of TLR4^-/-^ mice was similar to the edge distribution of these mice at baseline, suggesting resolution of cross-compartment inflammation (**Figure 8B**).

As detailed in **Supplementary Table 1**, this homology indicates a similar trans-compartmental distribution of inflammatory mediators in the WT and TLR4^-/-^. Following hemorrhagic shock as well as at 4h and 22h post-resuscitation, the most apparent difference between WT and TLR4^-/-^ mice was that the TLR4^-/-^ mice expressed IL-5 in the spleen, lung, and gut, while WT mice do not. Overall, WT mice expressed a greater number of inflammatory mediators across all compartments and all time points, causing the deceptively different appearing hypergraph structure. However, the graph structures are more similar than different and only differ by more than 50% of the mediators (10/20 inflammatory mediators) in any compartment at baseline.

S-Betweenness centrality, an indicator of the strength by which nodes are connected by a given edge (33) – and inferred as the degree to which inflammation is coordinated – was the greatest at 1h of resuscitation in WT mice. In contrast, S-betweenness centrality was greatest at baseline (Ctrl) in TLR4^-/-^ mice (**Figure 8C**). This result suggests that there may have been a more coordinated inflammatory response to T-HS/R in WT mice, but not so in TLR4^-/-^ mice. In aggregate, our analyses suggest that TLR4^-/-^ mice tend to display an inflammatory response that is more diffusely distributed across multiple tissues as compared to WT mice, which display a more restricted tissue distribution pattern of inflammatory mediators.

### Inferred Differential Th17 Immune Dynamics in WT vs. TLR4^-/-^ Mice

The cross-correlation and hypergraph analyses pointed to IL-17A in the context of spatiotemporal spread of inflammation following T/HS-R predominantly in WT but also in TLR4^-/-^ mice, in line with our prior studies in trauma patients (7, 12). Interleukin-17A can be produced by a variety of cell types, including Th17 cells, innate lymphoid cells, γδ T cells, and both CD4^+^ and CD8^+^ effector/memory T cells (35–37). Kuchroo et al. have described a sub-population of Th17 cells known as pathogenic Th17 cells, which are implicated in driving pathological inflammatory processes. Pathogenic Th17 cells are characterized by the co-expression of IL-17A and GM-CSF; a reciprocal, non-inflammatory Th17 cell subset expresses IL-17A and IL-10 (38). Furthermore, CD4^+^/CD8^+^ effector/memory T cells express IL-17A and TNF (39, 40). We have previously utilized Spearman rank correlation analysis of IL-17A vs. GM-CSF, IL-10, or TNF to infer the presence of these three cell subsets (7, 12, 15), with positive and negative correlations being interpreted as increases or decreases, respectively, in these Th17 cell subsets.

We therefore carried out a similar analysis to determine the potential presence of these cells in distinct tissues of WT and TLR4^-/-^ mice. As shown in **Table 1**, pathogenic Th17 cells were inferred in the heart and kidney of WT mice and in the heart, gut, and kidney of TLR4^-/-^ mice. We observed nearly statistically significant Spearman correlations for pathogenic Th17 cells in the lung and gut of WT mice and in the systemic circulation of TLR4^-/-^ mice. Non-pathogenic Th17 cells were inferred in the heart, gut, and kidney of WT mice and in the heart, liver, gut, kidney, and systemic circulation of TLR4^-/-^ mice (**Table 1**).

**Table 1:** Spearman Rank Correlations (IL-17A vs. GM-CSF, IL-10 and TNFa) in WT and TLR4-/- mice.

WT	r values	P values		TLR4^-/-^	r values	P values
**Plasma**	**IL-17A**	** **		**Plasma**	**IL-17A**	** **
**GM-CSF**	**-0.150**	0.475		**GM-CSF**	0.420	0.052
**IL-10**	**-0.085**	0.688		**IL-10**	**0.533**	**0.011**
**TNFα**	**-0.379**	0.061		**TNFα**	0.105	0.641
** **				** **		
**Heart**	**IL-17A**			**Heart**	**IL-17A**	
**GM-CSF**	**0.698**	**1.038E-04**		**GM-CSF**	**0.516**	**0.014**
**IL-10**	**0.906**	**4.458E-10**		**IL-10**	**0.461**	**0.031**
**TNFα**	0.376	0.064		**TNFα**	**0.5154**	**0.014**
** **				** **		
**Liver**	**IL-17A**			**Liver**	**IL-17A**	
**GM-CSF**	0.312	0.129		**GM-CSF**	0.313	0.157
**IL-10**	0.228	0.273		**IL-10**	**0.488**	**0.021**
**TNFα**	**0.588**	**0.002**		**TNFα**	0.379	0.082
** **				** **		
**Lung**	**IL-17A**			**Lung**	**IL-17A**	
**GM-CSF**	0.395	0.051		**GM-CSF**	0.289	0.192
**IL-10**	0.082	0.698		**IL-10**	**-0.158**	0.481
**TNFα**	**0.597**	**0.002**		**TNFα**	**-0.087**	0.7
** **				** **		
**Gut**	**IL-17A**			**Gut**	**IL-17A**	
**GM-CSF**	0.392	0.053		**GM-CSF**	**0.632**	**0.002**
**IL-10**	**0.525**	**0.007**		**IL-10**	**0.513**	**0.015**
**TNFα**	**0.663**	**0.0003**		**TNFα**	**0.576**	**0.005**
** **				** **		
**Spleen**	**IL-17A**	IL-17A		**Spleen**	**IL-17A**	
**GM-CSF**	0.231	0.267		**GM-CSF**	**-0.134**	0.552
**IL-10**	0.340	0.096		**IL-10**	**-0.564**	0.579
**TNFα**	0.325	0.113		**TNFα**	**-0.438**	**0.042**
** **				** **		
**Kidney**	**IL-17A**			**Kidney**	**IL-17A**	
**GM-CSF**	**0.571**	**0.003**		**GM-CSF**	**0.622**	**0.002**
**IL-10**	**0.410**	**0.042**		**IL-10**	**0.56**	**0.007**
**TNFα**	**0.860**	**3.702E-08**		**TNFα**	**0.558**	**0.007**

Red: Negative correlation coefficient (r) values. Bold: p < 0.05.

Finally, memory/effector Th17 cells were inferred in the liver, lung, gut, and kidney of WT mice and in the heart, gut, and kidney of TLR4^-/-^ mice (**Table 1**). Interestingly, we observed a negative correlation for IL-17A and TNF in the spleen of TLR4^-/-^ mice, suggesting that there was a reduction of these cells in that organ. We also observed nearly statistically significant Spearman correlations for memory/effector cells in the heart and systemic of WT mice, suggesting a reduction of this cell subset in these compartments.

Taken together, these results suggest that TLR4 impacts distinct populations of tissue-resident and/or tissue-infiltrating, IL-17A-producing cells following resuscitated hemorrhagic shock.

## Discussion

In the present study, we utilized a novel computational pipeline involving hypergraphs as a means of assessing the dynamic, trans-compartment evolution of inflammation subsequent to traumatic injury and resuscitated hemorrhagic shock. These studies extend prior work from our group in which we defined protein-level dynamic networks and principal drivers of inflammation in both experimental (18) and clinical (7, 12, 19–21, 41–46) settings of T/HS-R, as well as in endotoxemia in mice (14, 15).

The inflammatory response to trauma/hemorrhage involves an important role for TLR4 in transducing pro-inflammatory signals from DAMPs such as HMGB1 (17). This includes a variety of functions ranging from cytokine production and organ damage (47–52); intestinal damage/dysfunction and consequent bacterial translocation (52, 53); and coagulation abnormalities (54). These effects require TLR4 expression on both myeloid and dendritic cells (55), and this may explain the inferred role for the dendritic cell-derived cytokine IL-12 (56) in driving Th1 responses (IFN-γ) (56, 57) in our system. Our results also suggest that there are TLR4-dependent differences in resting/baseline inflammatory responses in various tissues, and that these differences are somewhat blurred following T/HS-R and then recover their distinct characteristics following sufficient time post-injury. We speculate that the baseline differences in tissue expression of inflammatory mediators may be related to the reported stable baseline difference in gut microbiome composition between these two mouse strains (58), since enterocyte TLR4 has been implicated in damage/dysfunction in other organs such as the lung (52). Given that T-HS/R induces a “genomic storm” (59) and extensive reprogramming of multiple aspects of physiology (11), it is perhaps not surprising that differences between WT and TLR4^-/-^ mice are blurred following injury.

Another key conclusion from our analyses is that TLR4 plays a complex role in regulating distinct inflammatory pathways in different organs/tissues, and this complexity and context dependence may have relevance for critical illness. Specifically, our experimental and computational studies suggest that there is a baseline difference in tissue distribution of multiple cytokines and chemokines between WT and TLR4^-/-^ mice; that surgical cannulation followed by hemorrhagic shock and resuscitation for 0.5-4h tends to reduce those differences; and that the baseline differences between WT and TLR4^-/-^ re-appear by 22h following resuscitation from hemorrhagic shock. Key organs impacted during this complex process appear to be the gut, kidney, liver, and lung; the plasma (systemic circulation) manifested less dramatic differences. The gut has long been implicated as a central driver of inflammatory responses and multiple organ dysfunction following trauma/hemorrhage (60).

### Impact of Hemorrhage, Resuscitation, and TLR4 on IL-17A-Related Pathways

Toll-like receptor 4-expressing cells may impact the response to T-HS/R *via* IL-17A, since IL-17A was present persistently at multiple time points across most tissues assessed. Indeed, HMGB1/TLR4-dependent pathways induced subsequent to T-HS/R are tightly intertwined with type 17 (or type 3) immunity. Early studies have suggested that T-HS/R induces neutrophil efflux from the bone marrow *via* IL-17A driven by HMGB1-mediated induction of IL-23 in a TLR4-dependent manner (61). This is likely a broadly conserved inflammatory axis that is not unique to trauma/hemorrhage, since IL-17A production in the context of tuberculosis (62), arthritis (63), and acetaminophen-induced liver inflammation (64). Notably, adenoviral transduction of IL-17A alone was sufficient to induce multi-tissue inflammation, and this required TLR4 (65). The inferred role for IL-12p40/p70 may also implicate Th17 responses (66). It is tempting to speculate that some of these intertwined effects of TLR4 and type 17 immunity ultimately manifest in the clinical outcomes of trauma patients (7, 12, 13).

The finding that IL-5 is present in spleen, lung, and gut of TLR4^-/-^ mice is intriguing in that it suggests a potential role for another IL-17A-producing cell type, namely innate lymphoid cells (ILCs) (67). We have recently reported on a novel pathway of inflammation following T/HS-R in WT mice in which IL-33 induces ILC2 activation in the lung, which in turn induces further IL-5 expression by CXCR2^+^ lung neutrophils and drives early lung injury (68). Our current results suggest the paradoxical possibility that TLR4 deficiency might exacerbate this lung injury; further studies are needed to test this hypothesis.

We also observed TLR4-dependent changes in the post-T/HS-R inflammation in the heart, kidney, liver, lung, TLR4^-/-^ mice exhibited elevated inflammation in the heart following resuscitation, while WT mice had little inflammation at baseline, post-hemorrhage, or following resuscitation in this organ. Renal, hepatic, pulmonary inflammatory responses were elevated at baseline in both mouse strains but diverged following hemorrhagic shock and inflammation. Notably, splenic inflammatory responses were overall low across all treatment groups, and we did not observe any important differences as a function of TLR4.

An important context for the work described herein is the definition of C57BL/6 as a “wild type” strain when comparing to TLR4^-/-^ mice. Notably, the C57BL/6 mouse strain exhibits Th1- (69) and M1- (70) dominant immune responses and has been utilized extensively in studies of experimental T/HS-R (71). Our prior studies suggest that the inflammatory over-responsiveness of this mouse strain – and especially IL-17A-related responses – may reflect the biology of trauma patients that exhibit overly robust, self-sustaining inflammation associated with sub-acute mortality (12) and other adverse clinical outcomes (7). As such, TLR4^-/-^ mice (or other mouse strains that are not explicitly Th1-dominant) may better reflect the responses of trauma survivors. Further comparative studies are needed to address this question.

### Novel Insights Into Spatiotemporal Dynamics of Inflammation From Hypergraph Analysis

Our prior work was aimed at defining networks of individual inflammatory mediators interacting over time *within* a given tissue, as well as principal characteristics/drivers of these responses. Here, we utilized hypergraphs to extend prior work defining how mediators interact *across* tissues over time (34). Hypergraphs provide several advantages when visualizing and interpreting multi-compartment data, such as that examined in this study. On a basic level, hypergraphs provide a method to visualize inflammatory mediators and their localization to specific tissue compartments across each time point. These visualizations can be simplified to elucidate differences between the localization of key inflammatory mediators at distinct time points between experimental conditions. In conjunction with computational and cross-correlation analyses, hypergraphs provide a spatial map upon which inference on how mediators are interacting within and between tissues may influence the spread of trans-compartmental inflammation. A key quantitative metric of hypergraphs, edge distribution, captures the network complexity of inflammation across tissue samples at a given time point. Changes in edge distribution can highlight important changes in inflammation such as the localization of a few inflammatory mediators to just one compartment or the dispersion of nearly all inflammatory mediators to all tissue compartments. In conjunction with PCA and cross-correlational analyses, hypergraphs provide the ultimate spatial framework upon which analyses regarding how and when inflammatory mediators interact with each to modulate inflammation.

## Limitations

There are several limitations of our study. These include the focus on a subset of inflammatory mediators that broadly interrogate inflammation and immunity but are not a comprehensive set [vs. recent studies that have assessed a broader array of molecules (11)], the absence of cell-level data (5, 72) to validate key hypotheses detailed above, and a lack of validation in human trauma/hemorrhage due to the difficulty in accessing the various tissues (other than the systemic circulation) analysed herein. Key among the inflammatory mediators that could not be assessed across tissue compartments is HMGB1, which is typically found within the nucleus but is secreted in settings of cellular stress and damage (73, 74), since this molecule would be released during the process of tissue homogenization. Another important, related limitation concerns the possibility that some of the phenomena we have described are due to the efflux of bacteria from the gut and PAMPs, rather than DAMPs. Studies in gnotobiotic mice (75) may be needed to address this question. Another important limitation concerns the lack of tissue-specific deletion of TLR4 as a means for directly testing some of the hypotheses raised by our analyses. Finally, it is important to note that all of the analyses presented in this study are based on statistical correlations, and correlation is not causality. We have shown that it is possible to obtain define potential biological mechanisms related to the response to trauma/hemorrhage *via* a process involving obtaining inflammatory mediator data, carrying out network inference, and then extracting core features into mechanistic computational models that can be interrogated under various conditions to validate and extend the conclusions that can be derived from purely data-driven analyses (76). Future studies will utilize these datasets to carry out this type of iterative, rational process.

## Conclusions

In conclusion, these studies demonstrate multiple novel findings while reinforcing prior conclusions regarding the complex role of TLR4 and type 17 immune responses following trauma/hemorrhage. These studies serve as the basis for future cell-level analyses aimed at yielding an integrated understanding of the spatiotemporal evolution of inflammatory networks in the context of injury and critical illness.

## Data Availability Statement

The original contributions presented in the study are included in the article/**Supplementary Material**. Further inquiries can be directed to the corresponding authors.

## Ethics Statement

The animal study was reviewed and approved by IACUC University of Pittsburgh.

## Author Contributions

AS: analysed data, wrote manuscript. RZ: analysed data, wrote manuscript. SK: obtained data, analysed data, edited manuscript. DB: obtained data. JY: obtained data. FE-D: analysed data. TB: conceived and directed study, analysed data, edited manuscript. YV: conceived and directed study, analysed data, wrote manuscript. All authors contributed to the article and approved the submitted version.

## Funding

AS: University of Pittsburgh School of Medicine Physician Scientist Training Program.

## Conflict of Interest

YV is a co-founder of, and stakeholder in, Immunetrics, Inc.

The remaining authors declare that the research was conducted in the absence of any commercial or financial relationships that could be construed as a potential conflict of interest.

## Publisher’s Note

All claims expressed in this article are solely those of the authors and do not necessarily represent those of their affiliated organizations, or those of the publisher, the editors and the reviewers. Any product that may be evaluated in this article, or claim that may be made by its manufacturer, is not guaranteed or endorsed by the publisher.

## References

[1] Peitzman AB Billiar TR Harbrecht BG Kelly E Udekwu AO Simmons RL. Hemorrhagic Shock. Curr Probl Surg (1995) 32(11):925–1002. doi: 10.1016/S0011-3840(05)80008-5 7587344

[2] de Munter L Polinder S Lansink KW Cnossen MC Steyerberg EW de Jongh MA. Mortality Prediction Models in the General Trauma Population: A Systematic Review. Injury (2017) 48(2):221–9. doi: 10.1016/j.injury.2016.12.009 28011072

[3] Lord JM Midwinter MJ Chen YF Belli A Brohi K Kovacs EJ. The Systemic Immune Response to Trauma: An Overview of Pathophysiology and Treatment. Lancet (2014) 384(9952):1455–65. doi: 10.1016/s0140-6736(14)60687-5 PMC4729362 25390327

[4] Namas R Mi Q Namas R Almahmoud K Zaaqoq A Abdul Malak O. Insights Into the Role of Chemokines, Damage-Associated Molecular Patterns, and Lymphocyte-Derived Mediators From Computational Models of Trauma-Induced Inflammation. Antiox Redox Signaling (2015) 10:1370–87. doi: 10.1089/ars.2015.6398 PMC4685502 26560096

[5] Chen T Delano MJ Chen K Sperry JL Namas RA Lamparello AJ. A Roadmap From Single-Cell Transcriptome to Patient Classification for the Immune Response to Trauma. JCI Insight (2020) 6(2):e145108. doi: 10.1172/jci.insight.145108 PMC7934885 33320841

[6] Billiar TR Vodovotz Y. Time for Trauma Immunology. PloS Med (2017) 14(7):e1002342. doi: 10.1371/journal.pmed.1002342 28700602 PMC5507395

[7] Schimunek L Lindberg H Cohen M Namas RA Mi Q Yin J. Computational Derivation of Core, Dynamic Human Blunt Trauma Inflammatory Endotypes. Front Immunol (2021) 11:589304(3481). doi: 10.3389/fimmu.2020.589304 33537029 PMC7848165

[8] Bonaroti J Abdelhamid S Kar U Sperry J Zamora R Namas RA. The Use of Multiplexing to Identify Cytokine and Chemokine Networks in the Immune-Inflammatory Response to Trauma. Antioxid Redox Signal (2021) 35:1393–406. doi: 10.1089/ars.2021.0054 PMC8905234 33860683

[9] Maier B Lefering R Lehnert M Laurer HL Steudel WI Neugebauer EA. Early Versus Late Onset of Multiple Organ Failure is Associated With Differing Patterns of Plasma Cytokine Biomarker Expression and Outcome After Severe Trauma. Shock (2007) 28(6):668–74. doi: 10.1097/shk.0b013e318123e64e 18092384

[10] Cyr A Zhong Y Reis SE Namas RA Amoscato A Zuckerbraun B. Analysis of the Plasma Metabolome After Trauma, Novel Circulating Sphingolipid Signatures, and in-Hospital Outcomes. J Am Coll Surg (2021) 232(3):276–87.e1. doi: 10.1016/j.jamcollsurg.2020.12.022 33453380 PMC11875205

[11] Wu J Vodovotz Y Abdelhamid S Guyette FX Yaffe MB Gruen DS. Multi-Omic Analysis in Injured Humans: Patterns Align With Outcomes and Treatment Responses. Cell Rep Med (2021) 2(12). doi: 10.1016/j.xcrm.2021.100478 PMC8715070 35028617

[12] Abboud AN Namas RA Ramadan M Mi Q Almahmoud K Abdul-Malak O. Computational Analysis Supports an Early, Type 17 Cell-Associated Divergence of Blunt Trauma Survival and Mortality. Crit Care Med (2016) 44:e1074–81. doi: 10.1097/CCM.0000000000001951 PMC5201164 27513538

[13] Seshadri A Brat GA Yorkgitis BK Keegan J Dolan J Salim A. Phenotyping the Immune Response to Trauma: A Multiparametric Systems Immunology Approach. Crit Care Med (2017) 45:1523–30. doi: 10.1097/ccm.0000000000002577 PMC10114604 28671900

[14] Zamora R Korff S Mi Q Barclay D Yin J Schimunek L. A Computational Analysis of Dynamic, Multi-Organ Inflammatory Crosstalk Induced by Endotoxin in Mice. PloS Comput Biol (2018) 6:e100658. doi: 10.1371/journal.pcbi.1006582 PMC6239343 30399158

[15] Zamora R Chavan S Zanos T Simmons RL Billiar TR Vodovotz Y. Spatiotemporally Specific Roles of TLR4, TNF, and IL-17A in Murine Endotoxin-Induced Inflammation Inferred From Analysis of Dynamic Networks. Mol Med (2021) 27(1):65. doi: 10.1186/s10020-021-00333-z 34167455 PMC8223370

[16] Beutler B. TLR4 as the Mammalian Endotoxin Sensor. Curr Top Microbiol Immunol (2002) 270:109–20. doi: 10.1007/978-3-642-59430-4_7 12467247

[17] Kaczorowski DJ Mollen KP Edmonds R Billiar TR. Early Events in the Recognition of Danger Signals After Tissue Injury. J Leukoc Biol (2008) 83(3):546–52. doi: 10.1189/jlb.0607374 18032691

[18] Mi Q Constantine G Ziraldo C Solovyev A Torres A Namas R. A Dynamic View of Trauma/Hemorrhage-Induced Inflammation in Mice: Principal Drivers and Networks. PLoS One (2011) 6(5):e19424. doi: 10.1371/journal.pone.0019424 21573002 PMC3091861

[19] Namas RA Vodovotz Y Almahmoud K Abdul-Malak O Zaaqoq A Namas R. Temporal Patterns of Circulating Inflammation Biomarker Networks Differentiate Susceptibility to Nosocomial Infection Following Blunt Trauma in Humans. Ann Surg (2016) 263:191–8. doi: 10.1097/sla.0000000000001001 PMC5136774 25371118

[20] Almahmoud K Namas RA Zaaqoq AM Abdul-Malak O Namas R Zamora R. Prehospital Hypotension is Associated With Altered Inflammation Dynamics and Worse Outcomes Following Blunt Trauma in Humans. Crit Care Med (2015) 43:1395–404. doi: 10.1097/ccm.0000000000000964 25803650

[21] Almahmoud K Namas RA Abdul-Malak O Zaaqoq AM Zamora R Zuckerbraun BS. Impact of Injury Severity on Dynamic Inflammation Networks Following Blunt Trauma. Shock (2015) 44:105–9. doi: 10.1097/shk.0000000000000395 PMC4504837 26009819

[22] Namas R Almahmoud K Mi Q Ghuma A Namas R Zaaqoq A. Individual-Specific Principal Component Analysis of Circulating Inflammatory Mediators Predicts Early Organ Dysfunction in Trauma Patients. J Crit Care (2016) 36:146–53. doi: 10.1016/j.jcrc.2016.07.002 PMC5097026 27546764

[23] Tian Z Hwang T Kuang R. A Hypergraph-Based Learning Algorithm for Classifying Gene Expression and arrayCGH Data With Prior Knowledge. Bioinformatics (2009) 25(21):2831–8. doi: 10.1093/bioinformatics/btp467 19648139

[24] Bernal A Daza E. Metabolic Networks: Beyond the Graph. Curr Comput Aided Drug Des (2011) 7(2):122–32. doi: 10.2174/157340911795677611 21539508

[25] Lugo-Martinez J Zeiberg D Gaudelet T Malod-Dognin N Pržulj N Radivojac P. Classification in Biological Networks With Hypergraphlet Kernels. Bioinformatics (2020) 37(7):1000–7. doi: 10.1093/bioinformatics/btaa768 PMC8128478 32886115

[26] Gao Y Zhang Z Lin H Zhao X Du S Zou C. Hypergraph Learning: Methods and Practices. IEEE Trans Pattern Anal Mach Intell (2020) 44(5):2548–66. doi: 10.1109/tpami.2020.3039374 33211654

[27] Deng M Scott MJ Loughran P Gibson G Sodhi C Watkins S. Lipopolysaccharide Clearance, Bacterial Clearance, and Systemic Inflammatory Responses are Regulated by Cell Type-Specific Functions of TLR4 During Sepsis. J Immunol (2013) 190(10):5152–60. doi: 10.4049/jimmunol.1300496 PMC3644895 23562812

[28] Pfeifer R Kobbe P Darwiche SS Billiar TR Pape HC. Role of Hemorrhage in the Induction of Systemic Inflammation and Remote Organ Damage: Analysis of Combined Pseudo-Fracture and Hemorrhagic Shock. J Orthop Res (2011) 29(2):270–4. doi: 10.1002/jor.21214 20690183

[29] Barclay D Zamora R Torres A Namas R Steed D Vodovotz Y. A Simple, Rapid, and Convenient Luminex™-Compatible Method of Tissue Isolation. J Clin Lab Anal (2008) 22:278–81. doi: 10.1002/jcla.20253 PMC3640326 18623112

[30] Metukuri MR Beer-Stolz D Namas RA Dhupar R Torres A Loughran PA. Expression and Subcellular Localization of BNIP3 in Hypoxic Hepatocytes and Liver Stress. Am J Physiol Gastrointest Liver Physiol (2009) 296(3):G499–509. doi: 10.1152/ajpgi.90526.2008 PMC2660177 19147804

[31] Chong J Wishart DS Xia J. Using MetaboAnalyst 4.0 for Comprehensive and Integrative Metabolomics Data Analysis. Curr Protoc Bioinf (2019) 68(1):e86. doi: 10.1002/cpbi.86 31756036

[32] Chong J Soufan O Li C Caraus I Li S Bourque G. MetaboAnalyst 4.0: Towards More Transparent and Integrative Metabolomics Analysis. Nucleic Acids Res (2018) 46(W1):W486–94. doi: 10.1093/nar/gky310 PMC6030889 29762782

[33] Feng S Heath E Jefferson B Joslyn C Kvinge H Mitchell HD. Hypergraph Models of Biological Networks to Identify Genes Critical to Pathogenic Viral Response. BMC Bioinf (2021) 22(1):287. doi: 10.1186/s12859-021-04197-2 PMC8164482 34051754

[34] Aral AM Zamora R Barclay D Yin J El-Dehaibi F Erbas VE. The Effects of Tacrolimus on Tissue-Specific, Protein-Level Inflammatory Networks in Vascularized Composite Allotransplantation. Front Immunol (2021) 12:591154(1529). doi: 10.3389/fimmu.2021.591154 34017323 PMC8129572

[35] Weaver CT Hatton RD Mangan PR Harrington LE. IL-17 Family Cytokines and the Expanding Diversity of Effector T Cell Lineages. Annu Rev Immunol (2007) 25:821–52. doi: 10.1146/annurev.immunol.25.022106.141557 17201677

[36] Korn T Bettelli E Oukka M Kuchroo VK. IL-17 and Th17 Cells. Annu Rev Immunol (2009) 27:485–517. doi: 10.1146/annurev.immunol.021908.132710 19132915

[37] Cua DJ Tato CM. Innate IL-17-Producing Cells: The Sentinels of the Immune System. Nat Rev Immunol (2010) 10(7):479–89. doi: 10.1038/nri2800 20559326

[38] Peters A Lee Y Kuchroo VK. The Many Faces of Th17 Cells. Curr Opin Immunol (2011) 23(6):702–6. doi: 10.1016/j.coi.2011.08.007 PMC3232281 21899997

[39] Langrish CL Chen Y Blumenschein WM Mattson J Basham B Sedgwick JD. IL-23 Drives a Pathogenic T Cell Population That Induces Autoimmune Inflammation. J Exp Med (2005) 201(2):233–40. doi: 10.1084/jem.20041257 PMC2212798 15657292

[40] McArthur MA Sztein MB. Unexpected Heterogeneity of Multifunctional T Cells in Response to Superantigen Stimulation in Humans. Clin Immunol (2013) 146(2):140–52. doi: 10.1016/j.clim.2012.12.003 PMC3565224 23333555

[41] Zaaqoq AM Namas R Almahmoud K Azhar N Mi Q Zamora R. Inducible Protein-10, a Potential Driver of Neurally-Controlled IL-10 and Morbidity in Human Blunt Trauma. Crit Care Med (2014) 42:1487–97. doi: 10.1097/CCM.0000000000000248 PMC4133186 24584064

[42] Brown D Namas RA Almahmoud K Zaaqoq A Sarkar J Barclay DA. Trauma *in Silico*: Individual-Specific Mathematical Models and Virtual Clinical Populations. Sci Transl Med (2015) 7:285ra61. doi: 10.1126/scitranslmed.aaa3636 25925680

[43] Abdul-Malak O Vodovotz Y Zaaqoq A Guardado J Almahmoud K Yin J. Elevated Admission Base Deficit is Associated With a Complex Dynamic Network of Systemic Inflammation Which Drives Clinical Trajectories in Blunt Trauma Patients. Mediators Inflamm (2016) 2016:7950374. doi: 10.1155/2016/7950374 27974867 PMC5126463

[44] Lamparello AJ Namas RA Abdul-Malak O Vodovotz Y Billiar TR. Young and Aged Blunt Trauma Patients Display Major Differences in Circulating Inflammatory Mediator Profiles After Severe Injury. J Am Coll Surg (2019) 228:148–60.e7. doi: 10.1016/j.jamcollsurg.2018.10.019 30448299 PMC7768550

[45] Almahmoud K Abboud A Namas RA Zamora R Sperry J Peitzman AB. Computational Evidence for an Early, Amplified Systemic Inflammation Program in Polytrauma Patients With Severe Extremity Injuries. PloS One (2019) 14(6):e0217577. doi: 10.1371/journal.pone.0217577 31163056 PMC6548366

[46] Zaaqoq AM Namas RA Abdul-Malak O Almahmoud K Barclay D Yin J. Diurnal Variation in Systemic Acute Inflammation and Clinical Outcomes Following Severe Blunt Trauma. Front Immunol (2019) 10:2699(2699). doi: 10.3389/fimmu.2019.02699 31824494 PMC6879654

[47] Meng X Ao L Song Y Raeburn CD Fullerton DA Harken AH. Signaling for Myocardial Depression in Hemorrhagic Shock: Roles of Toll-Like Receptor 4 and P55 TNF-Alpha Receptor. Am J Physiol Regul Integr Comp Physiol (2005) 288(3):R600–6. doi: 10.1152/ajpregu.00182.2004 15514106

[48] Prince JM Levy RM Yang R Mollen KP Fink MP Vodovotz Y. Toll-Like Receptor-4 Signaling Mediates Hepatic Injury and Systemic Inflammation in Hemorrhagic Shock. J Am Coll Surg (2006) 202(3):407–17. doi: 10.1016/j.jamcollsurg.2005.11.021 16500244

[49] Lv T Shen X Shi Y Song Y. TLR4 is Essential in Acute Lung Injury Induced by Unresuscitated Hemorrhagic Shock. J Trauma (2009) 66(1):124–31. doi: 10.1097/TA.0b013e318181e555 19131815

[50] Reino DC Pisarenko V Palange D Doucet D Bonitz RP Lu Q. Trauma Hemorrhagic Shock-Induced Lung Injury Involves a Gut-Lymph-Induced TLR4 Pathway in Mice. PloS One (2011) 6(8):e14829. doi: 10.1371/journal.pone.0014829 21829592 PMC3150139

[51] Benhamou Y Favre J Musette P Renet S Thuillez C Richard V. Toll-Like Receptors 4 Contribute to Endothelial Injury and Inflammation in Hemorrhagic Shock in Mice. Crit Care Med (2009) 37(5):1724–8. doi: 10.1097/CCM.0b013e31819da805 19325486

[52] Sodhi CP Jia H Yamaguchi Y Lu P Good M Egan C. Intestinal Epithelial TLR-4 Activation is Required for the Development of Acute Lung Injury After Trauma/Hemorrhagic Shock *via* the Release of HMGB1 From the Gut. J Immunol (2015) 194(10):4931–9. doi: 10.4049/jimmunol.1402490 PMC4417407 25862813

[53] Neal MD Leaphart C Levy R Prince J Billiar TR Watkins S. Enterocyte TLR4 Mediates Phagocytosis and Translocation of Bacteria Across the Intestinal Barrier. J Immunol (2006) 176(5):3070–9. doi: 10.4049/jimmunol.176.5.3070 16493066

[54] Ding N Chen G Hoffman R Loughran PA Sodhi CP Hackam DJ. Toll-Like Receptor 4 Regulates Platelet Function and Contributes to Coagulation Abnormality and Organ Injury in Hemorrhagic Shock and Resuscitation. Circ Cardiovasc Genet (2014) 7(5):615–24. doi: 10.1161/circgenetics.113.000398 PMC4270899 25049041

[55] Zettel K Korff S Zamora R Morelli AE Darwiche S Loughran PA. Toll-Like Receptor 4 on Both Myeloid Cells and Dendritic Cells is Required for Systemic Inflammation and Organ Damage After Hemorrhagic Shock With Tissue Trauma in Mice. Front Immunol (2017) 8:1672(1672). doi: 10.3389/fimmu.2017.01672 29234326 PMC5712321

[56] Trinchieri G. Interleukin-12 and the Regulation of Innate Resistance and Adaptive Immunity. Nat Rev Immunol (2003) 3(2):133–46. doi: 10.1038/nri1001 12563297

[57] Lyakh L Trinchieri G Provezza L Carra G Gerosa F. Regulation of Interleukin-12/Interleukin-23 Production and the T-Helper 17 Response in Humans. Immunol Rev (2008) 226:112–31. doi: 10.1111/j.1600-065X.2008.00700.x PMC2766610 19161420

[58] Ubeda C Lipuma L Gobourne A Viale A Leiner I Equinda M. Familial Transmission Rather Than Defective Innate Immunity Shapes the Distinct Intestinal Microbiota of TLR-Deficient Mice. J Exp Med (2012) 209(8):1445–56. doi: 10.1084/jem.20120504 PMC3409501 22826298

[59] Xiao W Mindrinos MN Seok J Cuschieri J Cuenca AG Gao H. A Genomic Storm in Critically Injured Humans. J Exp Med (2011) 208(13):2581–90. doi: 10.1084/jem.20111354 PMC3244029 22110166

[60] Deitch EA. Role of the Gut Lymphatic System in Multiple Organ Failure. Curr Opin Crit Care (2001) 7(2):92–8. doi: 10.1097/00075198-200104000-00007 11373517

[61] Liu Y Yuan Y Li Y Zhang J Xiao G Vodovotz Y. Interacting Neuroendocrine and Innate and Acquired Immune Pathways Regulate Neutrophil Mobilization From Bone Marrow Following Hemorrhagic Shock. J Immunol (2009) 182(1):572–80. doi: 10.4049/jimmunol.182.1.572 PMC2610356 19109190

[62] van de Veerdonk FL Teirlinck AC Kleinnijenhuis J Kullberg BJ van CR van der Meer JW. Mycobacterium Tuberculosis Induces IL-17A Responses Through TLR4 and Dectin-1 and is Critically Dependent on Endogenous IL-1. J Leukoc Biol (2010) 88(2):227–32. doi: 10.1189/jlb.0809550 20299682

[63] Pierer M Wagner U Rossol M Ibrahim S. Toll-Like Receptor 4 is Involved in Inflammatory and Joint Destructive Pathways in Collagen-Induced Arthritis in DBA1J Mice. PloS One (2011) 6(8):e23539. doi: 10.1371/journal.pone.0023539 21858160 PMC3157404

[64] Wang X Sun R Wei H Tian Z. High-Mobility Group Box 1 (HMGB1)-Toll-Like Receptor (TLR)4-Interleukin (IL)-23-IL-17A Axis in Drug-Induced Damage-Associated Lethal Hepatitis: Interaction of Gammadelta T Cells With Macrophages. Hepatology (2013) 57(1):373–84. doi: 10.1002/hep.25982 22821628

[65] Tang H Pang S Wang M Xiao X Rong Y Wang H. TLR4 Activation is Required for IL-17-Induced Multiple Tissue Inflammation and Wasting in Mice. J Immunol (2010) 185(4):2563–9. doi: 10.4049/jimmunol.0903664 20631308

[66] Patel DD Kuchroo VK. Th17 Cell Pathway in Human Immunity: Lessons From Genetics and Therapeutic Interventions. Immunity (2015) 43(6):1040–51. doi: 10.1016/j.immuni.2015.12.003 26682981

[67] Ebbo M Crinier A Vély F Vivier E. Innate Lymphoid Cells: Major Players in Inflammatory Diseases. Nat Rev Immunol (2017) 17(11):665–78. doi: 10.1038/nri.2017.86 28804130

[68] Xu J Guardado J Hoffman R Xu H Namas R Vodovotz Y. IL-33 Drives Lung Injury *via* ILC2 Promotion of Neutrophil IL-5 Production After Polytrauma. PloS Med (2017) 14:e1002365. 28742815 10.1371/journal.pmed.1002365 PMC5526517

[69] Mosmann TR Coffman RL. TH1 and TH2 Cells: Different Patterns of Lymphokine Secretion Lead to Different Functional Properties. Annu Rev Immunol (1989) 7:145–73. doi: 10.1146/annurev.iy.07.040189.001045 2523712

[70] Watanabe H Numata K Ito T Takagi K Matsukawa A. Innate Immune Response in Th1- and Th2-Dominant Mouse Strains. Shock (2004) 22(5):460–6. doi: 10.1097/01.shk.0000142249.08135.e9 15489639

[71] Mira JC Nacionales DC Loftus TJ Ungaro R Mathias B Mohr AM. Mouse Injury Model of Polytrauma and Shock. Methods Mol Biol (2018) 1717:1–15. doi: 10.1007/978-1-4939-7526-6_1 29468579 PMC6296232

[72] Chen T Conroy J Wang X Situ M Namas RA Vodovotz Y. The Independent Prognostic Value of Global Epigenetic Alterations: An Analysis of Single-Cell ATAC-Seq of Circulating Leukocytes From Trauma Patients Followed by Validation in Whole Blood Leukocyte Transcriptomes Across Three Etiologies of Critical Illness. eBioMedicine (2022) 76:103860. doi: 10.1016/j.ebiom.2022.103860 35124428 PMC8822299

[73] Czura CJ Wang H Tracey KJ. Dual Roles for HMGB1: DNA Binding and Cytokine. J Endotoxin Res (2001) 7(4):315–21. doi: 10.1177/09680519010070041401 11717586

[74] Wang H Yang H Tracey KJ. Extracellular Role of HMGB1 in Inflammation and Sepsis. J Intern Med (2004) 255(3):320–31. doi: 10.1111/j.1365-2796.2003.01302.x 14871456

[75] Yang R Gallo DJ Baust JJ Watkins SK Delude RL Fink MP. Effect of Hemorrhagic Shock on Gut Barrier Function and Expression of Stress-Related Genes in Normal and Gnotobiotic Mice. Am J Physiol Regul Integr Comp Physiol (2002) 283(5):R1263–74. doi: 10.1152/ajpregu.00278.2002 12376421

[76] Azhar N Namas RA Almahmoud K Zaaqoq A Malak OA Barclay D. A Putative “Chemokine Switch” That Regulates Systemic Acute Inflammation in Humans. Sci Rep (2021) 11(1):9703. doi: 10.1038/s41598-021-88936-8 33958628 PMC8102583

