# Supplementary material for: Inferring Tissue-Specific, TLR4-Dependent Type 17 Immune Interactions in Experimental Trauma/Hemorrhagic Shock and Resuscitation Using Computational Modeling
Source: Front Immunol. 2022 May 19;13:908618. doi: 10.3389/fimmu.2022.908618 (PMC9160183; doi:10.3389/fimmu.2022.908618)
Supplement: Supplementary file 1 [file DataSheet_1.pdf]

Supplementary Material

Supplementary Figures and Tables

Suppl. Fig. 1. Time-dependent release of inflammatory mediators in mice undergoing HS ± R (pseudo-fracture/hemorrhagic shock ± resuscitation). Wild type (WT) and TLR4$^{-/-}$ mice were randomly assigned to one of three experimental groups: Control (Ctrl, animals sacrificed directly after anesthesia to obtain physiological baseline levels, n=4-5), HS (animals subjected to pseudo-fracture followed by pressure controlled hemorrhagic shock, n=4), and HS/R (animals subjected to pseudo-fracture and hemorrhagic shock followed by 30 min, 1h, 4h, and 22h resuscitation, n=4 each), as described in *Materials and Methods*. Plasma and tissue samples were collected and assayed for inflammatory mediators suing multiplex Luminex™ assay as described in *Materials and Methods* Cytokine concentrations are expressed in pg/mg protein (tissue) or pg/ml (plasma) + SEM as indicated (WT vs. TLR4$^{-/-}$, analyzed by Two-Way ANOVA, significance set at $P<0.05$).

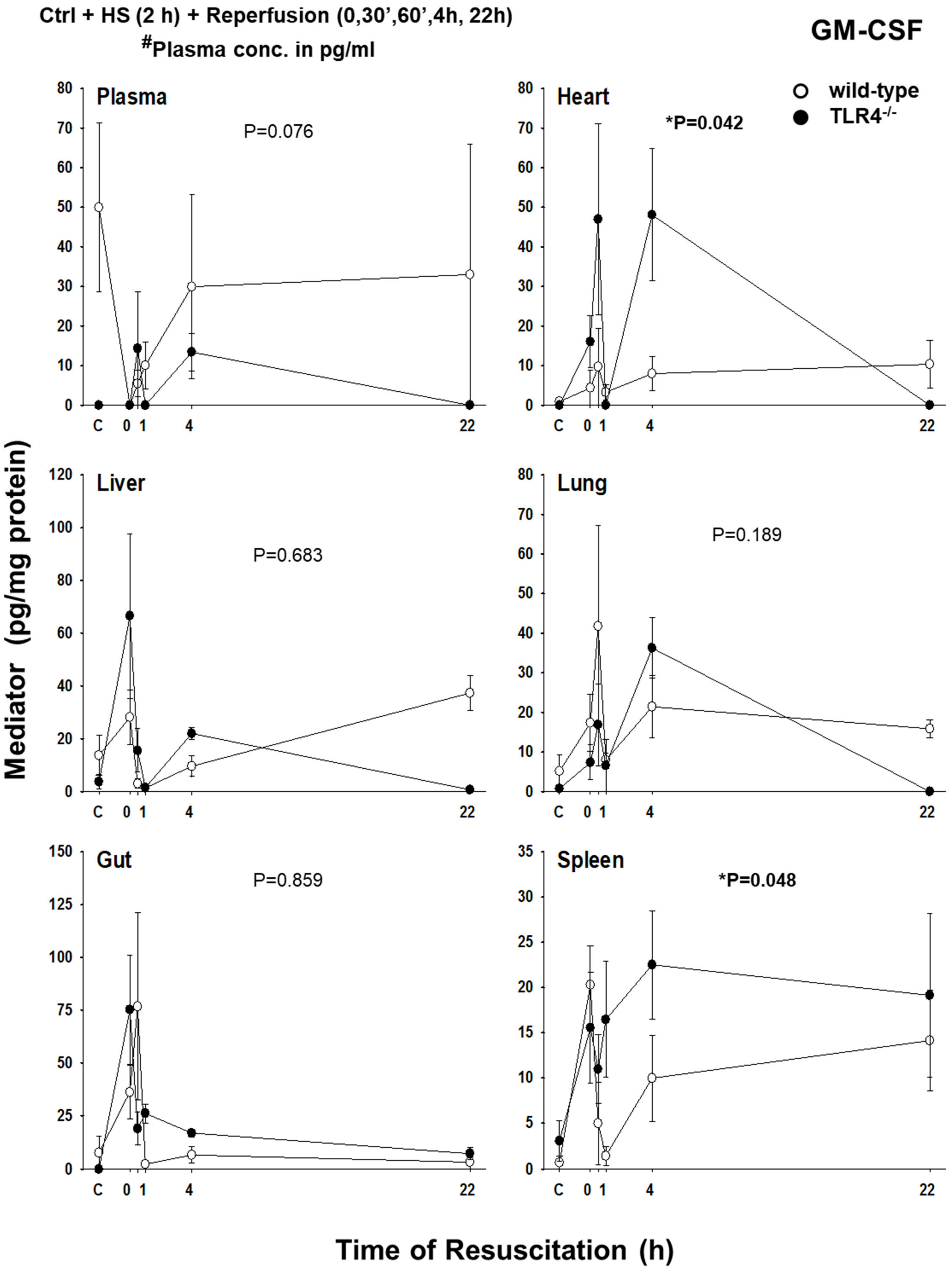
Ctrl + HS (2 h) + Reperfusion (0,30',60',4h, 22h)
#Plasma conc. in pg/ml
GM-CSF
wild-type
TLR4-/-
Plasma
P=0.076
Heart
*P=0.042
Liver
P=0.683
Lung
P=0.189
Gut
P=0.859
Spleen
*P=0.048
Mediator (pg/mg protein)
Time of Resuscitation (h)
C
0
1
4
22

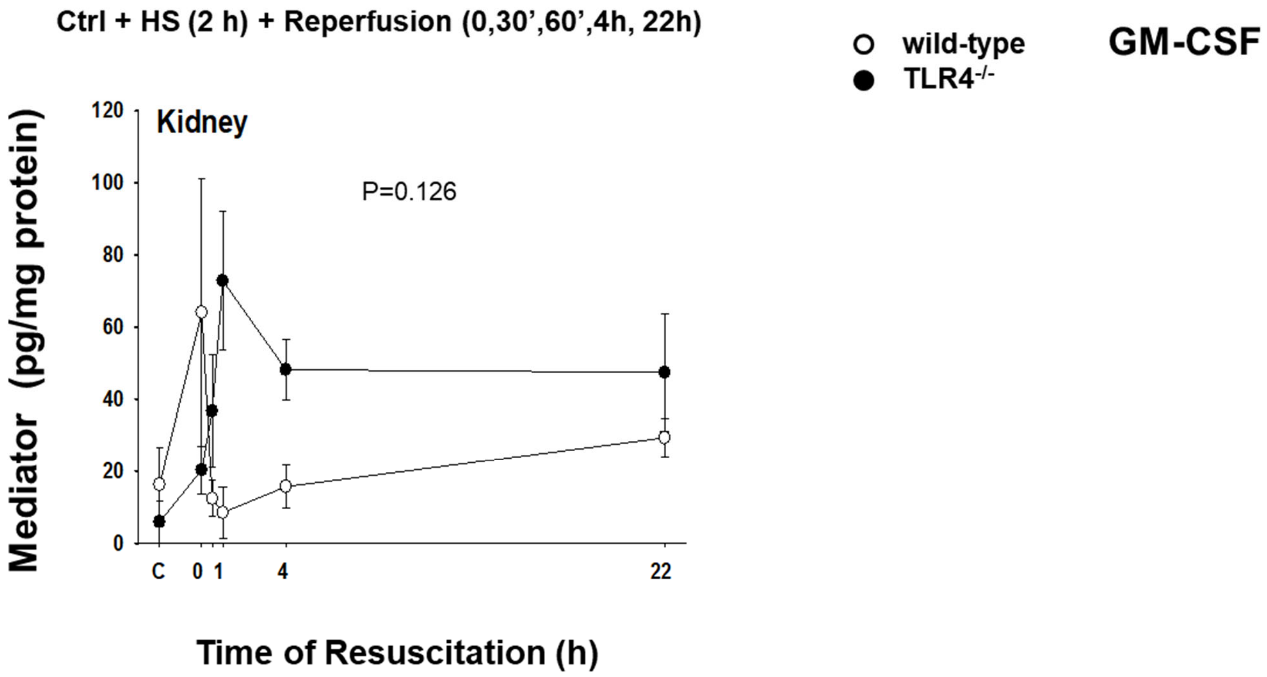
Ctrl + HS (2 h) + Reperfusion (0,30',60',4h, 22h)
wild-type
TLR4-/-
GM-CSF
Kidney
P=0.126
Mediator (pg/mg protein)
120
100
80
60
40
20
0
C
0
1
4
22
Time of Resuscitation (h)

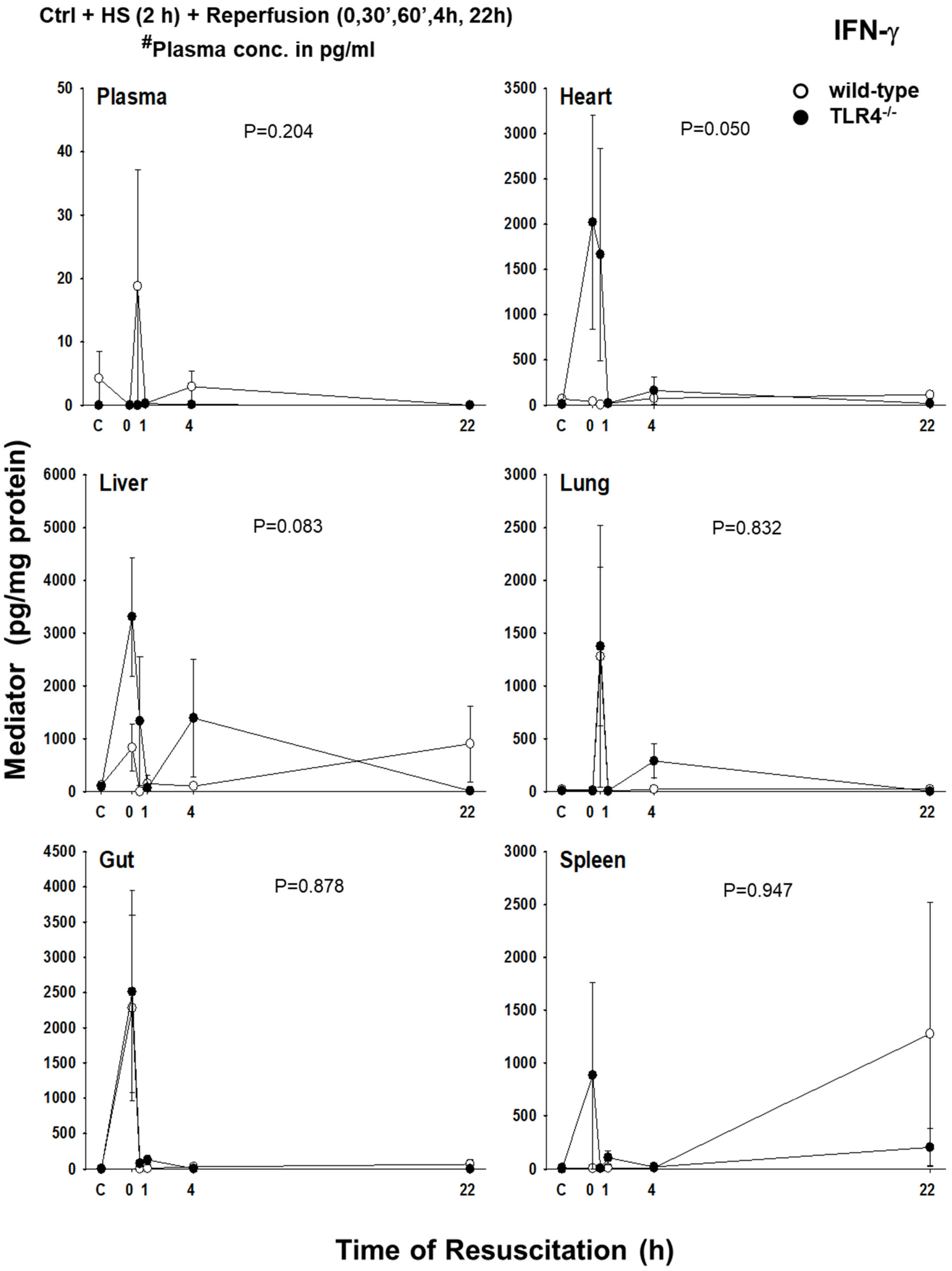
Ctrl + HS (2 h) + Reperfusion (0,30',60',4h, 22h)
#Plasma conc. in pg/ml
IFN-γ
wild-type
TLR4-/-
Plasma
P=0.204
Heart
P=0.050
Liver
P=0.083
Lung
P=0.832
Gut
P=0.878
Spleen
P=0.947
Mediator (pg/mg protein)
Time of Resuscitation (h)
C
0
1
4
22

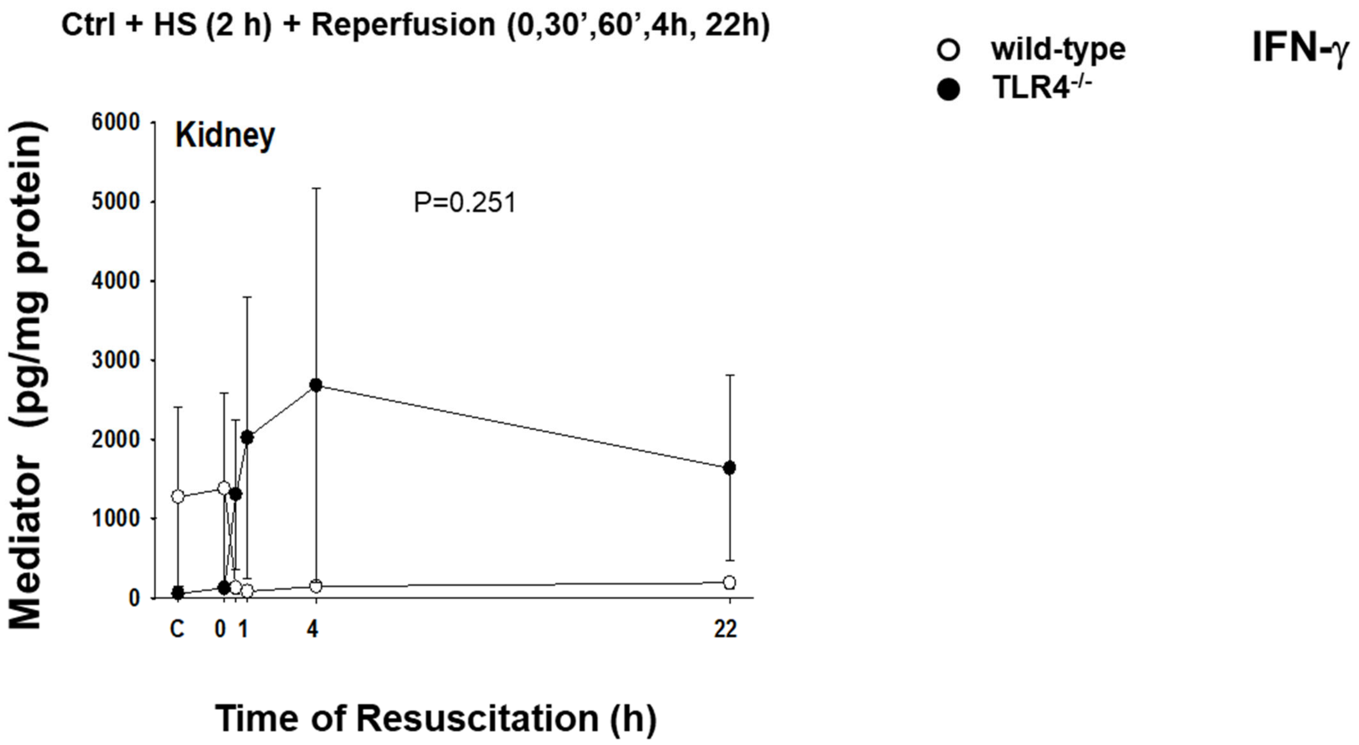
Ctrl + HS (2 h) + Reperfusion (0,30',60',4h, 22h)
wild-type
TLR4-/-
IFN-γ
Kidney
P=0.251
Mediator (pg/mg protein)
0
1000
2000
3000
4000
5000
6000
C
0
1
4
22
Time of Resuscitation (h)

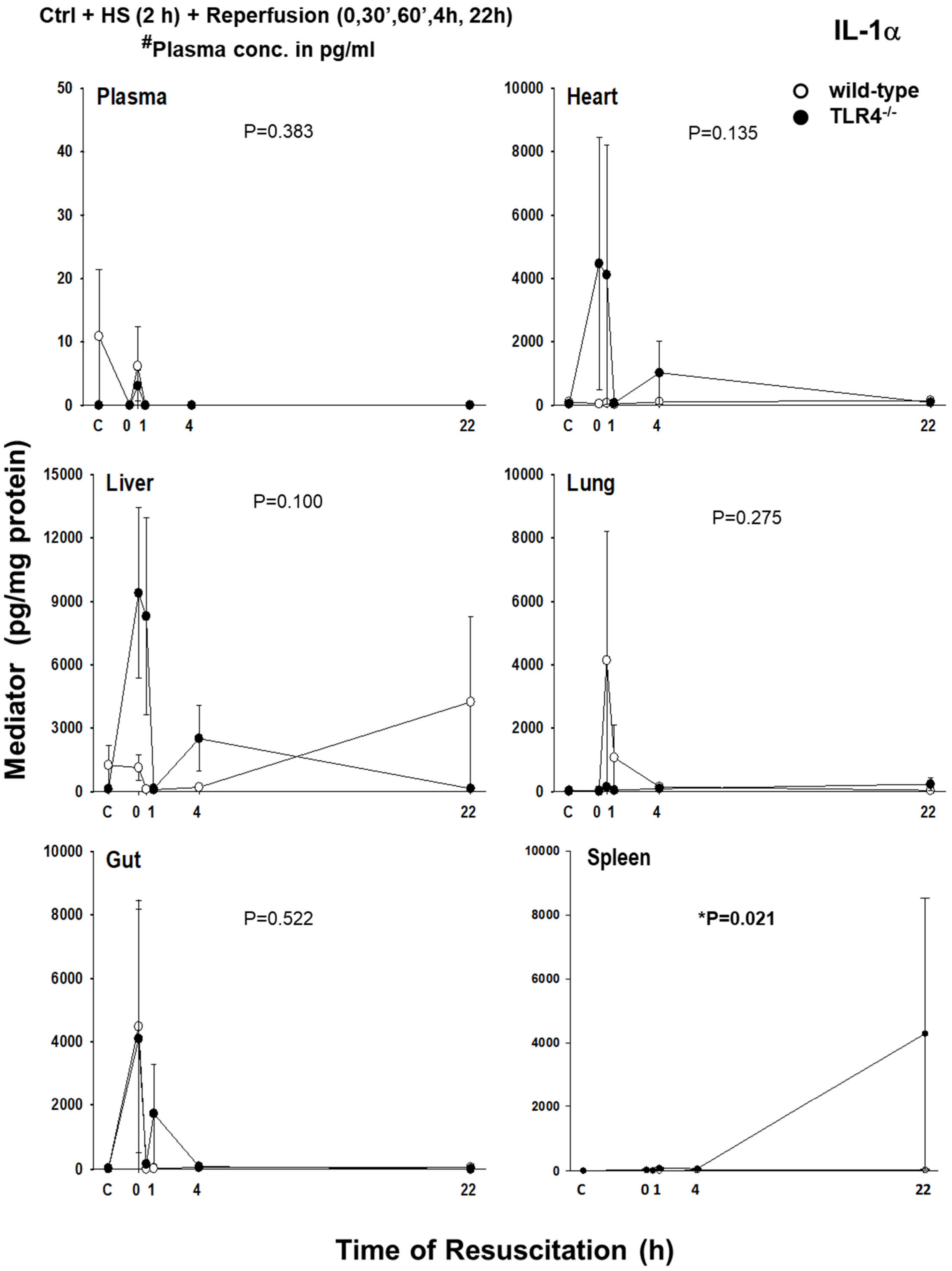

Ctrl + HS (2 h) + Reperfusion (0,30',60',4h, 22h)
#Plasma conc. in pg/ml
IL-1α
wild-type
TLR4-/-
Plasma
P=0.383
Heart
P=0.135
Liver
P=0.100
Lung
P=0.275
Gut
P=0.522
Spleen
*P=0.021
Mediator (pg/mg protein)
Time of Resuscitation (h)
C
0
1
4
22

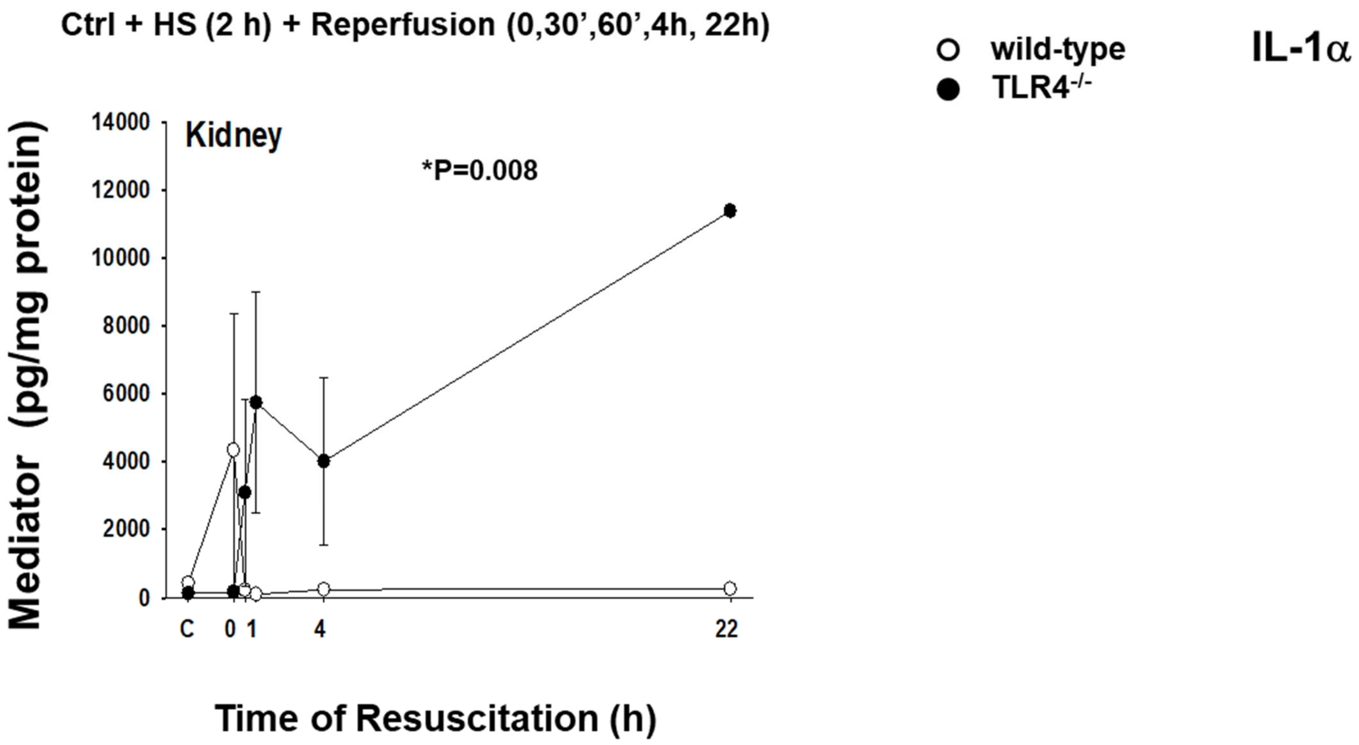
Ctrl + HS (2 h) + Reperfusion (0,30’,60’,4h, 22h)
wild-type
TLR4-/-
IL-1α
Kidney
*P=0.008
Mediator (pg/mg protein)
14000
12000
10000
8000
6000
4000
2000
0
C
0
1
4
22
Time of Resuscitation (h)

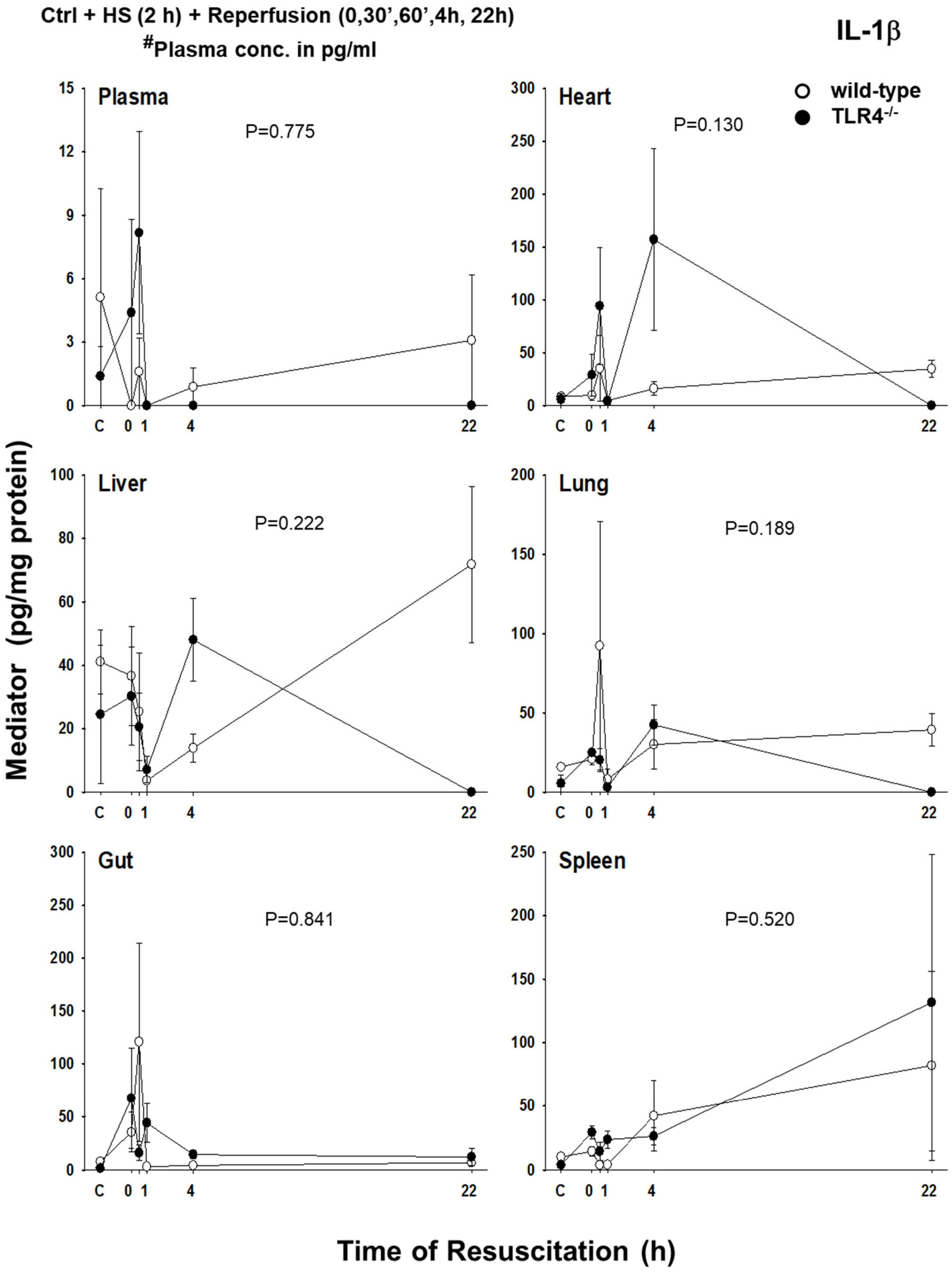

Ctrl + HS (2 h) + Reperfusion (0,30',60',4h, 22h)
#Plasma conc. in pg/ml
IL-1β
wild-type
TLR4-/-
Plasma
P=0.775
Heart
P=0.130
Liver
P=0.222
Lung
P=0.189
Gut
P=0.841
Spleen
P=0.520
Mediator (pg/mg protein)
Time of Resuscitation (h)
C
0
1
4
22

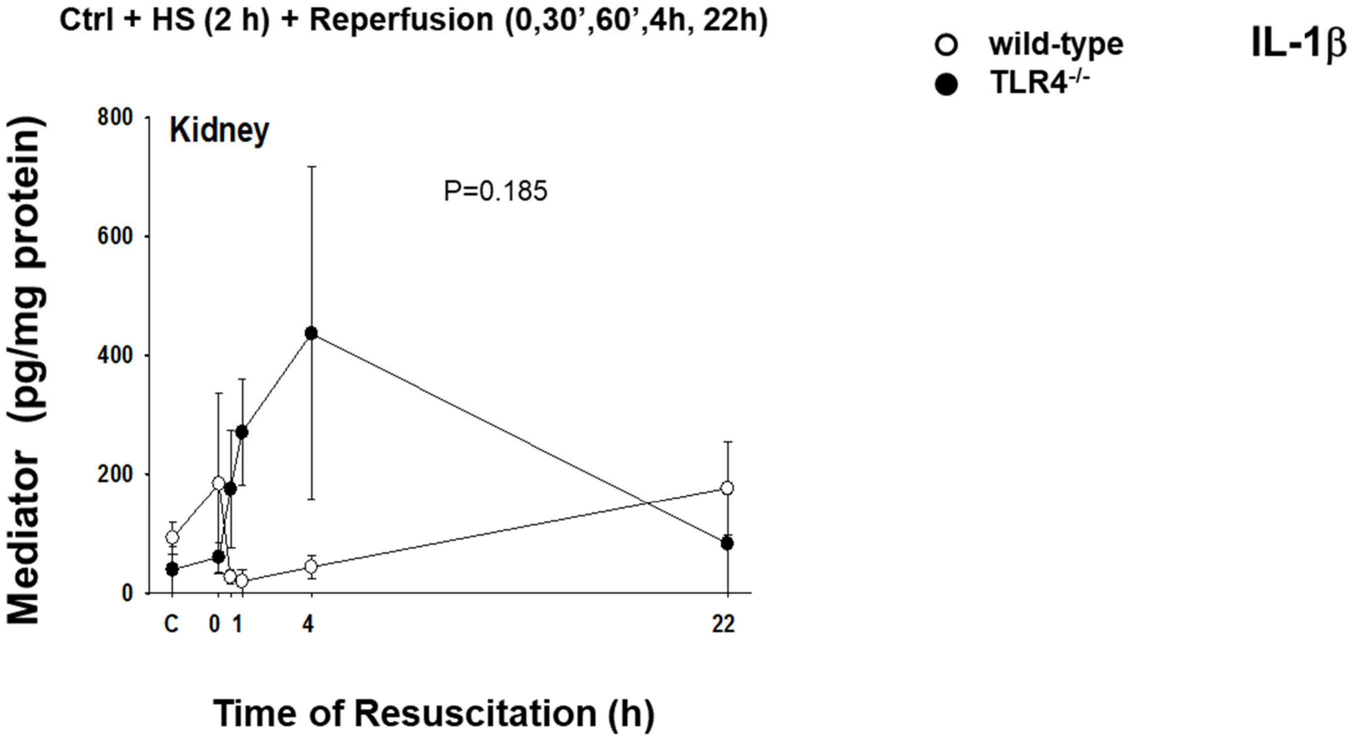
Ctrl + HS (2 h) + Reperfusion (0,30',60',4h, 22h)
wild-type
TLR4-/-
IL-1β
Kidney
P=0.185
Mediator (pg/mg protein)
800
600
400
200
0
C
0
1
4
22
Time of Resuscitation (h)

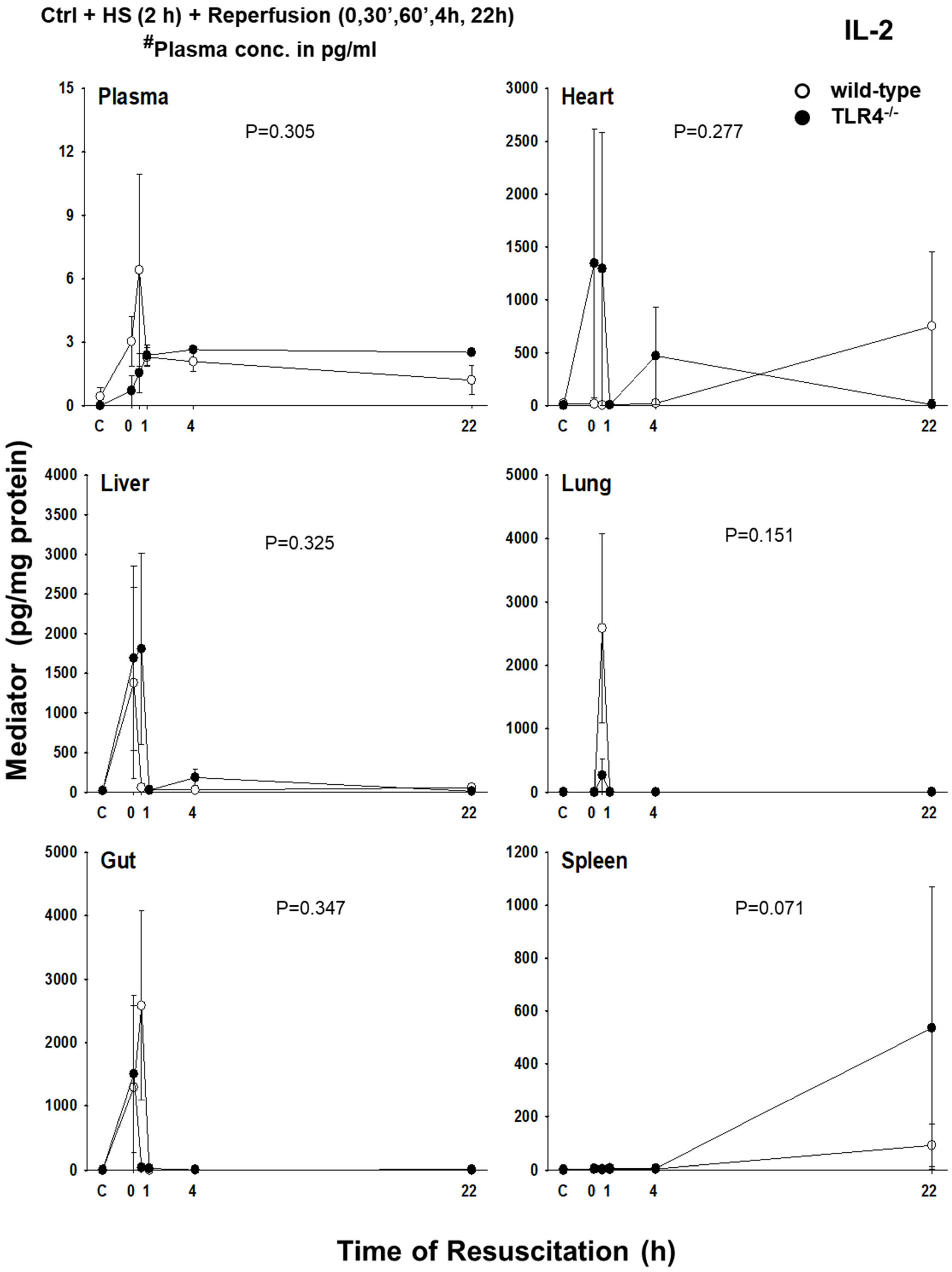
Ctrl + HS (2 h) + Reperfusion (0,30',60',4h, 22h)
#Plasma conc. in pg/ml
IL-2
wild-type
TLR4-/-
Plasma
P=0.305
Heart
P=0.277
Liver
P=0.325
Lung
P=0.151
Gut
P=0.347
Spleen
P=0.071
Mediator (pg/mg protein)
Time of Resuscitation (h)
C
0
1
4
22

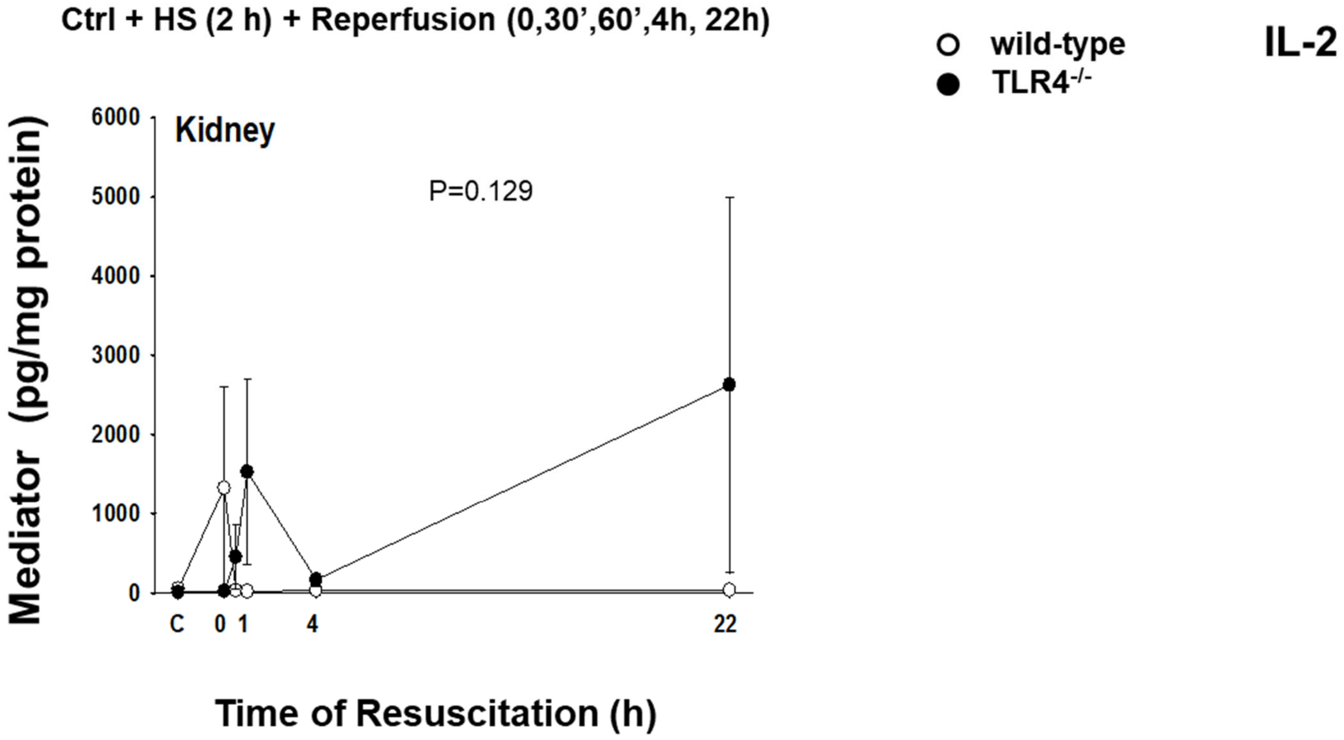
Ctrl + HS (2 h) + Reperfusion (0,30’,60’,4h, 22h)
wild-type
TLR4-/-
IL-2
Kidney
P=0.129
Mediator (pg/mg protein)
6000
5000
4000
3000
2000
1000
0
C
0
1
4
22
Time of Resuscitation (h)

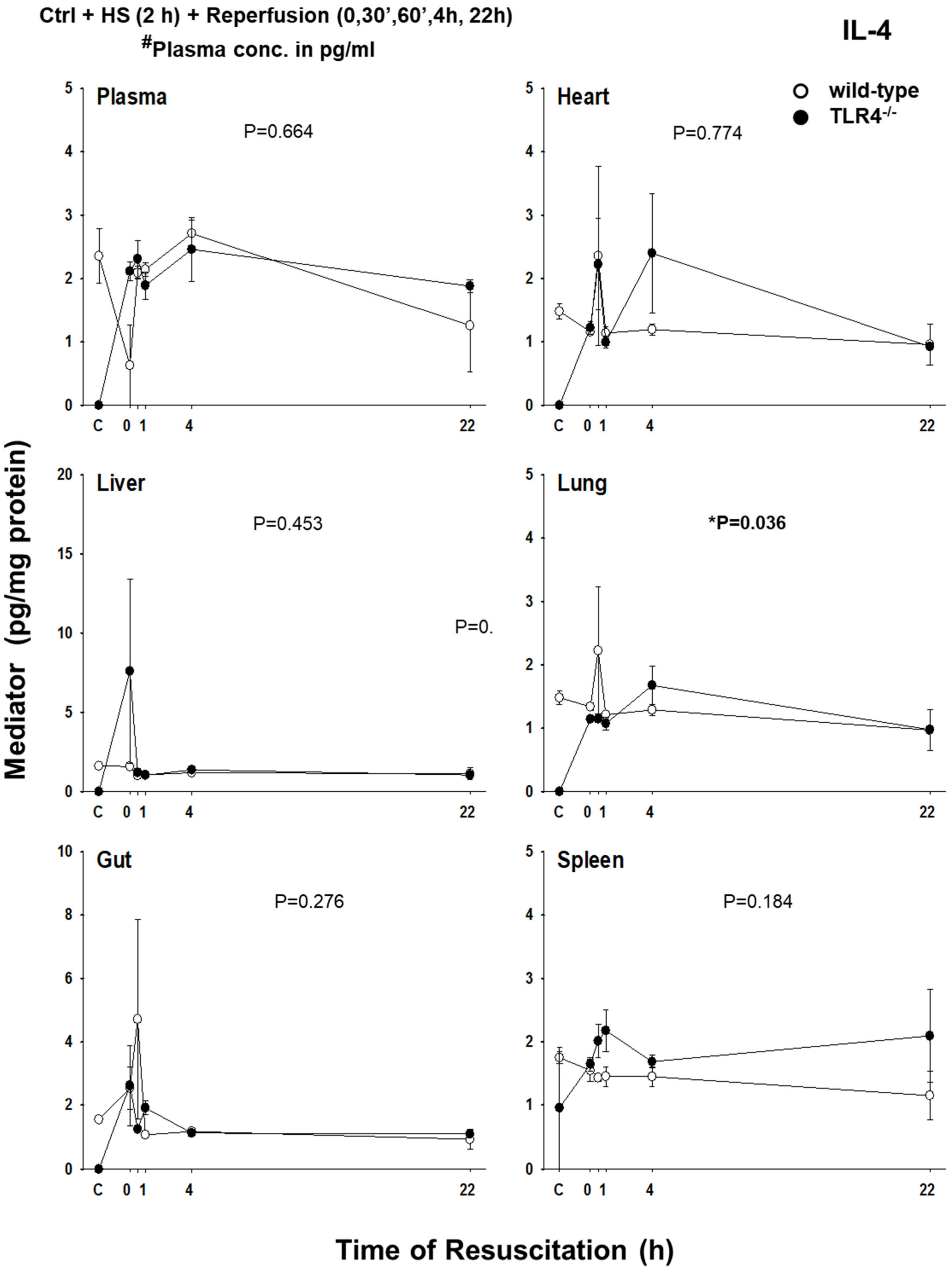
Ctrl + HS (2 h) + Reperfusion (0,30',60',4h, 22h)
#Plasma conc. in pg/ml
IL-4
wild-type
TLR4-/-
Plasma
P=0.664
Heart
P=0.774
Liver
P=0.453
P=0.
Lung
*P=0.036
Gut
P=0.276
Spleen
P=0.184
Mediator (pg/mg protein)
Time of Resuscitation (h)

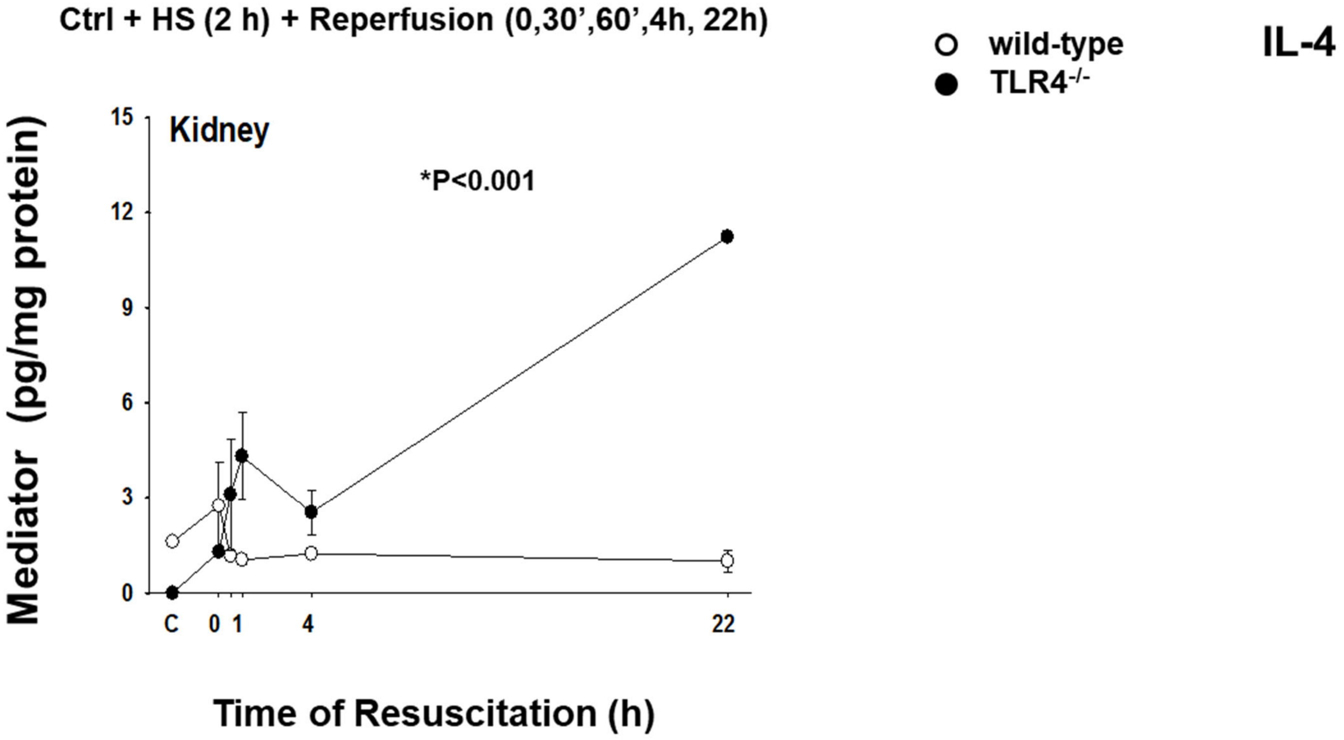
Ctrl + HS (2 h) + Reperfusion (0,30',60',4h, 22h)
wild-type
TLR4-/-
IL-4
Kidney
*P<0.001
Mediator (pg/mg protein)
15
12
9
6
3
0
C
0
1
4
22
Time of Resuscitation (h)

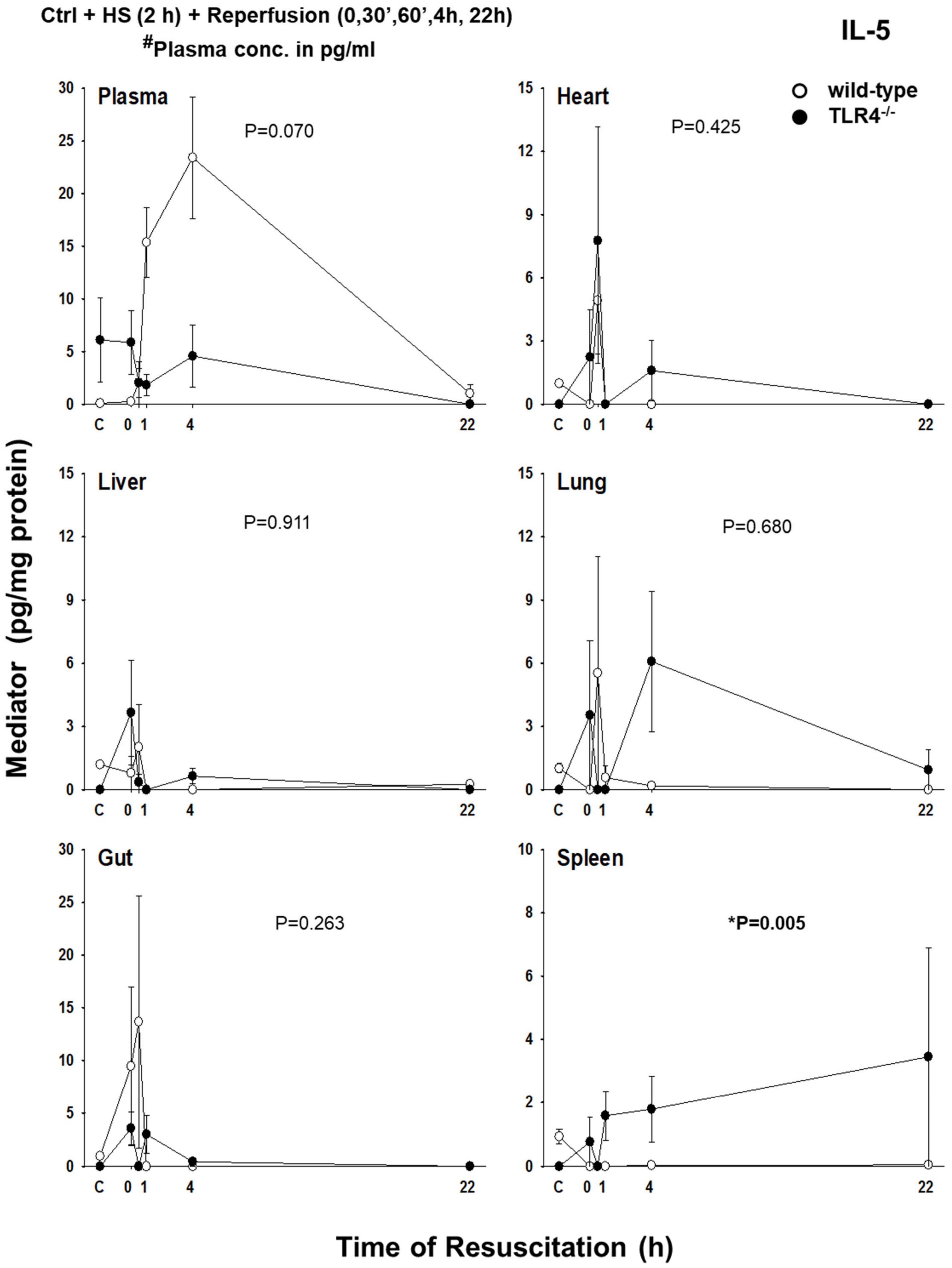
Ctrl + HS (2 h) + Reperfusion (0,30',60',4h, 22h)
#Plasma conc. in pg/ml
IL-5
wild-type
TLR4-/-
Plasma
P=0.070
Heart
P=0.425
Liver
P=0.911
Lung
P=0.680
Gut
P=0.263
Spleen
*P=0.005
Mediator (pg/mg protein)
Time of Resuscitation (h)
C
0
1
4
22

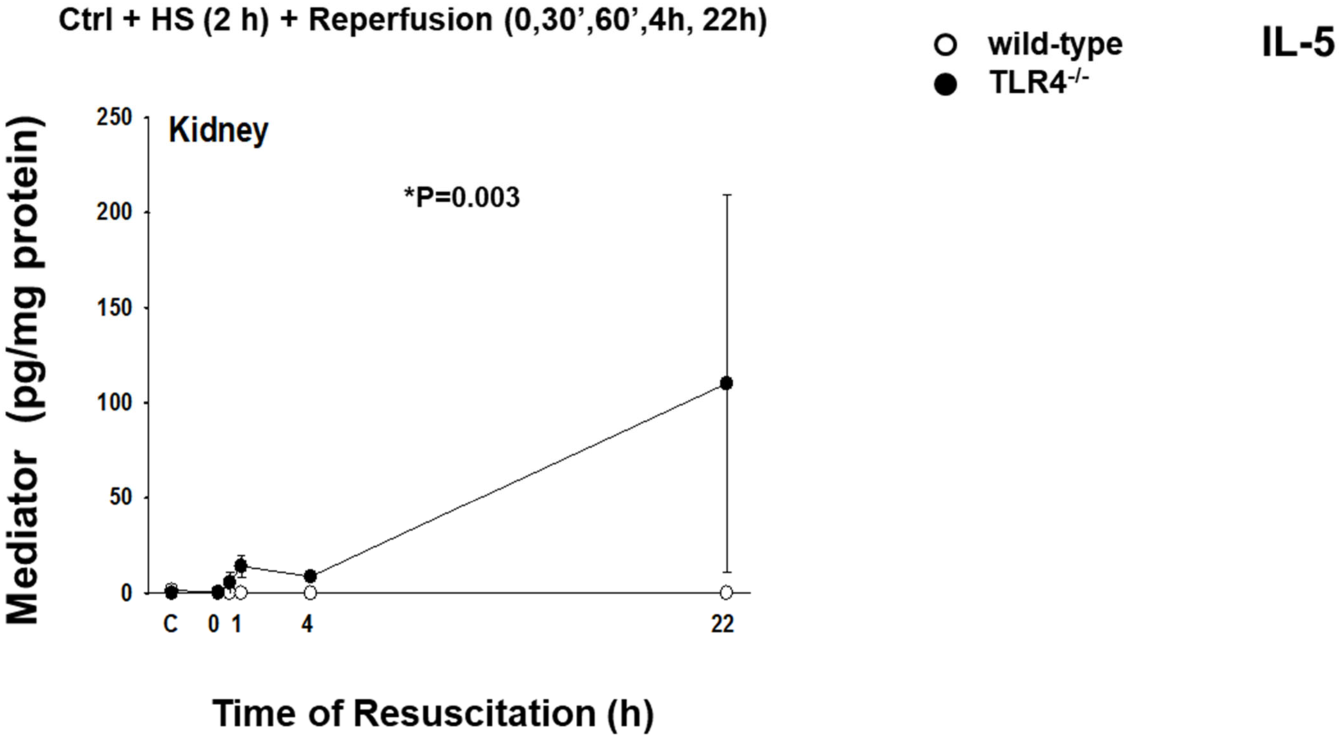
Ctrl + HS (2 h) + Reperfusion (0,30’,60’,4h, 22h)
wild-type
TLR4-/-
IL-5
Kidney
*P=0.003
Mediator (pg/mg protein)
250
200
150
100
50
0
C
0
1
4
22
Time of Resuscitation (h)

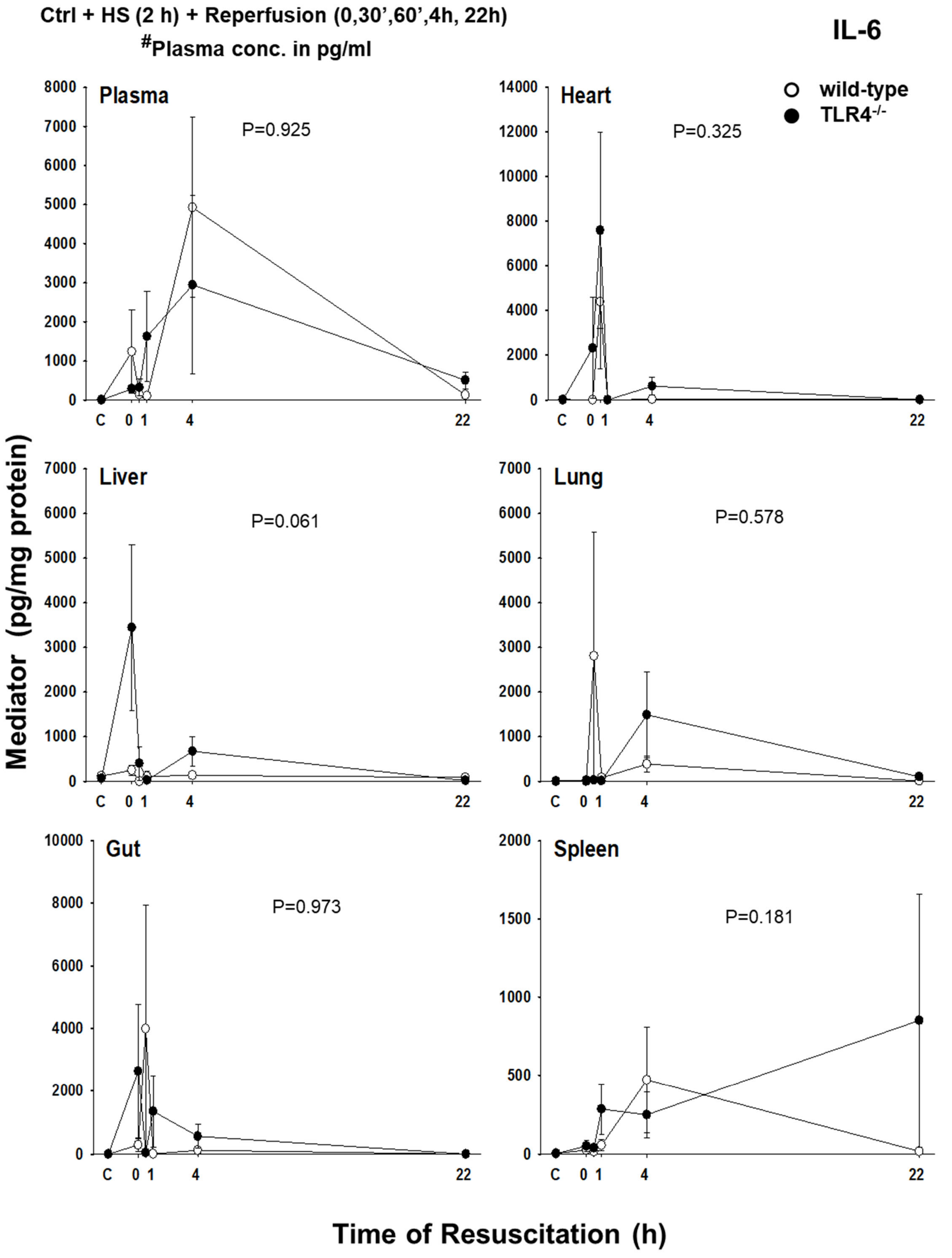

Ctrl + HS (2 h) + Reperfusion (0,30',60',4h, 22h)
#Plasma conc. in pg/ml
IL-6
wild-type
TLR4-/-
Plasma
P=0.925
Heart
P=0.325
Liver
P=0.061
Lung
P=0.578
Gut
P=0.973
Spleen
P=0.181
Mediator (pg/mg protein)
Time of Resuscitation (h)
C
0
1
4
22

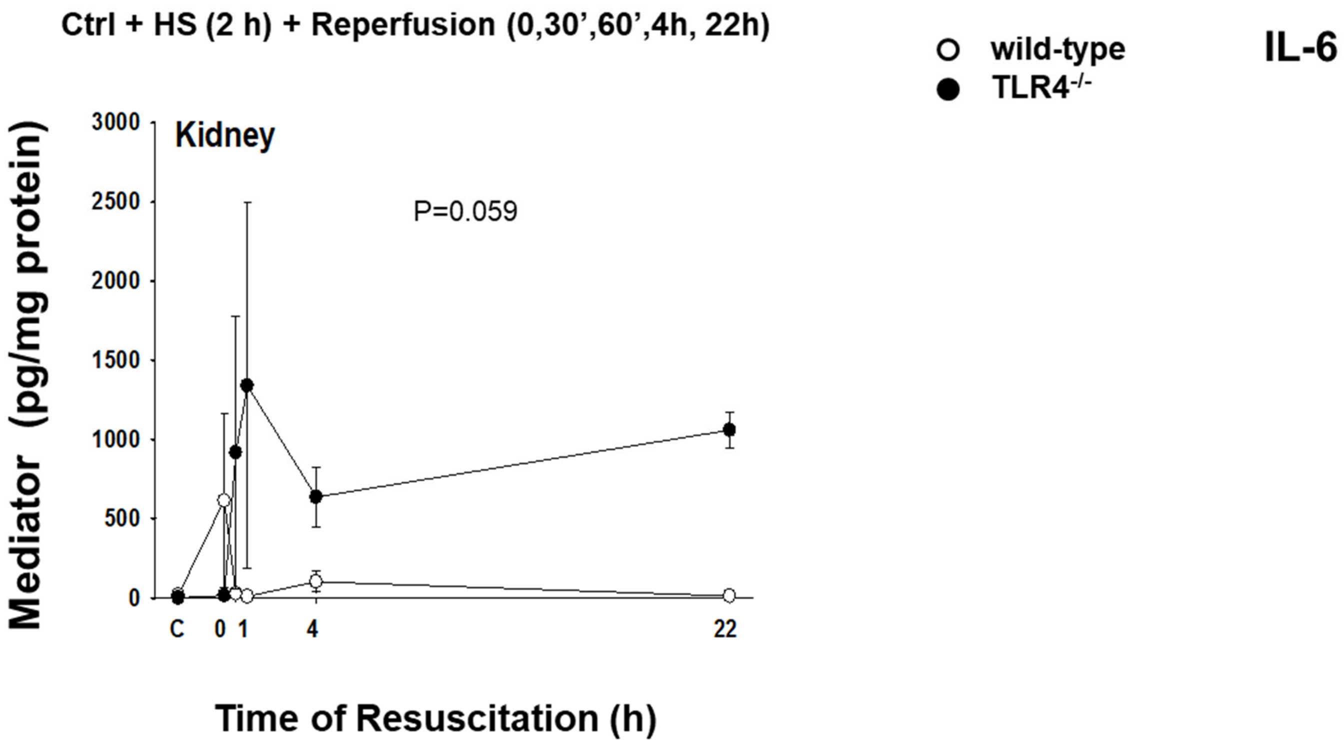

Ctrl + HS (2 h) + Reperfusion (0,30’,60’,4h, 22h)
wild-type
TLR4-/-
IL-6
Kidney
P=0.059
Mediator (pg/mg protein)
3000
2500
2000
1500
1000
500
0
C
0
1
4
22
Time of Resuscitation (h)

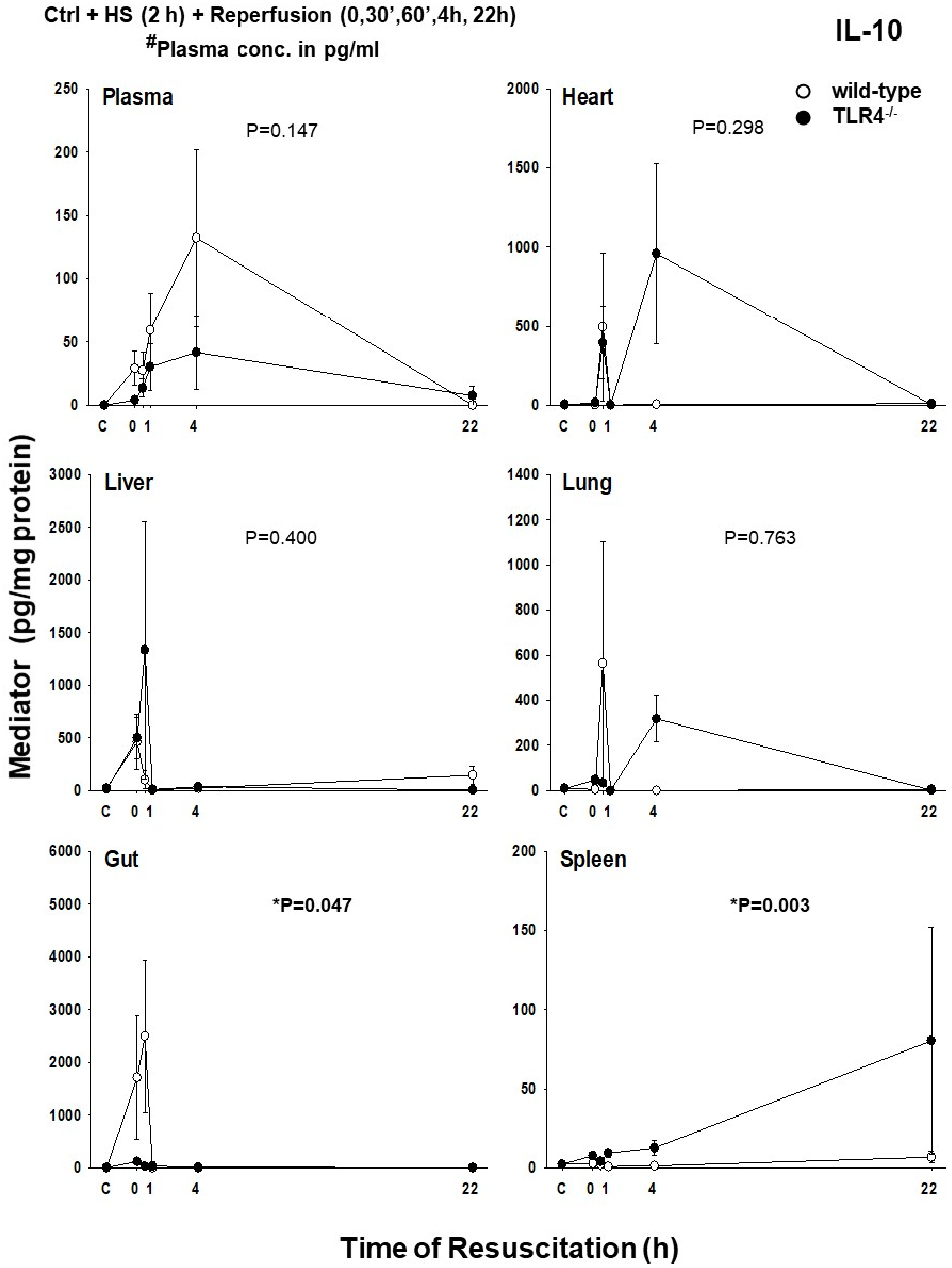
Ctrl + HS (2 h) + Reperfusion (0,30',60',4h, 22h)
#Plasma conc. in pg/ml
IL-10
wild-type
TLR4-/-
Plasma
P=0.147
Heart
P=0.298
Liver
P=0.400
Lung
P=0.763
Gut
*P=0.047
Spleen
*P=0.003
Mediator (pg/mg protein)
Time of Resuscitation (h)
C 0 1 4 22

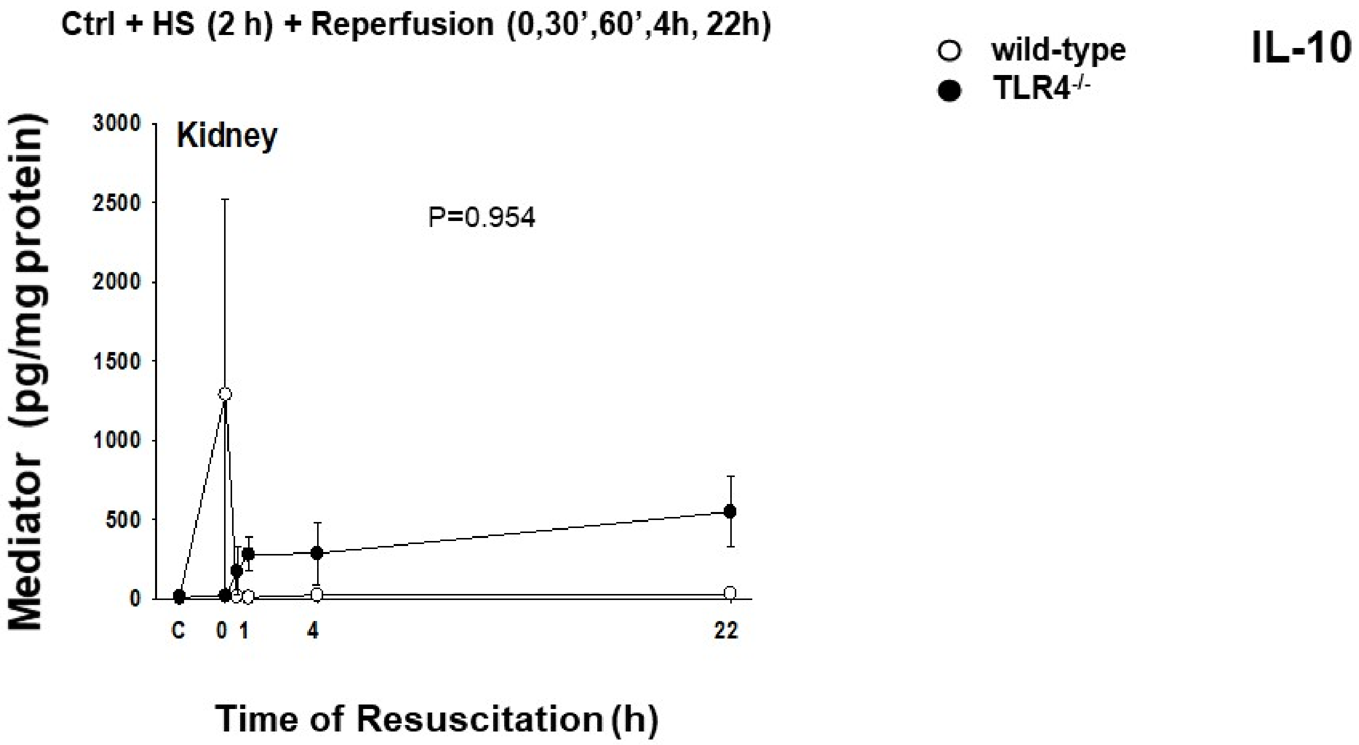
Ctrl + HS (2 h) + Reperfusion (0,30',60',4h, 22h)
wild-type
TLR4-/-
IL-10
Kidney
P=0.954
Mediator (pg/mg protein)
3000
2500
2000
1500
1000
500
0
C
0 1
4
22
Time of Resuscitation (h)

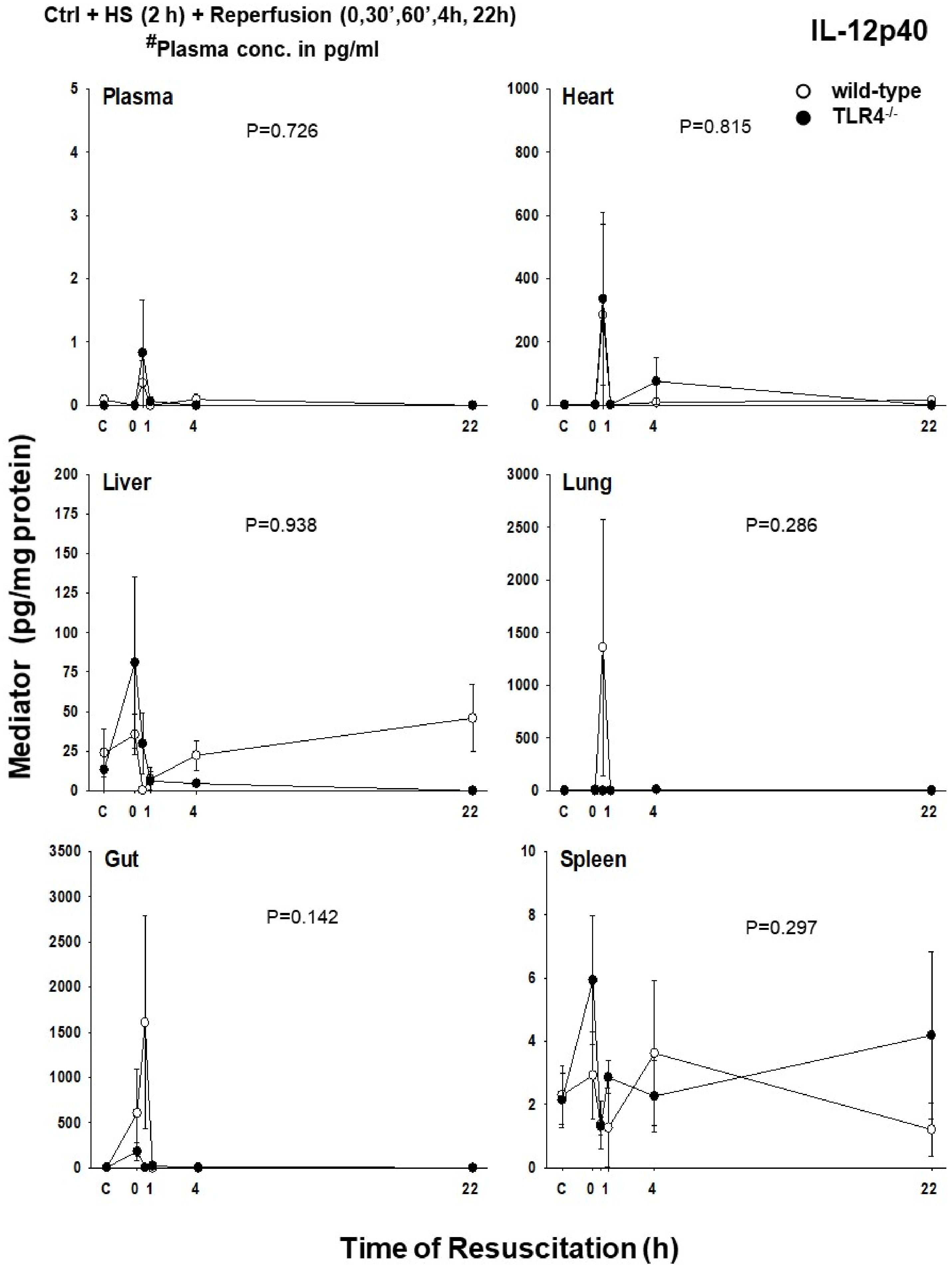
Ctrl + HS (2 h) + Reperfusion (0,30',60',4h, 22h)
#Plasma conc. in pg/ml
IL-12p40
wild-type
TLR4-/-
Plasma
P=0.726
Heart
P=0.815
Liver
P=0.938
Lung
P=0.286
Gut
P=0.142
Spleen
P=0.297
Mediator (pg/mg protein)
C
0
1
4
22
Time of Resuscitation (h)

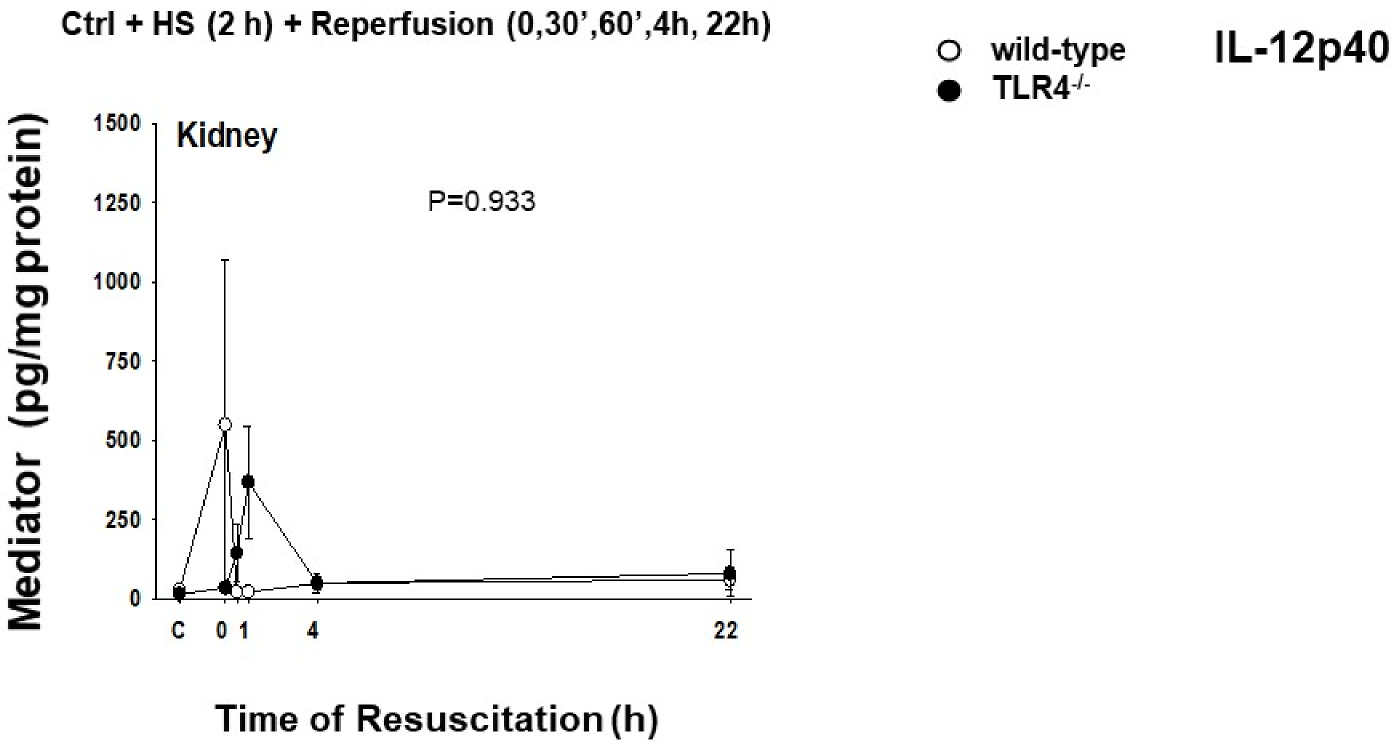

Ctrl + HS (2 h) + Reperfusion (0,30',60',4h, 22h)
wild-type
TLR4-/-
IL-12p40
Kidney
P=0.933
Mediator (pg/mg protein)
1500
1250
1000
750
500
250
0
C
0
1
4
22
Time of Resuscitation (h)

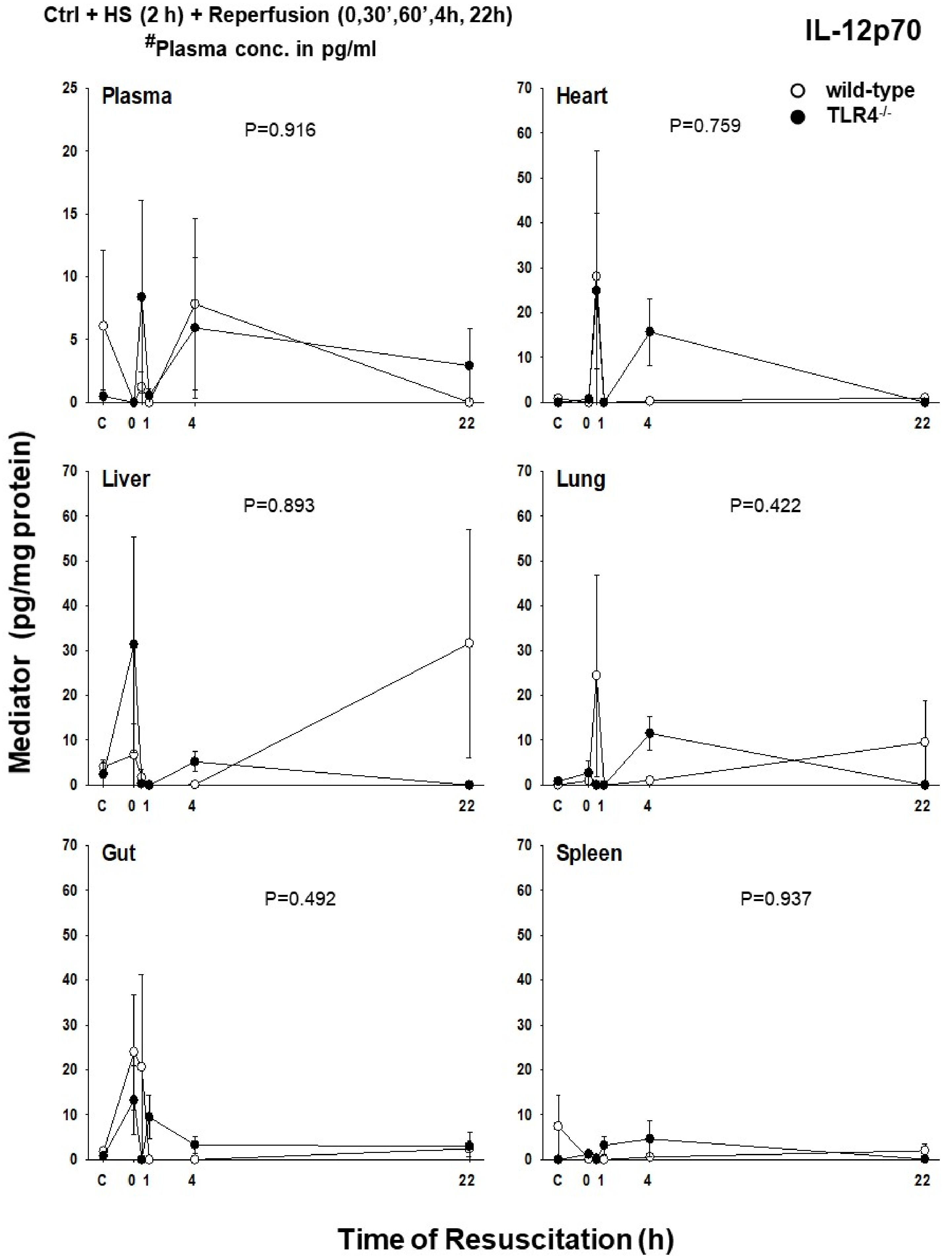
Ctrl + HS (2 h) + Reperfusion (0,30',60',4h, 22h)
#Plasma conc. in pg/ml
IL-12p70
wild-type
TLR4-/-
Plasma
P=0.916
Heart
P=0.759
Liver
P=0.893
Lung
P=0.422
Gut
P=0.492
Spleen
P=0.937
C 0 1 4 22
Mediator (pg/mg protein)
Time of Resuscitation (h)

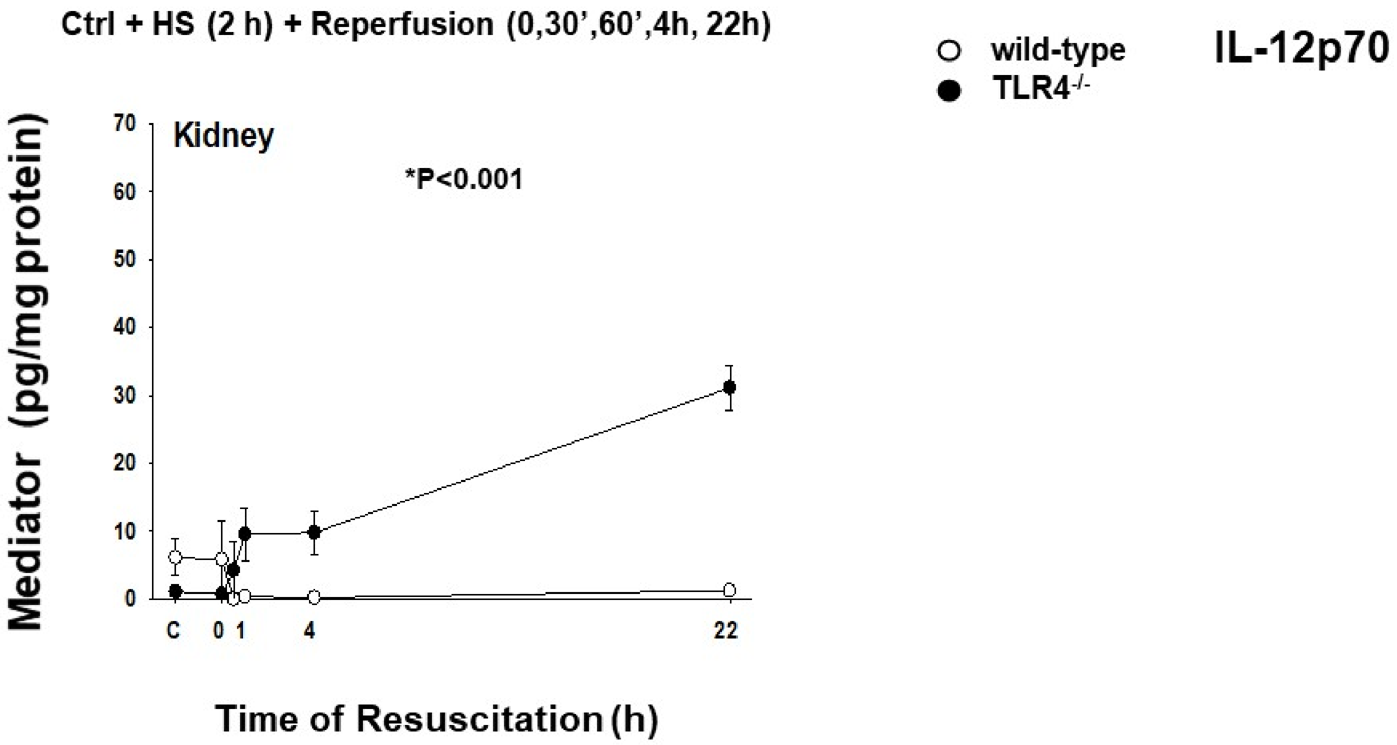
Ctrl + HS (2 h) + Reperfusion (0,30',60',4h, 22h)
wild-type
TLR4-/-
IL-12p70
Kidney
*P<0.001
Mediator (pg/mg protein)
70
60
50
40
30
20
10
0
C
0
1
4
22
Time of Resuscitation (h)

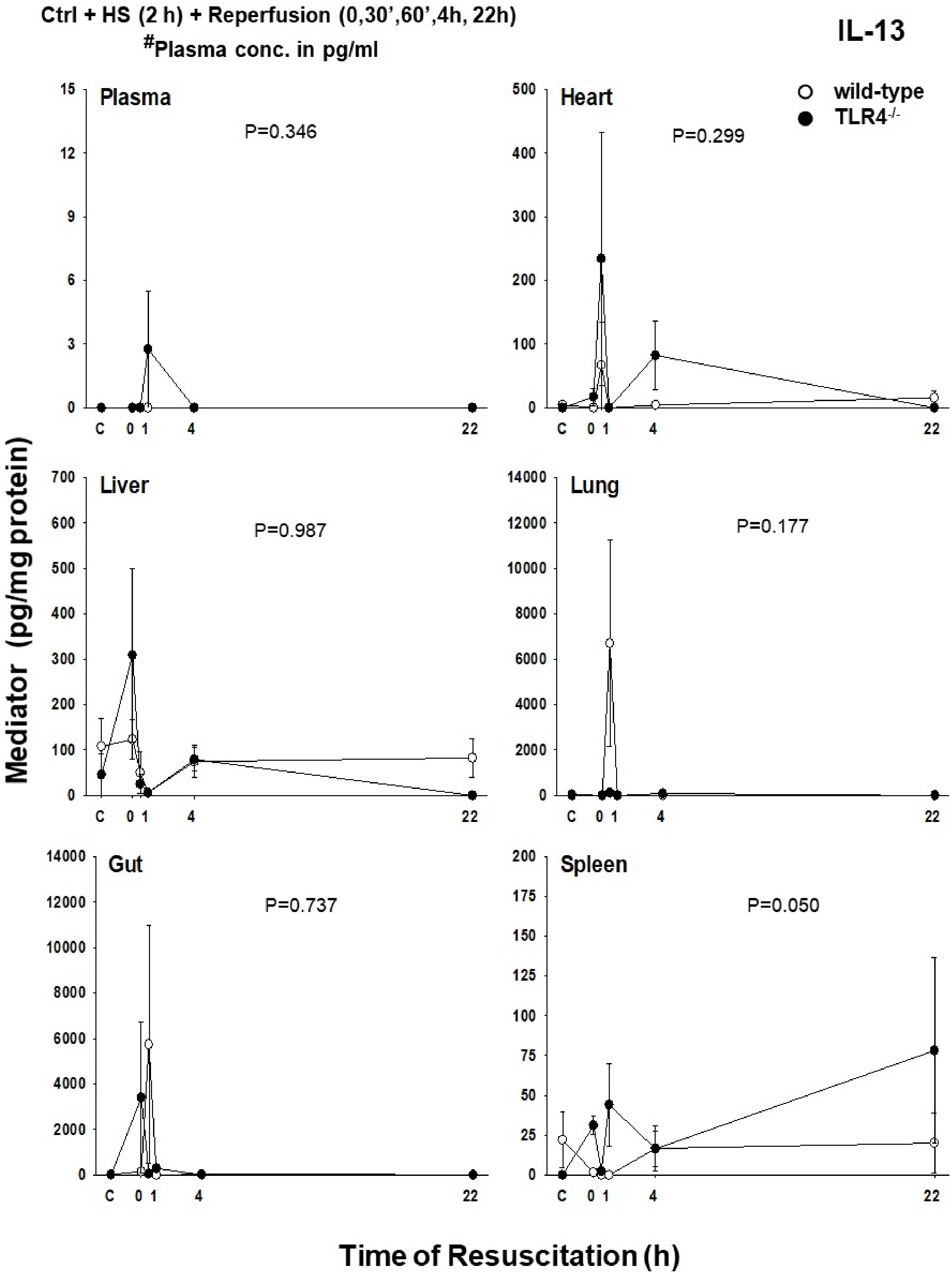
Ctrl + HS (2 h) + Reperfusion (0,30',60',4h, 22h)
#Plasma conc. in pg/ml
IL-13
wild-type
TLR4-/-
Plasma
P=0.346
Heart
P=0.299
Liver
P=0.987
Lung
P=0.177
Gut
P=0.737
Spleen
P=0.050
Mediator (pg/mg protein)
Time of Resuscitation (h)

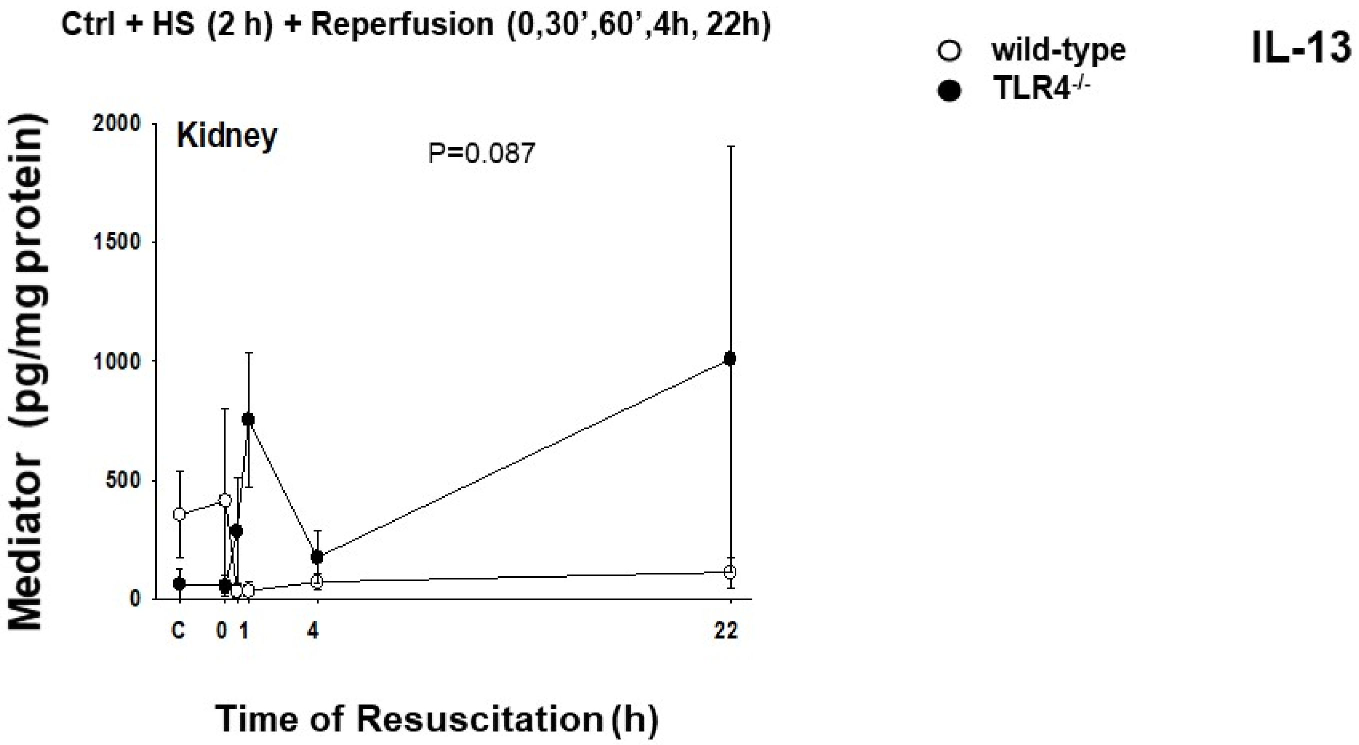

Ctrl + HS (2 h) + Reperfusion (0,30',60',4h, 22h)
wild-type
TLR4-/-
IL-13
Kidney
P=0.087
Mediator (pg/mg protein)
2000
1500
1000
500
0
C
0
1
4
22
Time of Resuscitation (h)

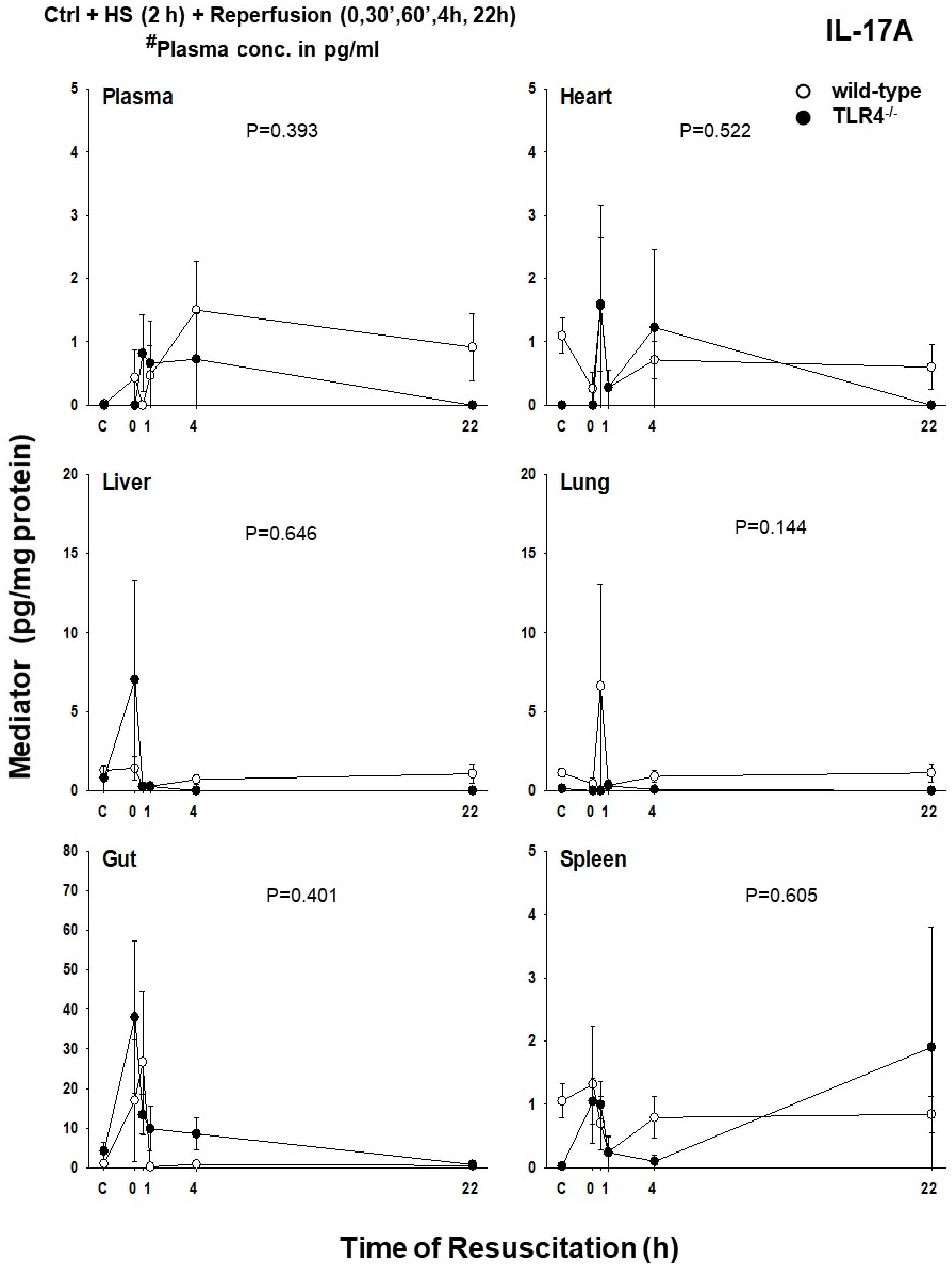
Ctrl + HS (2 h) + Reperfusion (0,30',60',4h, 22h)
#Plasma conc. in pg/ml
IL-17A
wild-type
TLR4-/-
Plasma
P=0.393
Heart
P=0.522
Liver
P=0.646
Lung
P=0.144
Gut
P=0.401
Spleen
P=0.605
Mediator (pg/mg protein)
Time of Resuscitation (h)
C
0
1
4
22

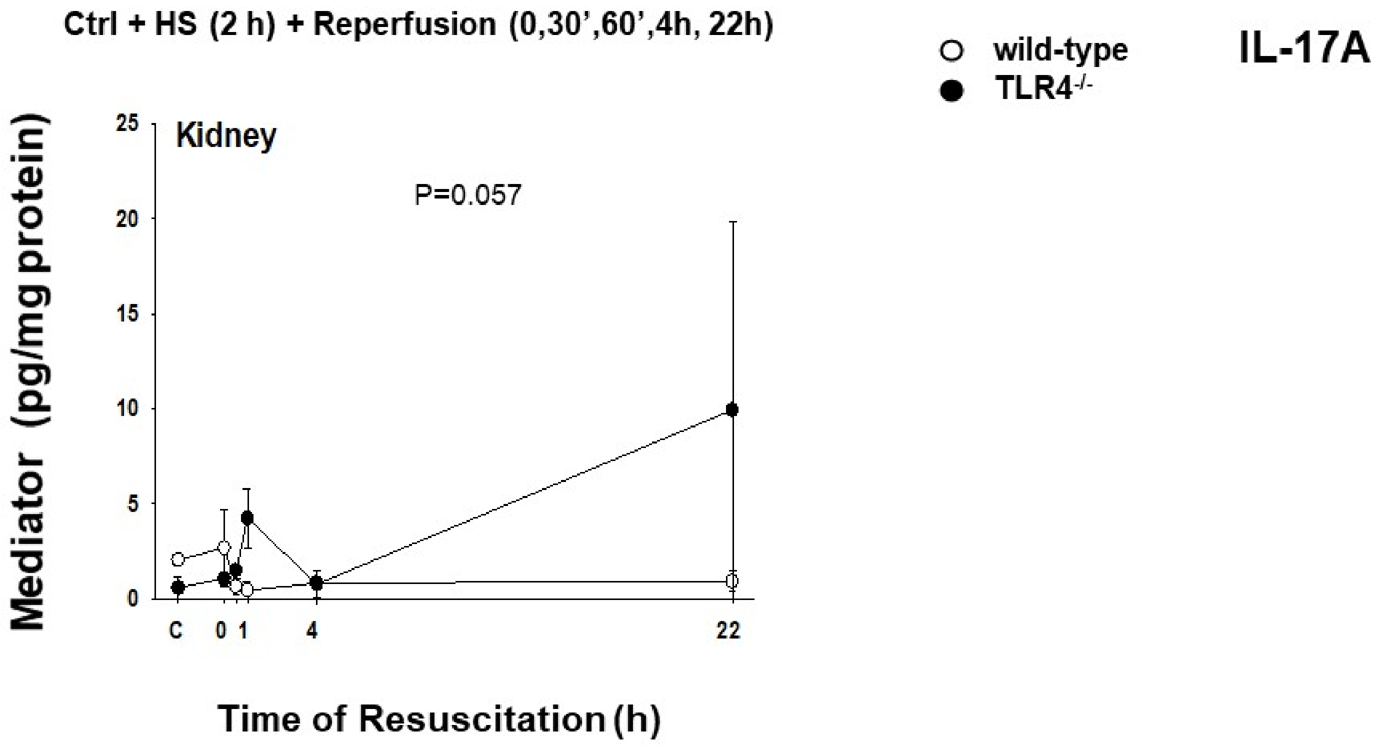
Ctrl + HS (2 h) + Reperfusion (0,30',60',4h, 22h)
wild-type
TLR4-/-
IL-17A
Kidney
P=0.057
Mediator (pg/mg protein)
25
20
15
10
5
0
C
0
1
4
22
Time of Resuscitation (h)

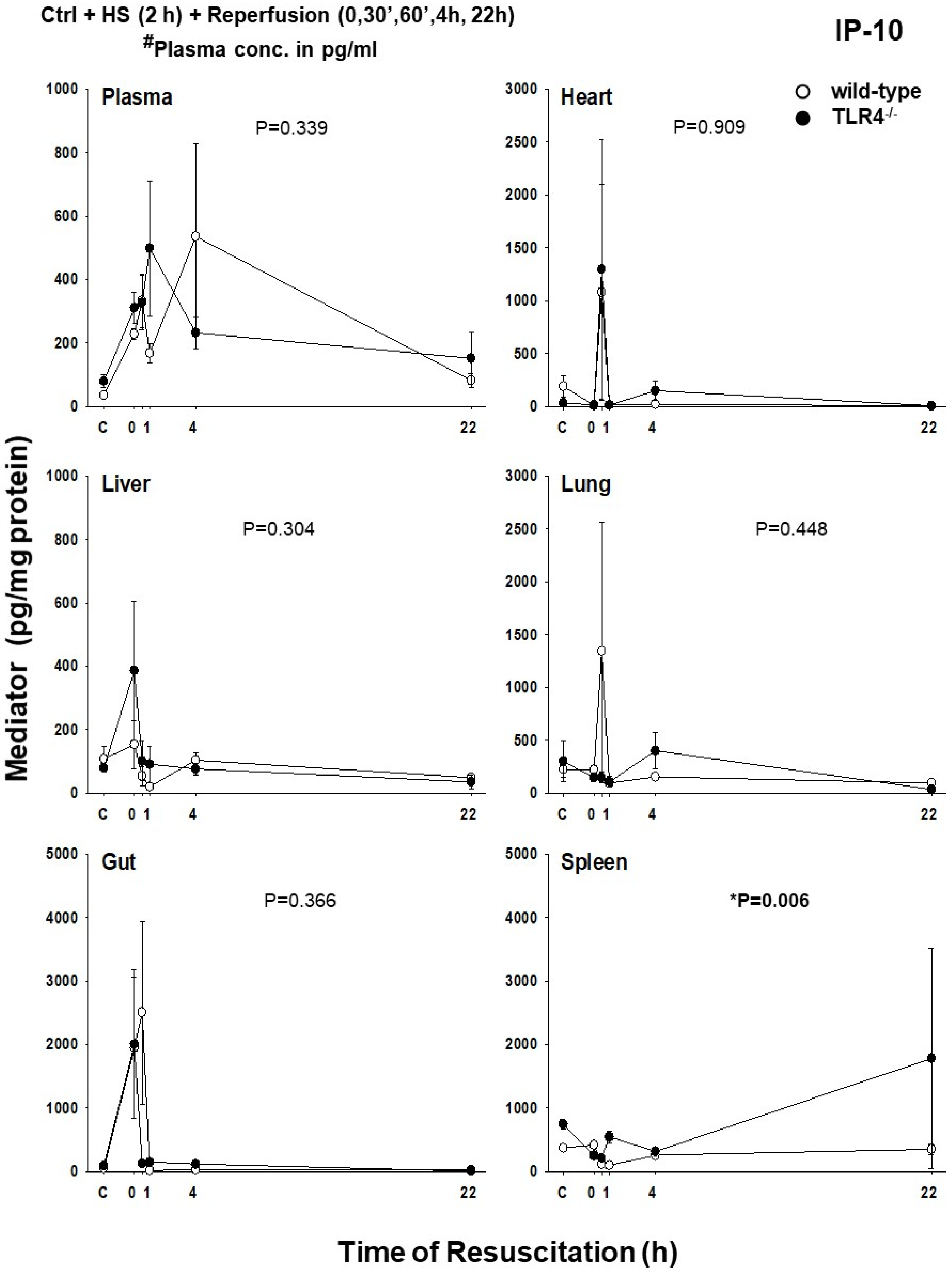

Ctrl + HS (2 h) + Reperfusion (0,30',60',4h, 22h)
#Plasma conc. in pg/ml
IP-10
wild-type
TLR4-/-
Plasma
P=0.339
Heart
P=0.909
Liver
P=0.304
Lung
P=0.448
Gut
P=0.366
Spleen
*P=0.006
Mediator (pg/mg protein)
Time of Resuscitation (h)
C
0
1
4
22

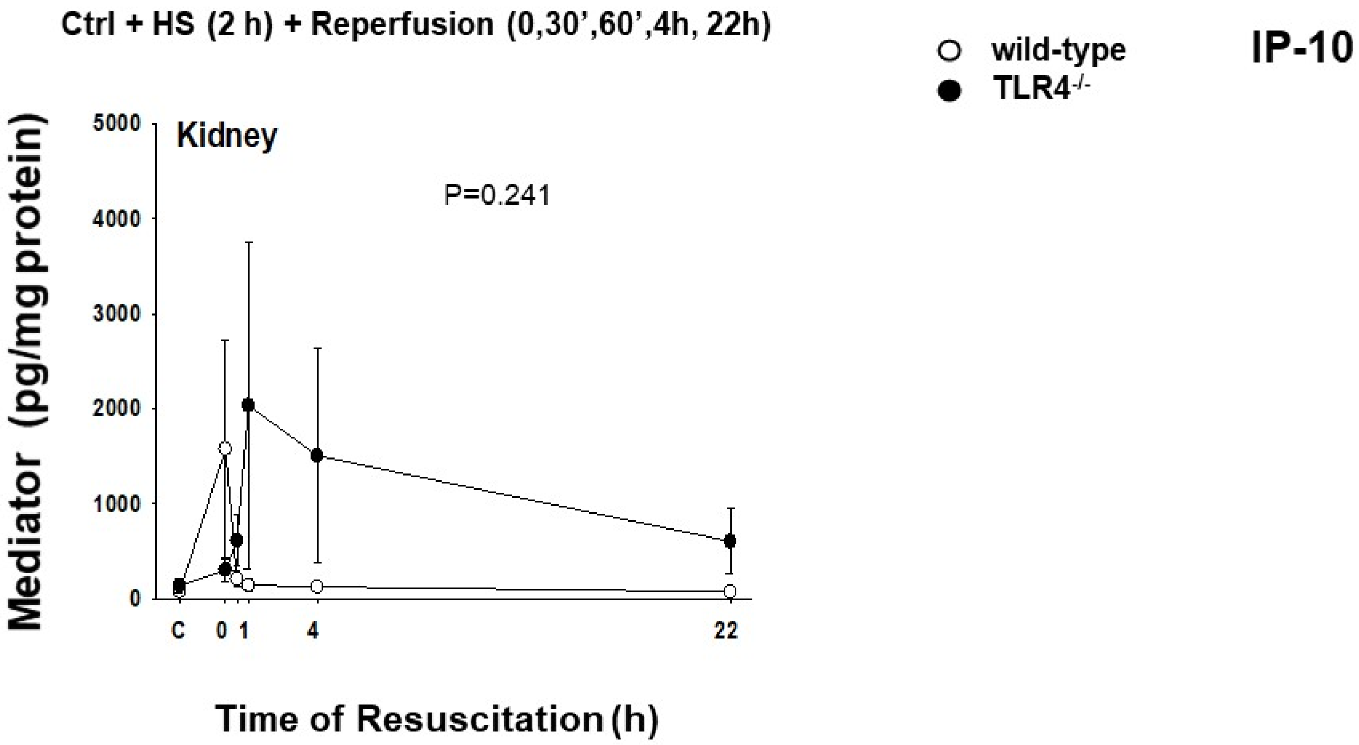
Ctrl + HS (2 h) + Reperfusion (0,30',60',4h, 22h)
wild-type
TLR4-/-
IP-10
Kidney
P=0.241
Mediator (pg/mg protein)
5000
4000
3000
2000
1000
0
C
0
1
4
22
Time of Resuscitation (h)

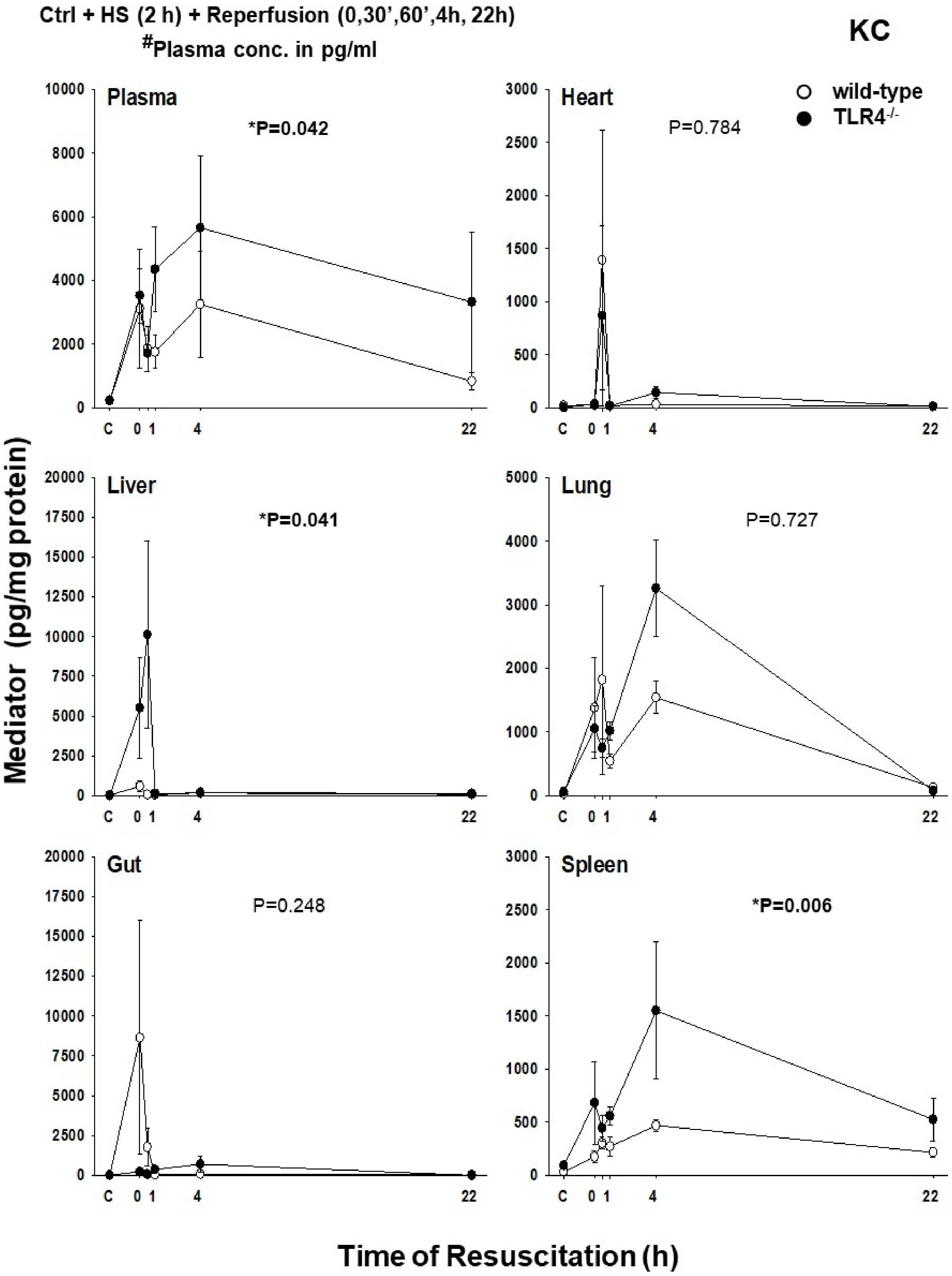

Ctrl + HS (2 h) + Reperfusion (0,30',60',4h, 22h)
#Plasma conc. in pg/ml
KC
wild-type
TLR4-/-
Plasma
*P=0.042
Heart
P=0.784
Liver
*P=0.041
Lung
P=0.727
Gut
P=0.248
Spleen
*P=0.006
Mediator (pg/mg protein)
Time of Resuscitation (h)

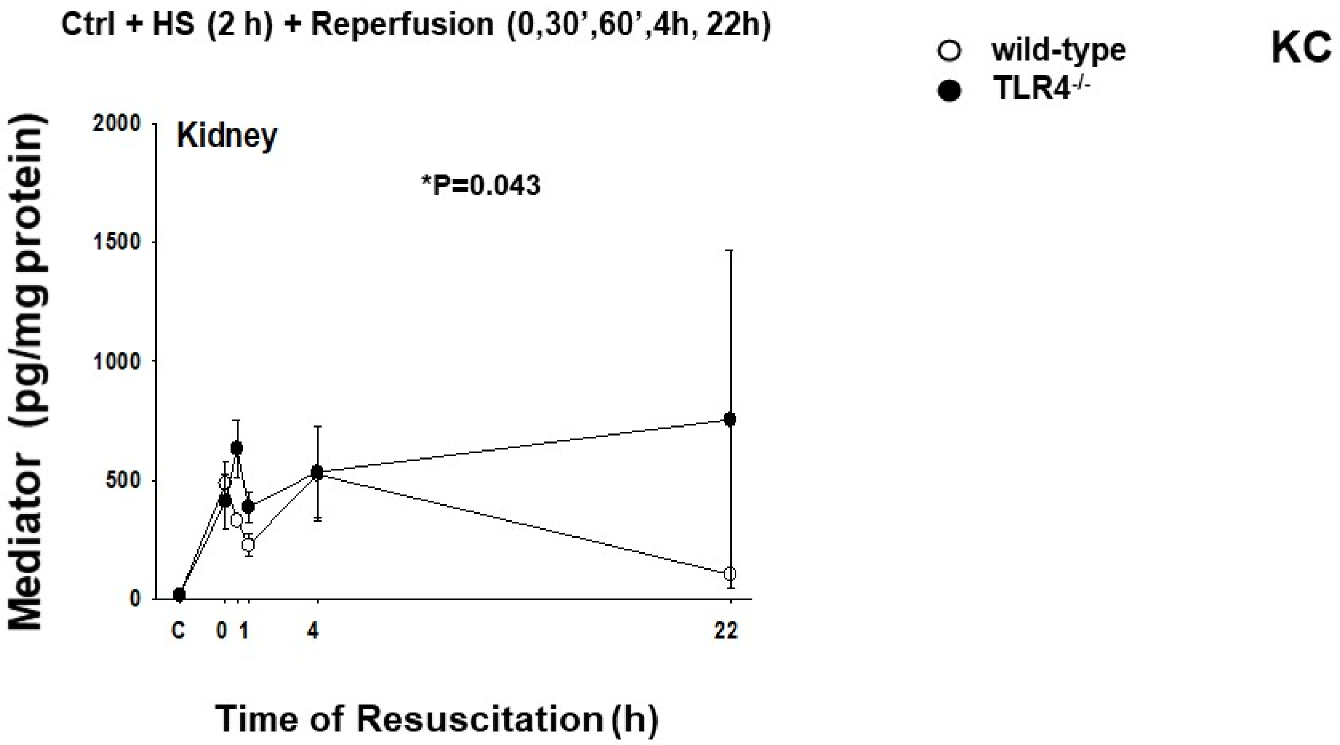

Ctrl + HS (2 h) + Reperfusion (0,30',60',4h, 22h)
wild-type
TLR4-/-
KC
Kidney
*P=0.043
Mediator (pg/mg protein)
2000
1500
1000
500
0
C
0
1
4
22
Time of Resuscitation (h)

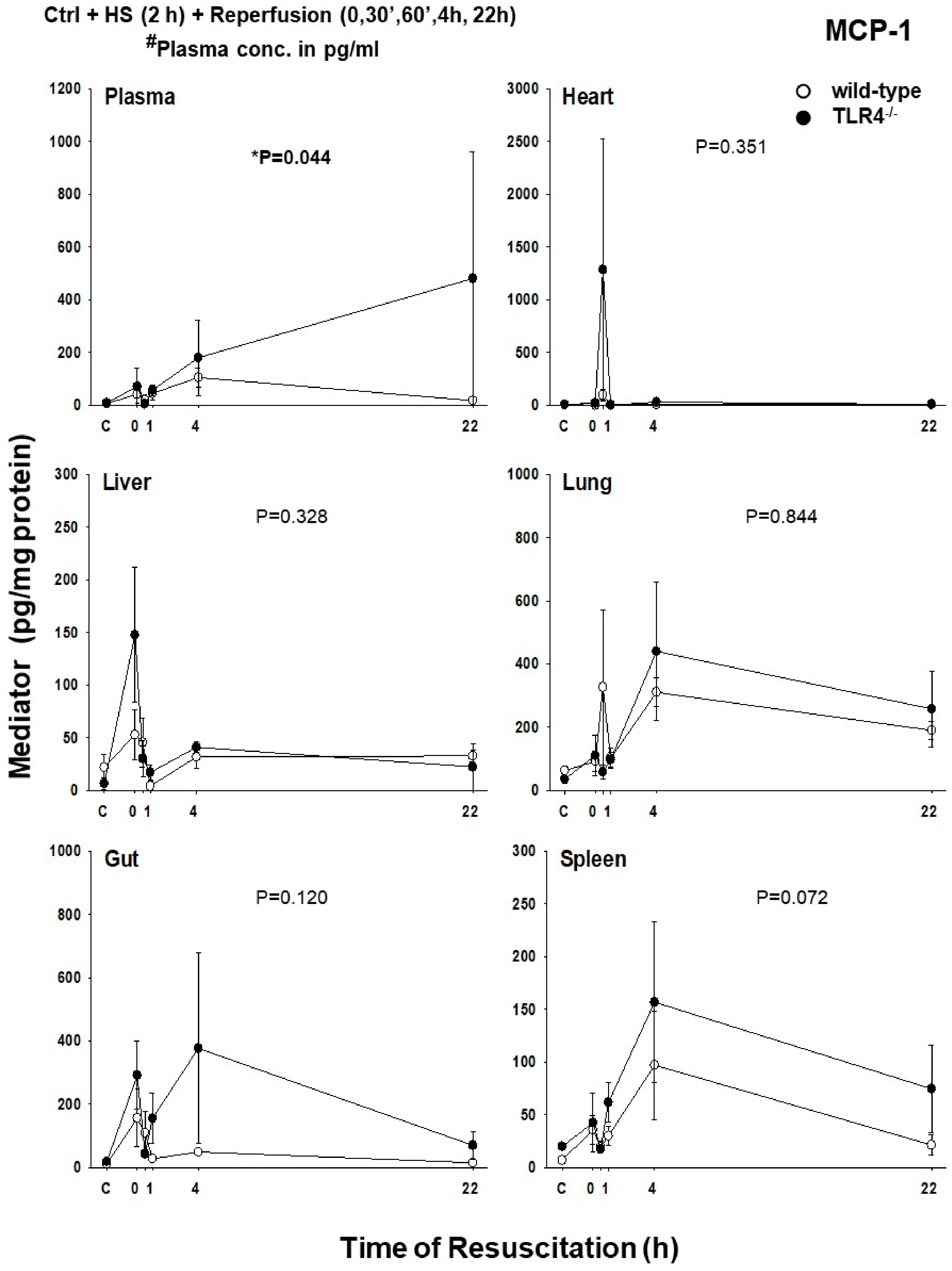
Ctrl + HS (2 h) + Reperfusion (0,30',60',4h, 22h)
#Plasma conc. in pg/ml
MCP-1
wild-type
TLR4-/-
Plasma
*P=0.044
Heart
P=0.351
Liver
P=0.328
Lung
P=0.844
Gut
P=0.120
Spleen
P=0.072
Mediator (pg/mg protein)
Time of Resuscitation (h)
C
0 1
4
22

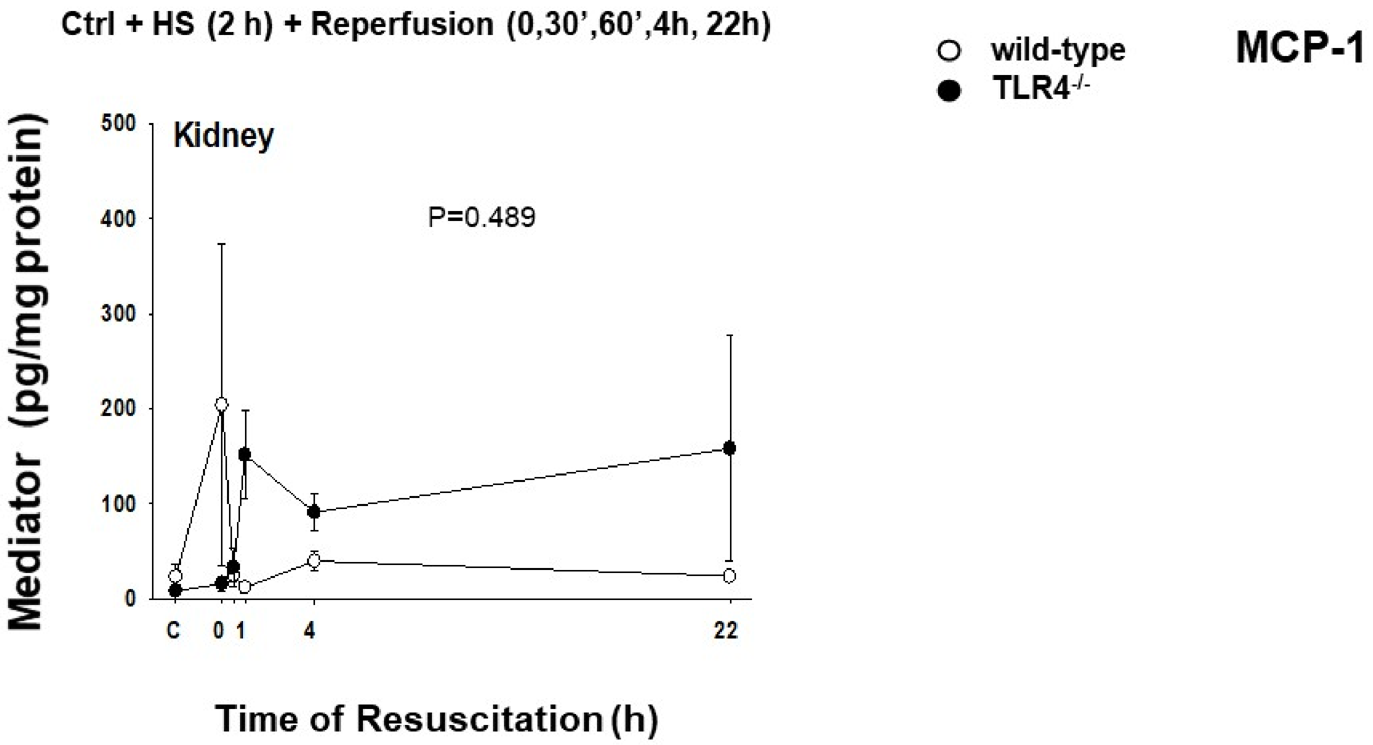
Ctrl + HS (2 h) + Reperfusion (0,30',60',4h, 22h)
wild-type
TLR4-/-
MCP-1
Kidney
P=0.489
Mediator (pg/mg protein)
0
100
200
300
400
500
C
0
1
4
22
Time of Resuscitation (h)

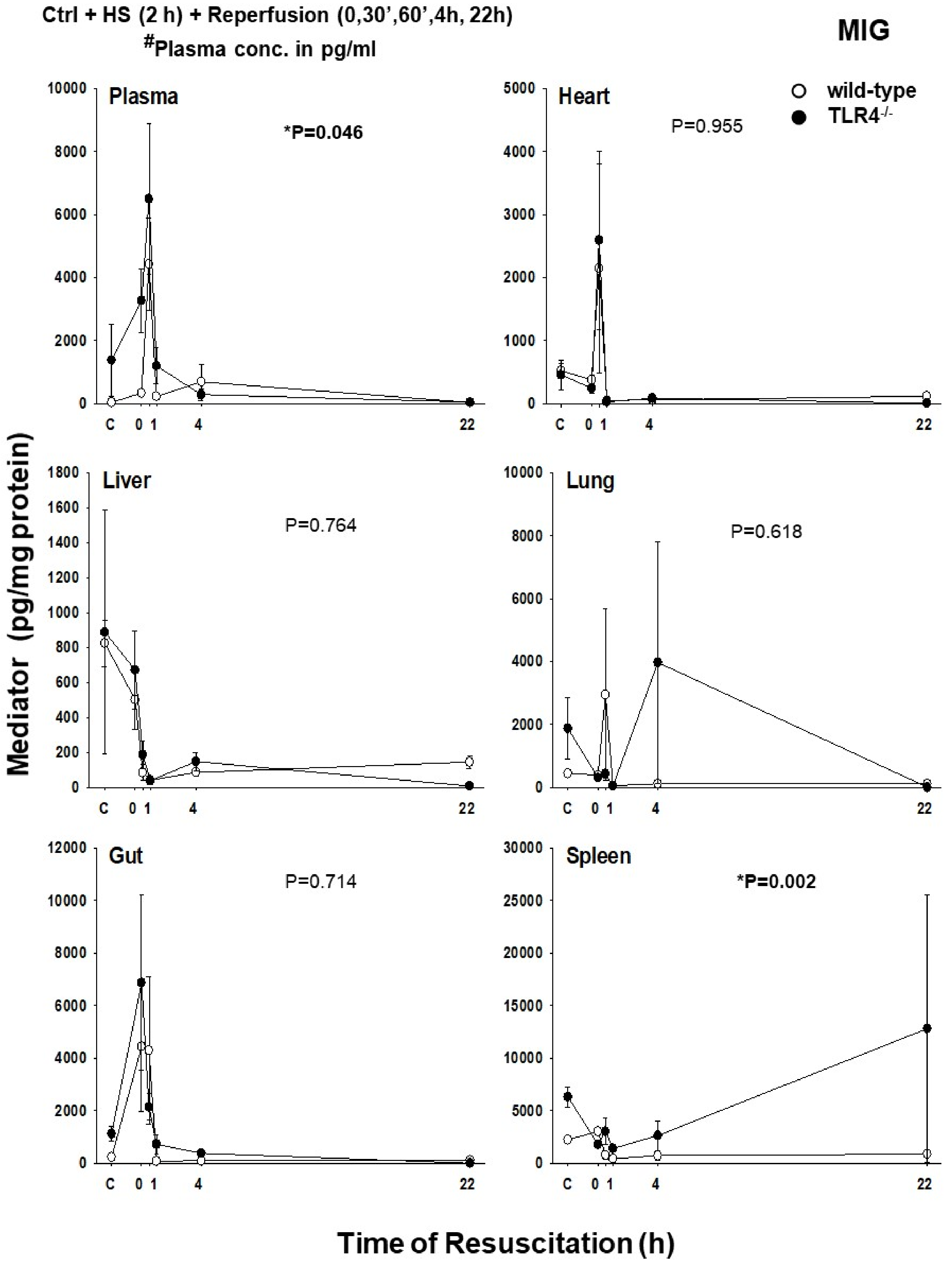
Ctrl + HS (2 h) + Reperfusion (0,30',60',4h, 22h)
#Plasma conc. in pg/ml
MIG
wild-type
TLR4-/-
Plasma
*P=0.046
Heart
P=0.955
Liver
P=0.764
Lung
P=0.618
Gut
P=0.714
Spleen
*P=0.002
Mediator (pg/mg protein)
Time of Resuscitation (h)

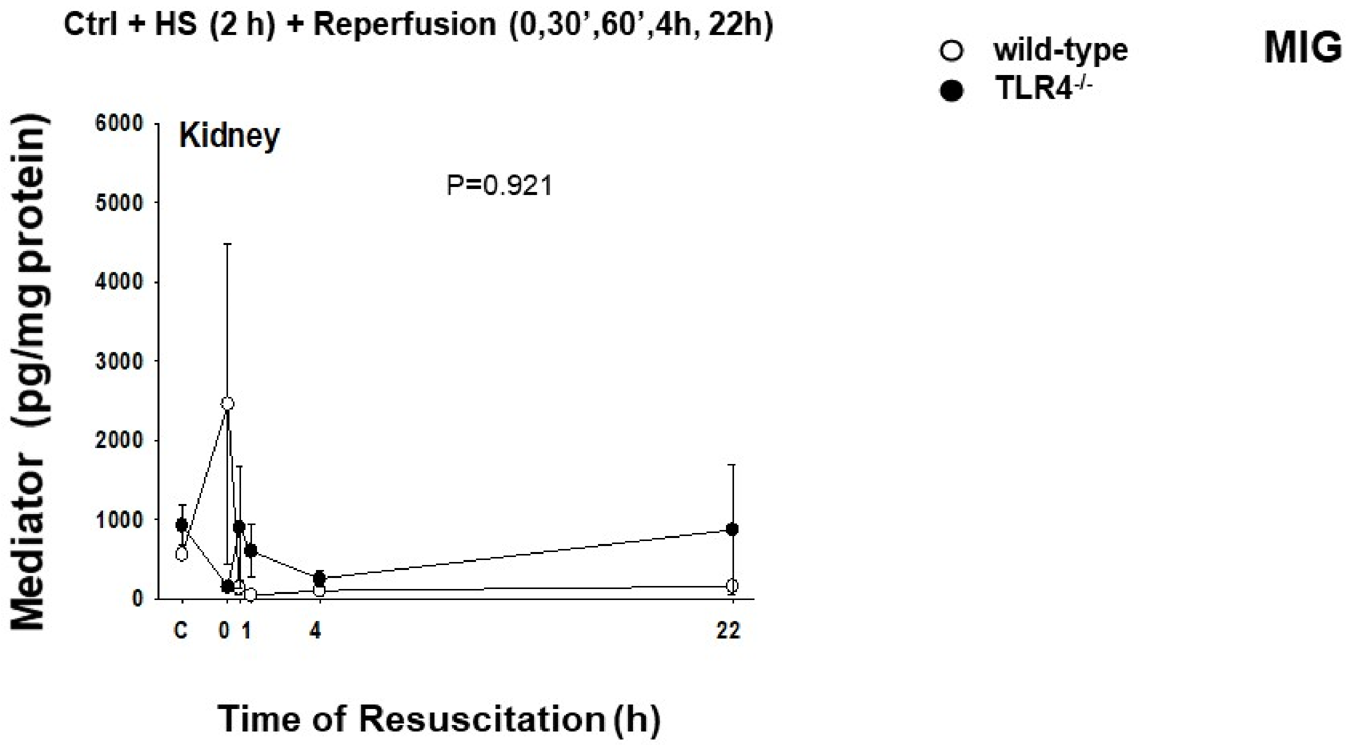
Ctrl + HS (2 h) + Reperfusion (0,30',60',4h, 22h)
wild-type
TLR4-/-
MIG
Kidney
P=0.921
Mediator (pg/mg protein)
6000
5000
4000
3000
2000
1000
0
C
0
1
4
22
Time of Resuscitation (h)

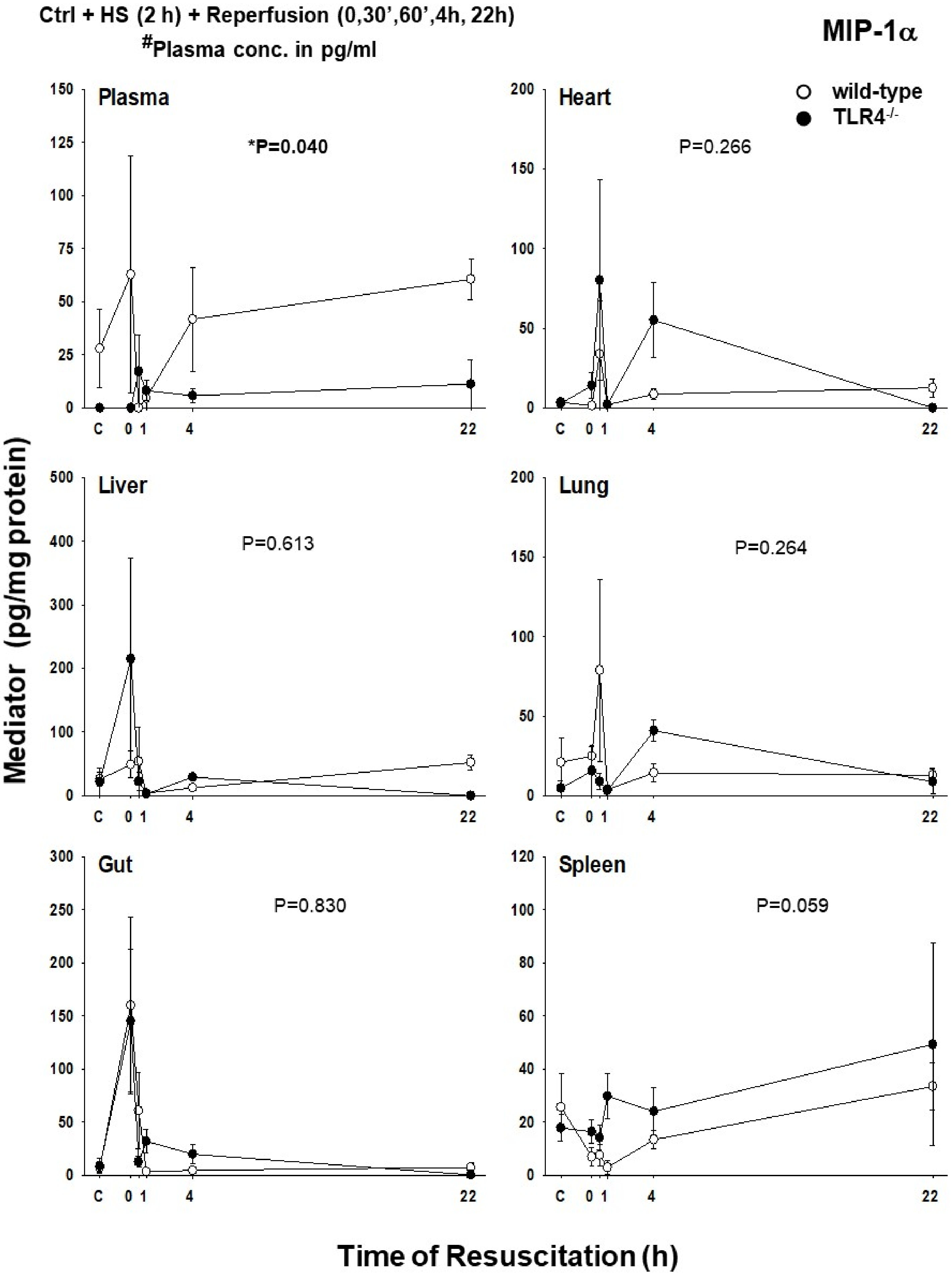
Ctrl + HS (2 h) + Reperfusion (0,30',60',4h, 22h)
#Plasma conc. in pg/ml
MIP-1α
wild-type
TLR4-/-
Plasma
*P=0.040
Heart
P=0.266
Liver
P=0.613
Lung
P=0.264
Gut
P=0.830
Spleen
P=0.059
Mediator (pg/mg protein)
Time of Resuscitation (h)

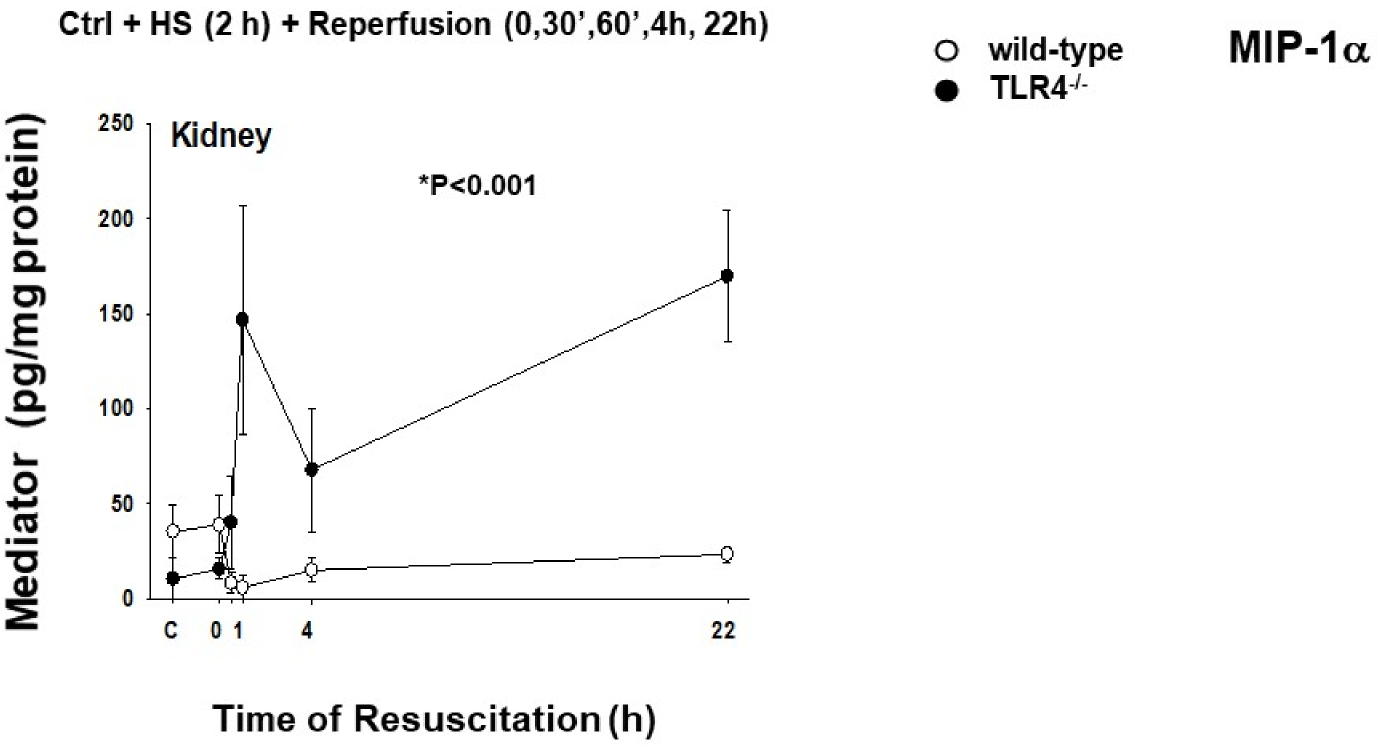
Ctrl + HS (2 h) + Reperfusion (0,30',60',4h, 22h)
wild-type
TLR4-/-
MIP-1α
Kidney
*P<0.001
Mediator (pg/mg protein)
250
200
150
100
50
0
C
0
1
4
22
Time of Resuscitation (h)

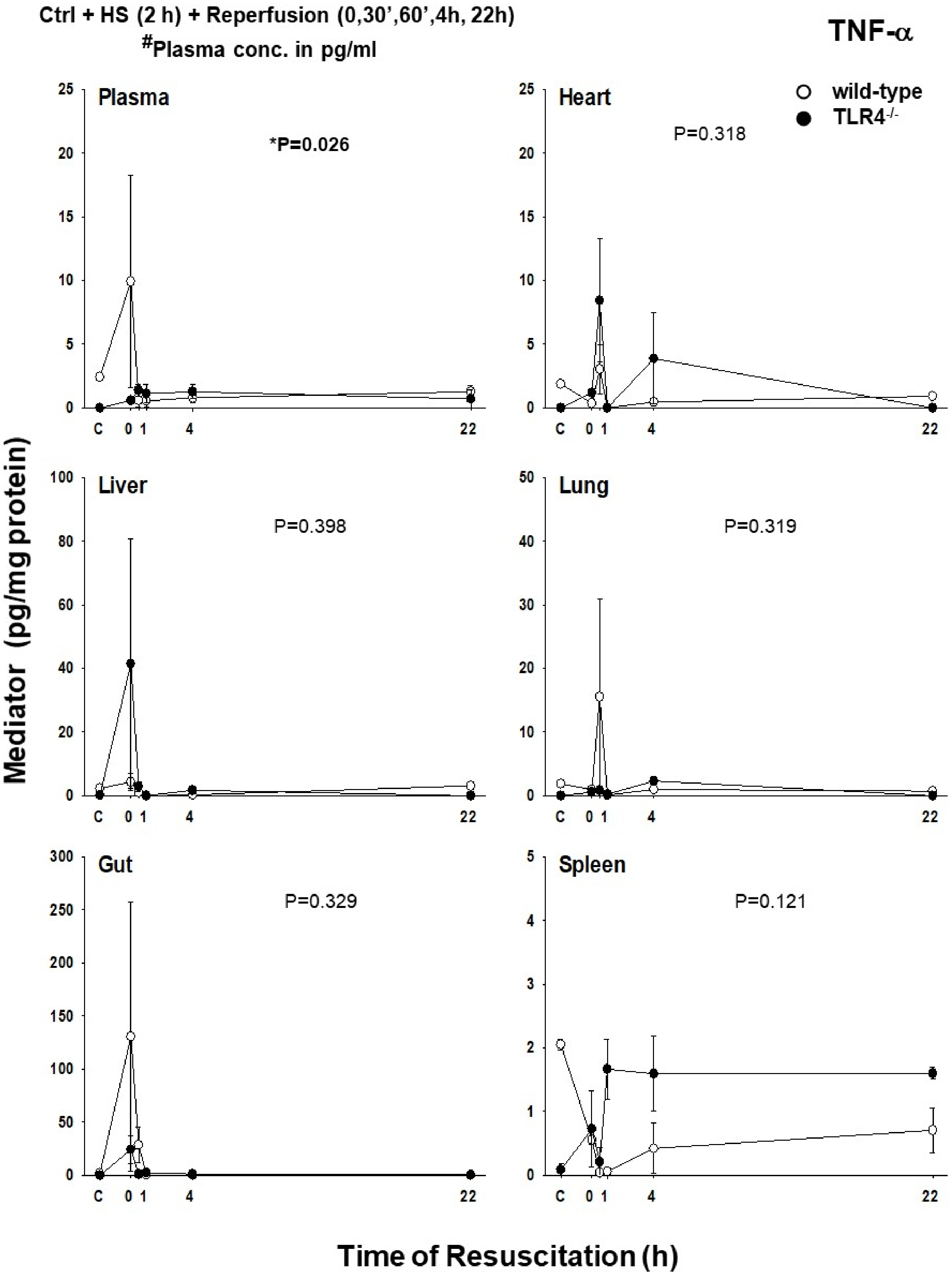

Ctrl + HS (2 h) + Reperfusion (0,30',60',4h, 22h)
#Plasma conc. in pg/ml
TNF-α
wild-type
TLR4-/-
Plasma
*P=0.026
Heart
P=0.318
Liver
P=0.398
Lung
P=0.319
Gut
P=0.329
Spleen
P=0.121
Mediator (pg/mg protein)
Time of Resuscitation (h)
C
0
1
4
22

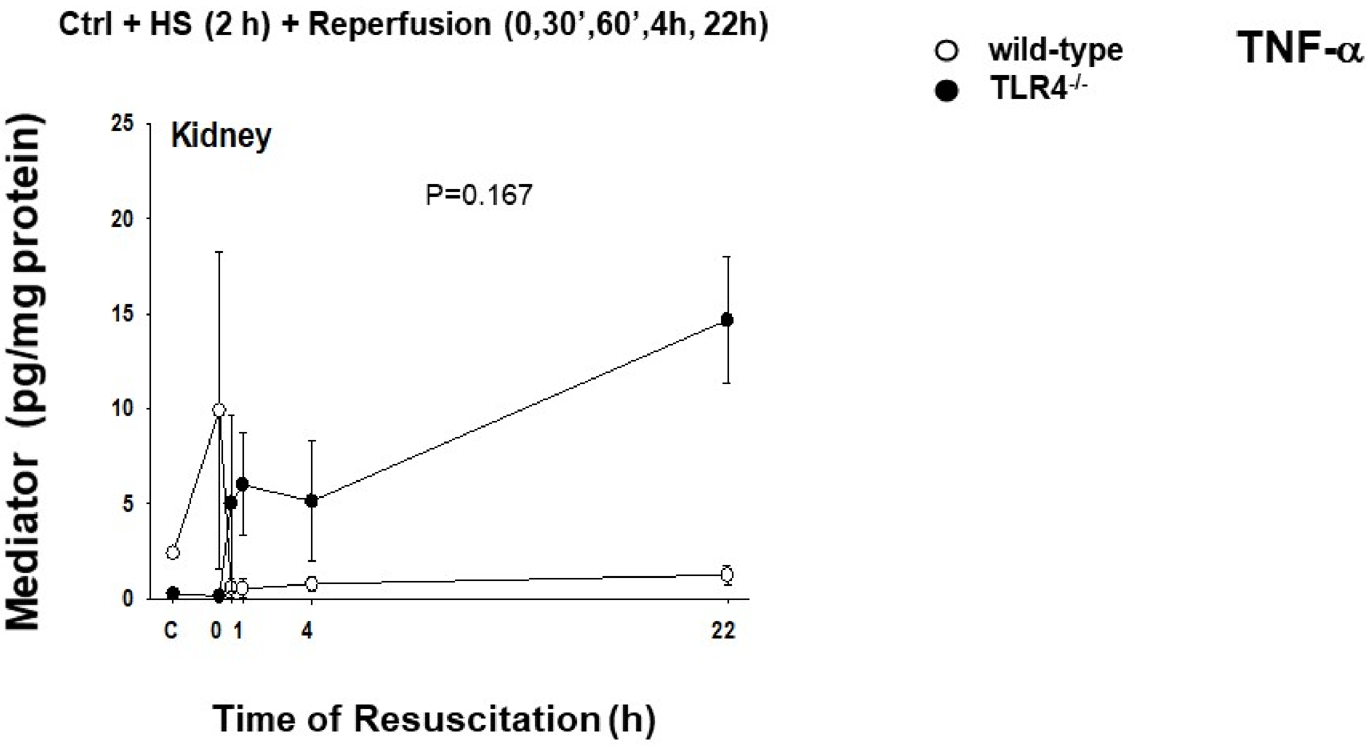
Ctrl + HS (2 h) + Reperfusion (0,30',60',4h, 22h)
wild-type
TLR4-/-
TNF-α
Kidney
P=0.167
Mediator (pg/mg protein)
25
20
15
10
5
0
C
0
1
4
22
Time of Resuscitation (h)

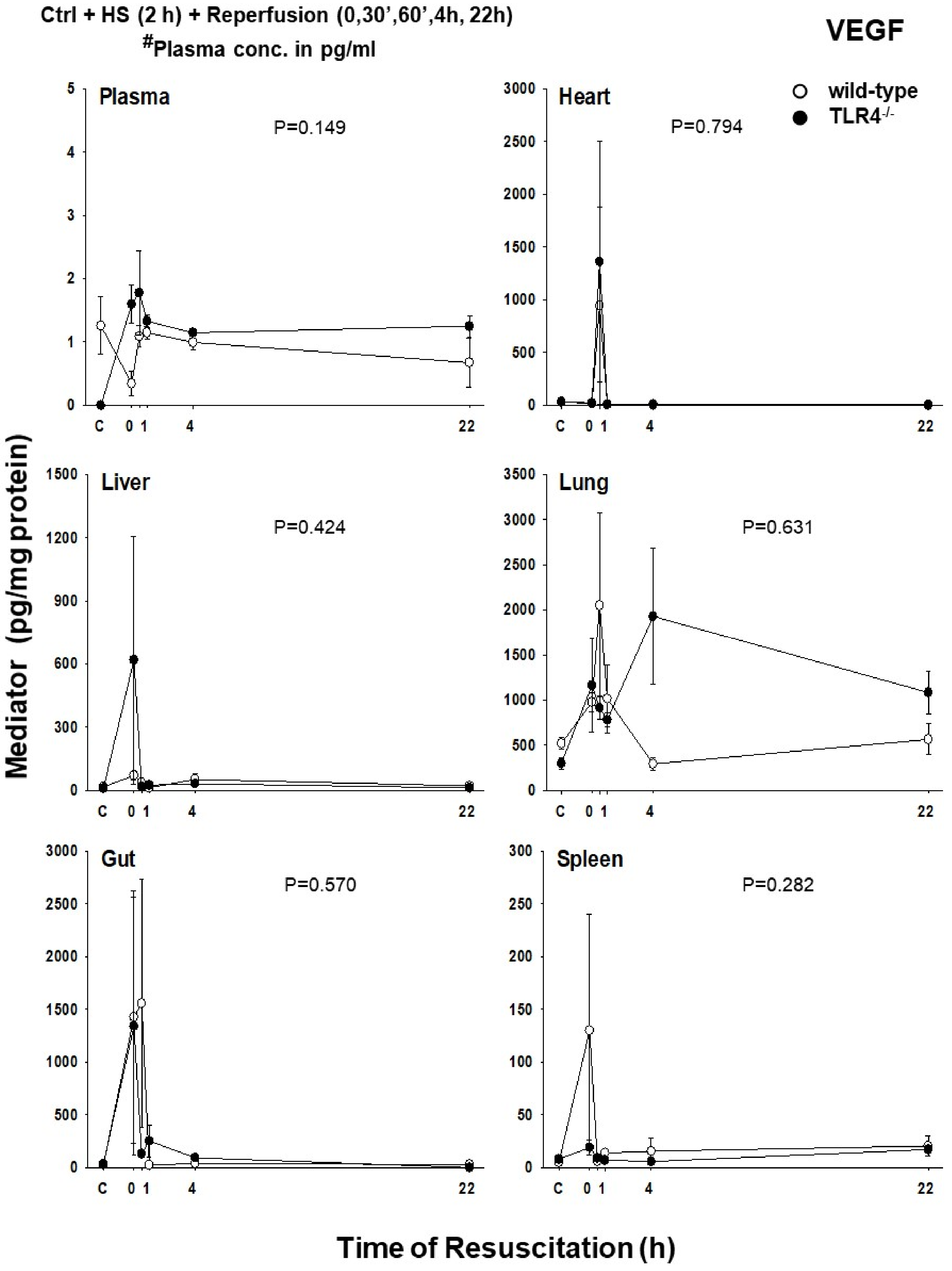

Ctrl + HS (2 h) + Reperfusion (0,30',60',4h, 22h)
#Plasma conc. in pg/ml
VEGF
wild-type
TLR4-/-
Plasma
P=0.149
Heart
P=0.794
Liver
P=0.424
Lung
P=0.631
Gut
P=0.570
Spleen
P=0.282
Mediator (pg/mg protein)
Time of Resuscitation (h)
C
0
1
4
22

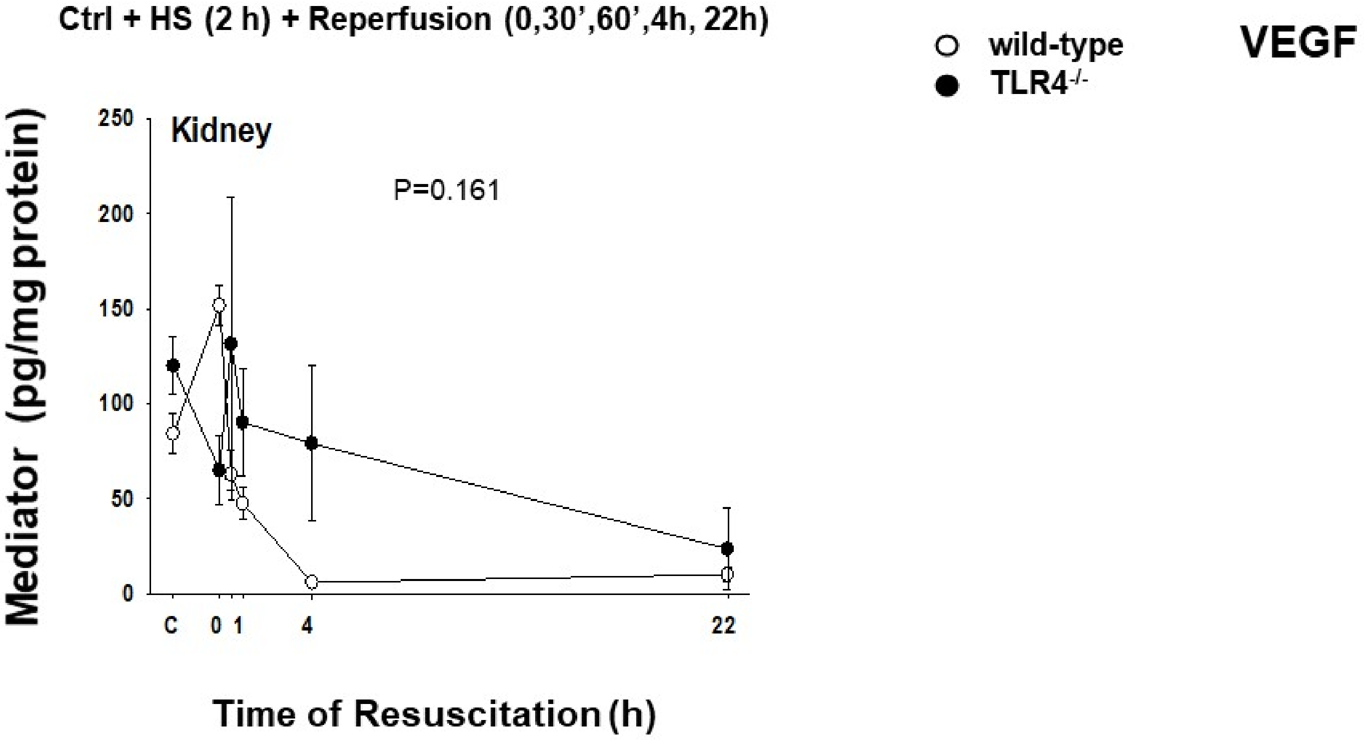
Ctrl + HS (2 h) + Reperfusion (0,30',60',4h, 22h)
wild-type
TLR4-/-
VEGF
Kidney
P=0.161
Mediator (pg/mg protein)
250
200
150
100
50
0
C
0
1
4
22
Time of Resuscitation (h)

Suppl. Table 1. Hypergraphs highlight compartmental differences between WT and TLR4$^{-/-}$ mice. Significant differences between WT and TLR4$^{-/-}$ mice exist primarily due to a greater number of inflammatory mediators present across many compartments in the WT mice compared to the TLR4$^{-/-}$ mice. If a cytokine is not present in the table, it is either located in the given compartment in both the WT and TLR4$^{-/-}$ mice or in neither the WT nor TLR4$^{-/-}$ mice.

	BL		0h		0.5h		1h		4h		22h	
	WT	TLR4$^{-/-}$	WT	TLR4$^{-/-}$	WT	TLR4$^{-/-}$	WT	TLR4$^{-/-}$	WT	TLR4$^{-/-}$	WT	TLR4$^{-/-}$
**Plasma**	IL-12p40, MIP-1a, IL-10, IL-1a, TNFa, IFN-g, IL-4, GM-CSF, IL-2, VEGF, IL-17A		MIP-1a, IL-17A	IL-1b	IFNg	IL-17A, VEGF, MIG	GM-CSF	IL-12p70, IL-13, IL-12p40	IL-12p40, IL-1b		GM-CSF, IL-17A, IL-1b, IL-5	IL-12p70, IL-10
**Kidney**	IL-5, IL-4, IL-12p70		IL-5			IL-5, IL-12p70, VEGF, MIG		IL-5		IL-5		IL-5
**Spleen**	IL-13, IL-5, IL-12p70			IL-5		IL-12p70, VEGF, MIG, IL-13		IL-5, IL-12p70, IL-13				
**Lung**	TNFa, IL-5, IL-4			IL-5	IL-5, IL-12p70	VEGF, MIG	IL-5, IL-10	TNFa			IL-12p40, IL-12p70, IL-13, GM-CSF, IL-17A, IL-1b	IL-5
**Liver**	IL-13, TNFa, IL-5, IL-4, GM-CSF, IL-17A, IL-12p70		IL-17A		IL-1b	VEGF, MIG			IL-17A	IL-5	IL-13, IL-12p70, MIP-1a, IL-17A, IL-1b, IL-5	
**Gut**	IL-5, IL-4, GM-CSF			IL-5	IL-5, IL-12p70	VEGF, MIG		IL-5, IL-12p70		IL-5, IL-12p70	KC	
**Heart**	IL-13, TNFa, IL-5, IL-4, GM-CSF, IL-17A, IL-12p70		IL-17A	IL-5, IL-12p70, IL-13		VEGF, MIG	GM-CSF, TNFa			IL-5	IL-12p40, IL-12p70, IL-13, GM-CSF, IL-17A, IL-1b, MIP-1a	
